# Data migration, validation and implementation of a new laboratory information system (LIS) in an academic pathology department, using Ellkay data archive, and Epic Beaker anatomic and clinical pathology modules

**DOI:** 10.1016/j.jpi.2025.100459

**Published:** 2025-06-25

**Authors:** Jeffrey Benitez, Adam An, Alec B. Santos, Amelia Flaus, Matt Wawrzyszko, Beverley Young, Eleanor Latta, Catherine J. Streutker, Ju-Yoon Yoon

**Affiliations:** a Department of Laboratory Medicine, Unity Health Toronto, Toronto, Ontario, Canada; b Department of Laboratory Medicine and Pathobiology, University of Toronto, Toronto, Ontario, Canada

**Keywords:** Laboratory information system, Epic, Beaker, Ellkay

## Abstract

Implementation of a new laboratory information system (LIS) poses a significant challenge, amplified when synchronous with launch of a new electronic medical record (EMR) system. Our institution made an executive decision to switch to Epic EMR and Epic Beaker LIS from Cerner Soarian/Altera Sunrise EMR and Cemer CoPath Plus LIS in anatomic pathology and molecular genetic pathology, with a simultaneous go-live date. This synchronous migration required a complete overhaul in our department of laboratory medicine, impacting all standard operating procedures (SOPs). In our efforts to minimize potential risks, we pursued a phased approach to comprehensive validation, starting with iterative rounds of optimization, ending with the final round of validation assessing 45 consecutive pathology cases, simulating the entire workflow in a dry-lab setting, from ordering to reporting, including addenda, with additional cases tested for specific workflow steps. In addition, we pursued validation of result component migration, in form of legacy pathology results to the Epic EMR, and the Ellkay archiving system. We found that our SOP adaptations for Epic Beaker reproduced >99% of the workflows previously established using CoPath Plus. The validation performed was limited to Epic Beaker LIS functionality, and, post-go-live, deficiencies were uncovered largely upstream of the LIS. Based on our experience, we formed a framework for systematic validation of LIS workflows, and share our comprehensive handbook, detailing all workflows built before go-live.

## Background

Hospitals rely on electronic medical record (EMR) and laboratory information system (LIS) technologies to record and distribute patient information, including charts and test results. In Ontario, Canada, EMR/LIS also connect hospitals to larger health system information repositories, including the Ontario-wide ConnectingOntario/Ontario Laboratories Information System (OLIS). Seamless transition of patient information between hospitals and ConnectingOntario/OLIS is required to maintain continuity of care when patients receive care at one or more of the 140 public hospital networks in Ontario, many of which have several sites. For example, a patient under investigation for cancer may undergo diagnosis and biopsy at one hospital in a network, biomarker testing at another hospital in the same network, and treatment at a site within a different hospital network. Ensuring that lab results are properly delivered in digital format across an individual a hospital site, across sites within a hospital network, and to ConnectingOntario/OLIS for distribution to other hospital networks is the responsibility of Ontario's labs.^1^

Our Division of Anatomic Pathology (AP) is responsible to deliver lab results within our hospital site, across our hospital network, and to ConnectingOntario/OLIS. Our lab is physically located in Toronto, Ontario, at St. Michael's Hospital (SMH). In 2017. SMH came together with St. Joseph's Healthcare Centre (SJHC), and Providence Healthcare (Providence) to form the three-site Unity Health Toronto network (Unity Health). Unity Health initially used CoPath Plus LIS (Cerner). CoPath Plus is an older LIS, first released in 1982,^2^ connected to two EMR systems used at two different hospital sites; Cerner Soarian used at the St. Michael's Hospital site, and Altera Sunrise used at the St. Joseph's Health Centre. In comparison to this legacy setup, newer LISs such as Epic Beaker feature functionality for better connectivity to OLIS, facilitated by the OLIS' HL7 FHIR® interface.^3^^,^^4^ The choice to migrate Unity Health to Epic was undertaken to improve Unity Health's connectivity within and among sites; to other hospitals within Ontario through data-sharing agreements,^5^ many of which use Epic; and to facilitate connectivity to ConnectOntario/OLIS.

Various institutions have described their Epic implementation experiences.^6^, ^7^, ^8^, ^9^, ^10^, ^11^ Challenges AP divisions can face when migrating to Epic EMR/Epic Beaker from legacy systems include: 1) How to manage the use of archived materials, tracked in the legacy system—transition plans need to consider that it is mandatory to retain human biological materials for defined time periods, mostly in form of formalin-fixed paraffin-embedded tissue blocks, for 10- and 20-year minimum retention periods in USA and Canada, respectively,^12^^,^^13^ which may be used for testing of biomarkers, including next generation sequencing (NGS) assays. 2) The legal requirement to ensure high-fidelity storage of pathology reports, where retention policy may span decades (e.g., autopsy reports). (3) Interfacing with other lab divisions, where tests may be ordered from pathology, such as tissue culture from autopsy specimens, and biochemistry tests (e.g., pancreatic fluid aspirates). 4) Armament of ancillary tests that may be performed within the pathology divisions, which may be traditionally be part of clinical pathology divisions, such as NGS, and cytogenetics assays (e.g., HER2 in situ hybridization).^14^, ^15^, ^16^ In oncology where biomarker testing continues to grow exponentially, and, at least partly reflecting this, human biological materials are associated with certain minimum periods of retention.^14^ Also, the use of various ancillary test calls for seamless interface between anatomic and clinical pathology (AP, CP) within single departments, which are often structured as separate divisions. Such logistics complexity calls for better integration of LISs and EMRs to enhance tracking of materials between divisions of lab medicine, and to ensure right testing is performed on the right specimens/materials.

Herein, we share our experiences in migration from a popular pathology EMR and LIS, Cerner Soarian/Altera Sunrise and CoPath Plus, respectively, to Epic, with the Epic Beaker LIS, and describe the framework we applied for validating the workflows before implementation. In comparison to these experiences, our AP division offered some additional challenges, where our test menu included molecular genetic pathology (MGP) tests, including NGS for somatic cancer profiling. Our legacy setup also lacked a designated LIS specifically for these MGP tests, with the workflow leveraging various tracking spreadsheet files, with the results reported using CoPath.^17^ With our goal of minimizing impact to patient care, we went to apply principles of validating pathology “tests”.^18^, ^19^, ^20^, ^21^ As our institution had customized the Epic Beaker workflows within Epic's foundation system to reflect the internal workflows, we benchmarked our institution's current, operational procedures as “gold-standard tests”, and formed our internal framework to validate the different Epic Beaker workflow steps to replicate the workflow functionality. Whereas our validation studies went well beyond the validation plan suggested by the vendors, allowing our department to confidently ensure functionality of key workflow steps within the AP division, our implementation and go-live experiences revealed several areas of deficiencies, largely reflecting the deficiencies in our validation scope. Besides sharing our validation framework, we also share our comprehensive handbook, which details all workflows built and validated before our go-live.

## Methods

### Customization of the institution's Epic Beaker

Our institution chose to implement the Canadian foundation system of Epic Beaker. During and before the clinical content validation phase 1 (CCV1), our institution worked with Epic representatives to collect information regarding how the institution's legacy workflows deviated from the Canadian foundation system of Epic Beaker, and customizations were applied in the POC (proof of concept). Numbering conventions differed. Our legacy systems used the convention SYY-#####-L (Y = year, # = number, L = letter), with alphabetical order of case parts, whereas the Epic Beaker workflow used numerical ordering of case parts. Another change was integration of ordering and receiving of all types of pathology tests into the single Epic/Epic Beaker system. Of the three Epic Beaker lab modules available to our institution—AP, CP, and molecular diagnostics—AP was the main module in which workflows were built, and the CP and molecular diagnostics modules called on for specific tests. The AP module was used for tissue exam tests, including accessioning and histology tasks, such as sectioning. The AP module was also used for certain ancillary tests, such as flow cytometry. Our flow cytometry build can be compared with the workflow utilized by Stanford, where they had utilized the Beaker CP module for their flow cytometry workflow.^10^ Other ancillary tests, including all molecular diagnostics, were built using the CP module.

### Legacy data migration

For all cases (from October/1988 to November/2024), all CoPath Plus (Cerner) data were extracted using Microsoft Structured Query Language, with patient ID (medical record number [MRN]), and case ID. The extracted data were migrated to the Ellkay archiving solution, an application separate from Epic. For pathology data spanning a 10-year period before go-live, reported results were extracted from the Soarian and Sunrise EMRs. The resulting data matrix was converted into HL7 format, and imported into the Epic EMR.

### Harmonization of “source & type” list, and interface with other epic applications

All ordering workflows, including other Epic modules, such as Epic OpTime (operating room, OR), Radiant (radiology), and Lumens (endoscopy), were built outside of the Epic Beaker workgroup, with no initial consultation with the laboratory medicine department. Rather, we provided a harmonized source and type (S&T) list of specimens submitted to our labs as the critical coupling link. The S&T of materials/specimens impacted the final destination(s) of the specimen within the lab, subsequent processing steps, transport methods, storage conditions, among other aspects. Linkage required us to: 1) Harmonize the S&T lists and LIS used by different divisions within laboratory medicine departments historically used different S&T lists, and two different LISs (CoPath Plus in AP and MGP *vs*. Soft in CP). Some “sources” also referred to not the source *per se*, but reflected the procedure, such as “fine needle aspiration”. Given that our AP division's ancillary test list includes CP tests, our S&T list required a department-wide harmonization (see Supplemental Materials).

For the AP division, billing and quality metrics acquisitions were directly tied to the S&T list, as this was tied to which protocol was triggered within CoPath Plus. Migration to the harmonized S&T list eliminated the capacity, and we had to build new processes. Automatic triggering of different protocols for histopathology, including printing of cassettes, were disabled; rather, protocols were entered at the time of specimen accessioning, after being received in AP.

### Validation

Our goals for migration were to ensure that go-live had the least impact on patient care possible, and to enhance tracking of materials/specimens between divisions of lab medicine, so the right testing was performed on the right materials/specimens. We accordingly applied principles of validating pathology “tests”.^18^, ^19^, ^20^, ^21^ We benchmarked our institution's current, operational procedures as the “gold-standard tests” against which we would validate the customized Epic Beaker workflow steps we developed to determine if they replicated our existing workflow functionality. The validation aimed to maintain the barcode-tracked workflow, previously established in the AP division,^22^ with the legacy setup in CoPath Plus. The “dry run” validation entailed placing of orders using the Requisition Entry function in Epic Beaker, noting the appropriate source and type of the specimens received. Subsequent steps in specimen accessioning was followed in Case Builder, manually selecting the appropriate protocol for the specimen, automatically inputting a set of various histology orders appropriate for the specimen (e.g., two H&E slides for endometrial biopsy). Grossing was simulated by creating all blocks for given cases, manually entering the gross description, and checking the cassette labels, barcodes, and colors. Histology was simulated by printing off slide labels. Downstream diagnostic work was simulated in Case Results, which include manually entering the diagnostic texts, synoptic reports, and ordering of additional ancillary workup, including histochemistry and molecular assays. Generated reports were checked in Chart Review. All validation work was performed using Epic Beaker, and, where feasible, all barcode-tracked steps were simulated using two different scanner models (Symbol DS4308, or Code Reader 2702).

## Results

### Legacy data migration and validation

Based on financial constraints, decision was made to migrate 10-years' worth of pathology data into Epic, with older data to be migrated to an integrated, but separate archiving solution, Ellkay. All legacy data were migrated from the hospital's two EMR systems, namely Soarian and Sunrise, rather than directly from the source LIS (CoPath Plus). The migration allowed for consolidation of patient records that spanned our two hospital sites; new MRNs were provided to all patients. Whereas this migration strategy worked relatively well, four main issues were identified: Initial validation strategy, as part of the institution's broader strategy was notable for pathology results that were not initially captured as part of the validation test set, related to the initial testing strategy, which focused on high-volume tests (e.g., electrolyte panel). We thus initiated two separate validation initiatives, one for the Epic EMR, and another for the Ellkay archiving system. The scope of the data migration strategies differed, where the migration to Ellkay required additional data elements, such as different patient demographics. Related to the data migration strategy to Epic EMR, referred-in test results of patients, who lacked MRNs from our institution were not captured. These cases included patients whose pathology cases were reviewed at our institution, before their physical arrival and being assigned a MRN, presenting a potential gap in available results.

Additional steps were also required for us to view AP data in Ellkay. We transferred mirrored data backup from CoPath Plus system, mapped the different data elements, and re-created different report templates. The process was generally simpler for migration of test results from Soarian/Sunrise to Epic EMR, where only the reported result elements were transferred. However, we observed that the institutions' initial data migration strategy did not transfer autopsy and supplemental reports. Once this issue was corrected, we validated the migration strategy. Overall, concordance between the data in the legacy systems and Epic EMR/Ellkay was 214/221 reports (96.8%). Table 1 summarizes the issues we observed in the remainder, and potential means to resolve them. Validation of details regarding different pathology asset information, critical for tracking,^22^ were deferred to validation at a later time.

**Table 1:** Deficiencies identified.

Deficiency noted	Description/Root cause	(proposed) Solutions
Improper/incomplete merging of charts from two hospital sites within institution	Discordant data points for single patients (e.g., different address) resulted in chart merging errors	Issue was flagged to the team managing patient registration. Manual review of specific charts was performed to observe the different IDs to be merged. However, as the migrated chart data were stored in different environments, actual merging of the charts could not be re-tested.
Missing test records	Some of the legacy test results not properly linked to a clinical encounter were not migrated, or the patient was not discharged within the legacy EMR.	Plan is to discharge all inactive encounters in the legacy EMR and re-migrate chart data afterwards.
Referred-in test records missing	Referred-in test patients were not assigned a MRN, but simply tested within the legacy LIS	Such missing reports were included as part of Ellkay, however, and the Ellkay migration validation included such tests.

We observed some additional data fidelity issues during migration; namely: 1) Inconsistent labeling among legacy pathology reports (i.e., “historical” title being applied only a subset of reports). 2) Heterogeneous categorization of pathology reports (e.g., “Gyn Cytology” cases categorized as “LAB BLOOD ORDERABLES”, and subset of non-Gyn Cytology cases categorized under “LAB FLUID ORDERABLES”. 3) Leading space at start of each line. 4) Records with for missing spaces between words. These issues were considered to be non-critical before go-live, with the categorization issue's potential impact on result filtering deferred to after the go-live date, during the implementation optimization phase.

### CP–AP interface–molecular ancillary tests

Our institution's menu of ancillary assays performed on pathology specimens included immunohistochemistry (IHC), immunofluorescence, flow cytometry (e.g., lymphoma protocol), electron microscopy (e.g., muscle biopsy), cytogenetics assays (e.g., HER2 FISH), and molecular assays (e.g., somatic NGS assay). Ordering of additional histology orders (e.g., histochemistry such as PASD) proved to be more straightforward tasks, reasonably setup within the foundation system. In comparison, ordering IHC ran into validation challenges, as the Epic-generated labels were found to be non-readable on our Ventana platform (BenchMark Ultra), an issue identified later in the validation. As a work-round solution, all IHC slides were double-labeled—first with Ventana-compatible label, followed by re-labeling of the slides with Epic-compatible labels.

For other tests, including flow cytometry, and molecular assays, managing of separate worklists, and managing associated data, such as workload capturing, proved to be difficult, with these aspects being tightly integrated within the larger Epic system. For cleaner, holistic management of these aspects of testing, these ancillary tests were built within the Beaker system to be separate tests, with their own case/specimen IDs and their own number wheels (e.g., F25–0001 for flow cytometry, and 25S–001D00001 for cytogenetics/molecular assays). This setup facilitated generation of discrete outstanding lists, where lab staff can easily visualize outstanding cases/specimens needing their attention.

**Fig. 1:**
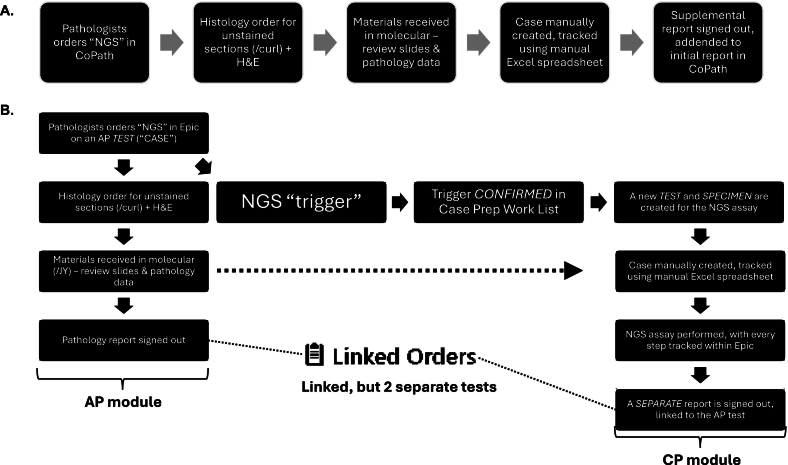
A) Legacy workflow for ancillary next generation sequencing (NGS) tests orders, i.e., somatic tumor testing. B) Epic Beaker workflow for ancillary molecular test orders. AP = anatomic pathology. CP = clinical pathology. “Trigger” order is placed as part of the NGS order protocol, confirming of which initiates a new test, with its own test identifier, with the test specimen being the materials received from surgical pathology.

To link the two, now separate, tests, we used “triggers” (Fig. 1). Within the Epic Beaker AP module, triggers were generally created as a virtual “level” from a block, distinct from the actual histology work performed on a case, such as unstained slides for DNA extraction. By using triggers, pathologists were able to order such linked ancillary tests within Case Builder, with or without associated histology orders (such as unstained slides for DNA extraction). This allowed additional tests to be ordered separately, such as adding on another molecular test to DNA already extracted for another test. We set up the triggers such that all triggers require manual confirmation to trigger the downstream reflex assay. For certain assay, it was appropriate for the histotechnologists to confirm the trigger, after grossly ensuring the presence of tissue on the unstained slide(s). For other assays, such as tumor NGS assays, the workflow was setup to be confirmed by the pathologist assessing the H&E slide for sufficient lesional tissue (e.g., 30% tumor for the *MGMT* methylation testing assay). This allowed us to track cases where the unstained slides have been cut, but not received by the molecular group. However, it made it challenging to create an outstanding specimen list, where the materials were not yet received by the MGP group.

Once the triggers were confirmed in Case Prep Work List, this lead to was creation of a new CP *specimen* and new test in Epic Beaker, linked to the AP case. Depending on viewing option used by the end users, linked results could still be viewed as a single set of results, with the molecular results displayed below the surgical pathology results. In comparison, all ancillary tests performed in our legacy system were reported as merely a supplemental report (addendum) to the original surgical pathology report, to form a single, large report. Whereas not flagged during validation, the linking of the reports did present an issue, which arose, for example, when reports were faxed to referring institutions, being sent as two separate reports, faxed at two different time points.

### Historical cases

Pathology labs have the unique requirement to active specimens/materials, and make them available for further testing, such as when a new biomarker is implemented (e.g., PD-L1). We thus investigated the migration of cases in CoPath Plus as actual *cases* to Epic Beaker, and examined how ordering additional workup on historic cases may be accomplished. Such migration strategy would be key to maintaining tracked AP workflow,^22^ which was previously established using CoPath Plus, with barcode scanning integrated. We found this to pose a significant mapping challenge. The minimal data element set was discussed to be: patient ID, case ID, procedure code, case type, specimen, specimen protocol, block, and slide. This migration would have required mapping of information to different master files within Epic (INIs), including MSH, PID, ORC, OBR, and ZCA. With some of the data elements not being readily available as data extracted from the legacy system, the risk/benefit analysis ruled against this strategy. As well, the financial constraints argued against this strategy, and thus the decision was made to only migrate the results into Epic (see above).

With result-only migration planned, we needed to devise a new workflow to: 1) Trigger the additional biomarker test, including NGS. 2) Track the flow of the pathology assets through the different steps of these additional biomarker tests. With the legacy LIS (CoPath Plus) to be sunsetted, the historical case workflow became essential for ongoing tracking in all steps, from (re-)accessioning to invoicing.

We formed a strategy for “historical cases”, where any cases requiring additional testing would be re-accessioned into Epic Beaker, under a separate historical number wheel (e.g., H25–00001), with the reports referring to the previously signed out case (see Supplemental Materials). Because various CP-built tests, including the somatic NGS assay, were resulted as separate CP reports, separate AP reports for the historical cases would be issued, linking the historic surgical case, and the new CP report(s). Clear advantage of this historical case workflow was to continue ongoing tracking of the AP assets in Epic Beaker, allowing the use of dashboards to specifically track these historical cases, whereas harmonizing the downstream CP testing workflows and outstanding case lists that included both historical and non-historical cases.

### Pathology workflow validation, deficiencies, and risk mitigation

Validation before go-live was performed in three phases of clinical content validation (CCV1–3). Printing of tissue cassettes and slide labels could not be figured out until merely 3 months before the go-live deadline. Numerous deficiencies were identified throughout the build and validation phases. Addressing deficiencies were generally challenging—whereas some were readily apparent, others required extensive consultation with those from both Epic and other institutions using Epic. Lack of familiarities with clinical workflows at the vendor levels was often identified, some of which were issues specific to our institution.

As the initial building and testing required numerous, iterative, rounds of fixes, and optimization, time restraints markedly restricted the opportunities for end-to-end workflow testing. However, our goal for migration was to ensure that we adapted our SOPs and workflows to reproduce existing functionality as closely as possible at go-live, to minimize impacts on patient care. We accordingly chose to pursue systematic validation, replicating all aspects of pathology testing for 45 consecutive cases from a typical workday in our department. The “dry-run” for these cases included any ancillary workup performed on the cases, including IHC, flow cytometry, and molecular testing. To ensure “accuracy” of the testing workflows, we included printing of the different LIS products, including specimen labels, slide labels, and cassettes. For any workflow/workflow steps with small sample sizes (e.g., molecular), additional, step-specific testing was performed (not included in the table). Related to coordination with the hematology division, aspects of hematopathology build, e.g., bone marrow order set, could not be validated before go-live. As well, various analytics function, such as building of dashboards for metrics monitoring, were not built and deferred for the optimization phase. Overall, >99% of workflow steps were simulated in the Epic Beaker system, without major deficiencies (Table 2).

**Table 2:** Workflow validation result summary.

	Workflow completed		Workflow NOT completed	% Workflow completed with no Major deficiency
	No deficiency	Minor deficiency		
Accessioning	45 (100%)	0	0	100%
Frozen section	2 (100%)	0	0	100%
Grossing	35 (79.5%)	8 (18.2%)	1 (2.3%)	97.7%
Histology	38 (92.1%)	3 (7.9%)	0	100%
Diagnosis	40 (97.5%)	1 (2.5%)	0	100%
Synoptic	2 (100%)	0	0	100%
IHC	19 (100%)	0	0	100%
Molecular	1 (100%)	0	0	100%
FISH	2 (100%	0	0	100%
EM	4 (100%)	0	0	100%
Flow cytometry	2 (66.7%)	1 (33.3%)	0	100%
Addendum	4 (100%)	0	0	100%
Other*	1 (100%)	0	0	100%

IHC = immunohistochemistry; FISH = fluorescent in situ hybridization; EM = electron microscopy; *Packing list function for transport of materials between two hospital sites.

All deficiencies noted are summarized in Table 3. One major deficiency was wrong color cassette being printed—this prompted re-review of the algorithm for the cassette hoppers in the printer. We addressed other more minor deficiencies with temporary bridging solutions at go-live, with resolution deferred to post-launch optimization. We could not assess certain aspects of our pathology workflows before go-live; in particular, ordering, because this function requires interfacing between Epic Beaker and different clinical applications within Epic (e.g., Epic Radiant for radiology). These processes are connected through different specimen navigation workflows which we could not test within Epic Beaker itself.

**Table 3:** Deficiencies identified and risk mitigation strategies.

Area/Workflow	Deficiency	Risk mitigation
Grossing	Wrong cassette color printed.	Cassette hopper selection workflow was re-reviewed and corrected.
Grossing	All cases start with default, undesired set of grossing text.	The default text can be easily deleted—fix deferred to optimization.
Grossing	“Print New” button behavior remaining active.	Pathology assistants prompted to check cassette printing behavior, rather than relying on this prompt.
Histology	Embedding instructions not apparent.	Leverage the “note” section within Case Builder.
Diagnosis	Beaker forced review of case by 2nd pathologist, preventing the primary pathologist from signing out.	Related to 10% quality control threshold being set in the foundation system—this function was disabled for go-live.
Flow cytometry	The flow cytometry test was not automatically linked to the cytopathology case.	At go-live, two tests will be manually linked. Review of auto-linking algorithm and fix were deferred to optimization.

Based on our experiences, we propose a framework for categorizing the level of evidence to gauge the ability of a LIS to complete a workflow (Table 4). Of note, the numbers of cases are restricted to cases are tested using the final, or the near-final version of the workflow. With ≥38/40 “accuracy”, the resultant point accuracy is 95% (confidence internal 83.5–99.4%), and we propose this cut-off as a reasonable compromise between confidence level and the limited time dedicated to full validation. Whereas larger sample sizes, and fuller validation would be ideal, many aspects of the custom-built system were constantly undergoing modification during our validation phases. During our validation, much of the workflows thus required re-testing due to various up- and down-stream processing steps being changed in the system. The time commitment for this phase should not be underestimated, based on our experience, due to tremendous need for re-testing, based on our experience.

**Table 4:** Proposed levels of evidence in LIS validation.

Level of evidence	(proposed) Description	Workflows meeting the level of evidence based on validation data
Level 1	Fully validated function, with at least 40 cases tested (“accuracy” ≥ 38/40)	• Accessioning • Diagnostic text entering, revision and verification (surgical and cyto-pathology)
Level 2	Validated function, with at least 20 cases tested (“accuracy” ≥ 18/20)	• Histology • Immunohistochemistry
Level 3	Limited number of cases tested (〈20) or “accuracy” < 18/20	• Grossing • Molecular genetic pathology • Electron microscopy • Flow cytometry • Addenda/Amendments • Autopsy

Based on validation activities performed from the pathology end, a handbook was formed, containing step-by-step instructions for different workflows, as a placeholder for temporary SOPs in preparation for go-live, and for better retention of institutional memory (see Supplemental Materials).

### Training and go-live

Technicians, technologists, and pathology assistants (PAs, also known as grossing assistants) were scheduled for a single session, taught by a microbiology technologist, unfamiliar with the pathology workflow. For example, PA-focused portion was limited to <0.5 h in length. Poor curriculum design and scheduling challenges even led to some trainees being released from their classes, upon reaching the trainer–trainee consensus of the class being irrelevant for the trainees' job descriptions. Training took place in the “ACE” environment, an environment that differed significantly from the production environment, due to ACE not being up to date with ∼2 month-lag in updates. Numerous workflow changes and deficiencies previously flagged, initially intended to be communicated during the training sessions, were thus not sufficiently communicated.

Our department addressed the need to augment our institution-led training by organizing small group sessions focused on job-specific training. These sessions comprised one to multiple focused session(s), with designated superuser(s) demonstrating different workflows relevant to different user groups. We produced a handbook, with step-by-step directions and screenshots outlining the steps of our division's various workflows (see Supplemental Materials). We made our handbook available to the entire AP division. We observed that some users printed the entire handbook for ease-of-use as they navigated the new workflows. In the frozen section rooms, where ready access to direction was needed, laminated hard copies were made available for the frozen section and lymphoma protocol workflows. A “lunch & learn” seminar was also held for the AP lab staff, providing an opportunity test out the system on their own and answer questions, before the system go-live.

Go-live took place on Nov 30, 2024, which soon revealed numerous deficiencies (Table 5). Among the first gaps we observed were related to staff training and to deferred testing of the Epic EMS/Epic Beaker order-test/case interfaces until after go-live. For example, OR nurses struggled to navigate the ordering system built through the specimen navigation route. We also noted some differences between the validated workflows in the TST (Test) environment from the actual go-live workflow in the live environment, including the grossing screen. During the first week of go-live, the most common inquiries received in pathology were regarding the ordering system, i.e., specimen navigation. Testing of different ordering workflows in the different clinical modules *outside* of Epic Beaker were not included in the Beaker LIS validation. Data for “integrated” testing, which focused on the EMR-LIS interface (i.e., clinical order/pathology case interface, i.e., order–test interface), were not available to the pathology division, and the different deficiencies in the ordering process were not appropriately flagged, resulting in cancelled tests, uncollected specimens, and wrongly ordered tests. Among the incorrectly ordered pathology tests were cases where each part of a single case was entered as an individual cases. Another was a set of prostate core biopsies, where a single descriptor was entered for the entire set, risking the loss of information regarding the different locations of the biopsied sites. We also received cases as a test that was not to be included in the test menu (“dermatopathology”), associated with its own case number wheel and block printing protocol, rather than the desired order as tissue exam test. Troubleshooting order issues was a challenging task for both the ordering parties and the lab group, as neither group was familiar with each other's interface. Even at go-live, various clinical orders needed major revisions We traced some of these ordering issues to an analyst using an incorrect version of the S&T list during the building process, resulting in some test options not appearing.

**Table 5:** Deficiencies identified post go-live, and their root causes.

Deficiency noted	Description/Root cause	(proposed) Solutions/Preventive strategy
Ordering – parts of a single pathology case being broken up into multiple cases	• EMR-LIS (i.e., order-to-test) interface could not be validated • Having 2 main tests for AP (“tissue resection” & “tissue biopsy”)	Validation of EMR-LIS interface, and better coordination with the analysts building various order sets
Source/type missing in the master list, preventing certain clinical teams from ordering tissue exam tests	• EMR-LIS (i.e., order-to-test) interface could not be validated	Validation of EMR-LIS interface, and better coordination with the analysts building various order sets
Orders placed not showing up in pathology worklist	• “Dermatopathology” was part of the foundation system, but not a recognized order in the customized version of Epic	Validation of EMR-LIS interface, and better coordination with the analysts building various order sets
Automatically cancelled orders	• Orders automatically cancelled upon patient being discharged and/or related to default time set for automatic cancellation, including perinatal autopsy orders	Modification of built-in timers for automatic order cancellation
Cases and task orders not visible to users within pathology	• Institution being setup as 2 geographical sites—orders from one site were not visible to the other • Delivery between sites require delivery of the “test” within Beaker	Clear SOP on login context to use for different user types, and integration of the worklists between sites as appropriate
Special stain, inc. IHC order not appearing on the task ordering panel	• Insufficient set of synonyms entered for the given task	More thorough review of synonyms was needed (e.g., cytokeratin 7 = CK7, cytokeratin-7, etc.)
IHC labels not readable on the Ventana platform	• Format incompatibility between platforms	All IHC slides were re-labeled using Epic labels after being stained as a bridging solution
Incomplete workflow – perinatal autopsy	• Order set initially built to be resulted within mother's chart	Accessioning of the fetus as a new patient, and completing the workflow therein.
Multiple molecular tests ordered on a single test	• Pathologists not recognizing that *each* trigger will reflex a molecular assay	Improved staff education
Molecular tests results not found in the EMR	• NGS results were not properly categorized in the result view tree to be under pathology	More thorough revie of the result view tree was needed
Insufficient data access and loss of quality monitoring abilities (e.g., TAT monitoring)	• Insufficient exposure and training for Cogito and related functions	Pathologist Builder program may enhance this aspect of lab management
Uploading of the results to the provincial LIS unsuccessful	• Incorrect accessioning and mapping of cases	Better coordination between the different Epic Beaker teams

Some of the issues were associated with informatics aspects of the workflows. For example, delivery of slides between two sites at our institution with a packing list resulted in some cases not appearing, based on a user's login context (i.e., SMH or SJHC site), and where the “test” is virtually held. At our institution, where majority of the histology and cytopathology screening work were performed at one location (SMH), it made sense to default the location of a given test to just that site during validation. A major deficiency was around the lack of analytics—building of various workbench reports for monitoring various quality metrics, including turnaround time, was not instituted before launch, and this deficiency remains unaddressed, 5 months after our go-live. In short, whereas our systematic validation approach allowed us to enter go-live with high level of confidence in functionality of the Epic Beaker LIS in the workflows tested, numerous deficiencies were noted outside the scope of the validation performed.

**Fig. 2:**
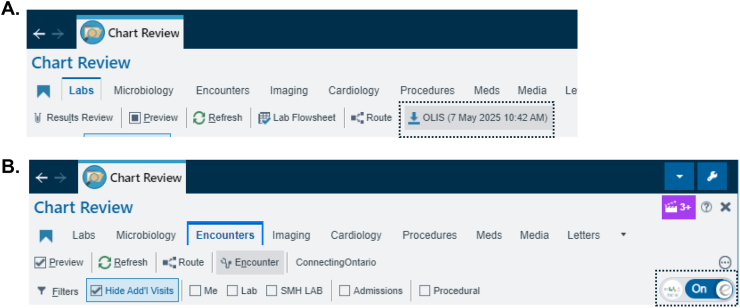
Evidence of improved connectivity. A) Screenshot of Chart Review tabs, which includes the “OLIS” button, which allows for downloading of results from OLIS for viewing. The date and time of last query are noted. B) In the “Encounters” tab, end users can view data from other institutions, and use the Toggle button to hide/show outside information.

Despite its challenges, our migration to Epic EMR/Epic Beaker is an important milestone, potentially marking our foray into pathology informatics. An obvious benefit of migration was also evident in end users' abilities to: 1) Readily download data from OLIS and view the results within the Chart Review view. 2) View data from other hospitals in Ontario directly (Fig. 2).

## Discussion

Concerted migration of our legacy Soarian/Sunrise EMR and CoPath Plus LIS to the Epic EMR/Epic Beaker platform has some obvious advantages, especially regarding interfacing of ordering, testing, resulting, and viewing of the results of a given lab test. These benefits were echoed in survey at other institutions of early adopters of Epic Beaker AP.^9^ Conversion of data to a standardized format will facilitate sharing of data across institutions, reducing loss of data fidelity related to faxing/photocopying artifacts, with OLIS, and reduce loss of data fidelity related to faxing/photocopying artifacts. As well, the Cogito module opens doors for extensive pathology data analytics, opening doors for facilitated implementation of pathology informatics.

**Fig. 3:**
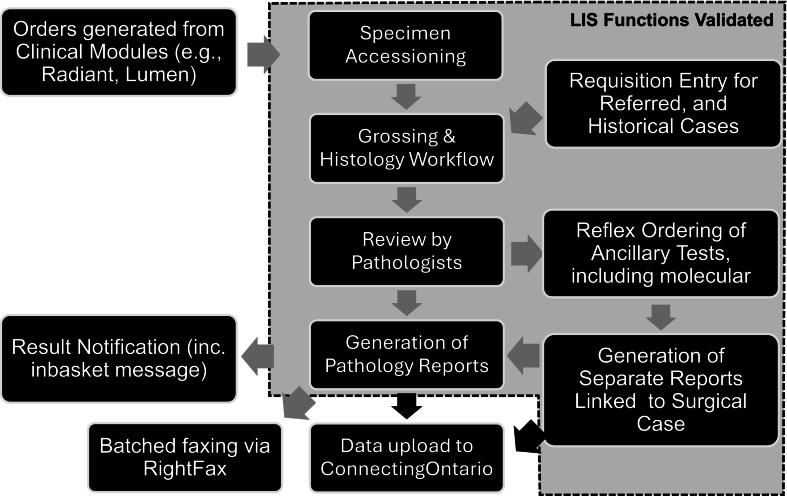
Graphical depiction of some of the key workflows in the department, distinguishing the workflows/steps that were assessed during validation, before go-live (dotted lines). Functions outside the dotted area represent those built by a separate team and represent interfaces that were not tested during validation. Black arrows indicate steps where the data undergo conversion to HL7 before data upload.

However, some challenges unique to the LIS, especially to the pathology LIS, were overlooked in the process of planning the migration to the new systems. We suggest that many of these challenges were preventable. Appropriate engagement and consultation with lab pathology departments during testing of clinical workflows that integrate Epic EMR/Epic Beaker could have minimized the go-live issues encountered on the upstream, Epic EMR side of the workflow—like the lab test ordering processes. We suggest that a team approach would be more effective during the customization/building phases than a siloed approach, where independent groups of analysts built workflows in each clinical module, using only the S&T list connect their work. Such siloed processes resulted in orders not appropriately leading to the desired test(s), separately built by the Beaker teams. Our experience indicates that workflows bridging the Epic EMR/Epic Beaker interface were the most vulnerable, yet were not robustly tested before go-live, including being out of scope for the Epic Beaker validation we were tasked to complete (Fig. 3).

Improved training of the nursing and technical staff members on the clinical and lab sides of orders, respectively, may also be an area where migrations could be improved. After go-live, it was generally assumed that all issues with ordering arose from Epic Beaker, and it fell to lab staff to provide training on how to order AP tests. In reality, the issues arose from both sides of the ordering system, and lab staff were not trained on, or familiar with, the ordering interface in the Epic clinical modules seen on the ordering side. Rather, the environment provided to us for validation entered cases with the “Requisition Entry” function, which did not simulate the different specimen navigation pathways outside of Epic Beaker (e.g., Epic OpTime, Epic Radiant, and Epic Lumens). Future validation strategies should include testing of the Epic EMR/Epic Beaker interfaces before go-live.

Other interfaces also represented points of vulnerabilities. Within the AP module, for example, the customization of the module to reporting was a relatively smooth process. However, the interface between the AP module with equipment (e.g., Ventana IHC platform) proved to be challenging related to incompatible barcodes. The AP–CP interface, relevant for different molecular assays ordered for biomarkers, was a particularly difficult one, delaying the building of the assay within the systems, and taking advantage of more advanced functions, such as Batch Editor (for designating 96-well plates) and different quality tracking functions. The use of “triggers” for initiating various molecular orders also provided challenge in pulling data for such orders for tracking and dashboard data review purposes.

Building/customization of institution-specific workflows, apparently from a relatively basic “foundation” system, without the knowledge of other institutions' approaches, posed a significant challenge. When the authors were tasked with customizing the AP workflow, knowledge of different functions possible was limited to select individuals who had used Epic elsewhere. The AP workgroup provided various sets of data reflecting how some workflows are performed within our institution, including different preferences on how something may be performed in Epic. However, it was generally unclear how those selections and data may shape the workflows to be built, with some repercussions only realized post go-live. As well, several aspects of the build were further challenged by the intrinsic differences between the US and the Canadian healthcare systems. Anecdotally, the concept of the public insurance plan, i.e., Ontario Health Insurance Plan was found to be a foreign concept that persisted as foreign, even well near the completion of the initial building of our test environment. The analysts also needed repeated reminders on differences in billing practice; unlike our US colleagues, reviewing of the charges prior signing out a case is not a typical part of daily practice in surgical pathology in Canada. Rather, at our institution, the workload is tracked by the administrative staff members for reimbursement from the government body(ies) and for personnel management purposes.

**Fig. 4:**
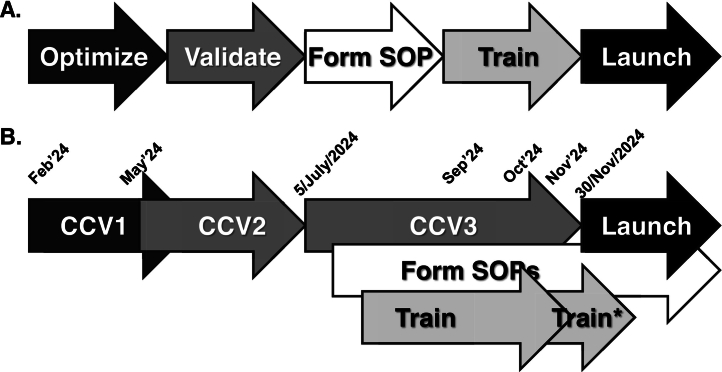
A) Ideal roll out optimization, validation, and roll-out of LIS workflows. B) Epic Beaker roll out at Unity Health Toronto. CCV = clinical content validation (phases 1–3). *additional job-specific training provided internally. The concerted go-live of the Epic electronic medical record system took place on Nov 30, 2024, along with Epic Beaker, the lab information system application.

The go-live was further challenged, related to the challenges in testing/validation, and training. In lab medicine, validation of an assay, or assay component, is performed to ensure performance as intended, with related quality metrics, as guided by various regulatory bodies (e.g., www.iso.org and www.clsi.org). In attempting to validate different aspects of Epic Beaker, all aspects of the workflows were constantly in flux, even up to the point of the go-live date. The ideal sequence of: 1) optimize the workflow, 2) validate the workflow, 3) form SOPs around validated workflow, supported by the validation data, and 4) train staff members and form operations accordingly (Fig. 4). Rather, we witnessed numerous issues being identified related to insufficient testing, especially integrated end-to-end testing, having to form bridging SOPs and work-round solutions on the fly. Numerous issues were identified throughout the building and validation exercise, which were left “to be addressed during training”. This strategy was not optimal, because the trainers being unaware of the issues being encountered by end users.

Our experience with reproducing and validating our legacy workflows did have the advantage of providing us with a confident understanding of Epic Beaker itself. We collected screenshots throughout the validation, and used these and our experience to produced a handbook with a temporary set of SOPs. We are now using these to update our SOPs in Paradigm Software, a work management processor. Better understanding gained through our validation also facilitated troubleshooting, and allowed us to determine that most issues arose upstream of any validated Epic Beaker workflow. Whereas we are not positioned to perform head-to-head comparison of different systems available in the market, our comparison with the institution's legacy setup is summarized in Table 6. The migration to the Beaker LIS led to different harmonization endeavors, between different divisions within the department of laboratory medicine, where the CP divisions, excluding molecular pathology, used Soft as their legacy LIS. Migration to Beaker required all divisions, AP and CP, to use a single, harmonized S&T list, for the purpose of building the different clinical workflows. Transfusion was another exception, where an appropriate solution had to be found outside of the Beaker system (WellSky), the validation efforts for which were directed by a different team. Better integration with HL7, compared to our older LIS, positioned our department for better data sharing with other institutions.

**Table 6:** Comparison of the Epic system to the legacy systems.

Advantages (vs. legacy setup)	Disadvantages
• Better integration with HL7 • Integration of multiple hospital sites • Single LIS system use for department • Harmonized “source and type” list • Integration of pathology LIS and clinical EMR, allowing for faster chart review • Migration to all-electronic ordering system from paper requisitions • Facilitated result exchange with other institutions also using Epic • Integration of OLIS/Connecting Ontario • Facilitated access to data analytics modules through Cogito	• Need for a separate LIS for transfusion (WellSky) • Numerous work-round solutions needed to be devised • Steep learning curve for various data analytics, including that for quality monitoring • Loss of flexibility in various workflow management, including wrongly placed orders • Separation of ancillary pathology results from the pathology cases • Apparent lack of time-stamped reports • Greater dependence on the IT team for resolution of any issues, including from wrong patient demographics

Obvious disadvantages and challenges include those secondary to insufficient testing before the launch, as discussed above. The temporary solutions we needed to apply may impact our division further in the future, including the fidelity of data exported for different analytics purposes. Assuming eventual resolution of such issues, another disadvantage is related to loss of AP's abilities to troubleshoot Epic Beaker-related issues. Migration to Epic translated to more intimate integration between different aspects of testing, including billing. As such, resolutions of numerous issues required submitting a ticket to the information technology team, where some tickets added weeks to a case's turn-around time in some cases. Various analytics-related functions, such as turnaround time monitoring, were not in place before go-live. Whereas challenges of forming such workflows before go-live with only test data are acknowledged, addressing this deficit post go-live has proven to be markedly labor-intensive.

In our efforts to help other institutions with this challenging transition, we share our handbook that was formed during our validation. The handbook, for our group, represented aggregated, temporary SOPs, meant to bridge our group during the transition. Every institution is likely to be unique in terms of the minute details of their workflows, necessitating customization of the Epic Beaker system, and our handbook may not be directly applicable. However, the knowledge we gained on how to approach may help institutions to be better prepared as they undertake a transition to Epic. We also propose a conceptual framework to validate LIS workflows to lower risks when migrating from legacy to new LIS. We also suggest that involving pathologists and other physicians and pathologists earlier in the process, including in physician/analyst pathologist/analyst teams during workflow builds could enhance interdepartmental knowledge of the system, and potentially circumvent some of the interfacing issues and vulnerabilities we encountered with the more siloed approach. It is clear from our experience that validation must be extended to various interfaces, including the ordering, and the lab workflows. Involvement of the clinical and lab sides in testing such interfaces would be beneficial, as would inclusion of a greater range of stakeholders in the building process.

## Ethics statement and patient consent

Not applicable.

## Funding

Parts of Molecular workflow building and validation were supported by quality improvement grants from Amgen and Pfizer.

Parts of this study were funded by Amgen (PI = JY), and Pfizer (PI = JY).

## Declaration of competing interest

The authors declare the following financial interests/personal relationships which may be considered as potential competing interests:

Ju-Yoon Yoon reports a relationship with Amgen Canada Inc. that includes: funding grants and speaking and lecture fees. Ju-Yoon Yoon reports a relationship with Bayer Corporation that includes: funding grants. Ju-Yoon Yoon reports a relationship with AstraZeneca R&D Reims that includes: funding grants. Ju-Yoon Yoon reports a relationship with Roche that includes: consulting or advisory. Ju-Yoon Yoon reports a relationship with Merck Sharp & Dohme Corp that includes: funding grants. Ju-Yoon Yoon reports a relationship with Pfizer that includes: funding grants. If there are other authors, they declare that they have no known competing financial interests or personal relationships that could have appeared to influence the work reported in this article.

## Appendix A Supplementary data

Supplementary material Unity Health Toronto Epic Beaker handbook for the division of anatomic pathology. Supplementary material

## References

[1] ConnectingOntario ClinicalViewer. https://ehealthontario.on.ca/en/health-care-professionals/connectingontario.

[2] Aller R.D., Weiner H. (2013). Anatomic pathology computer systems. CAP Today.

[3] eHealthOntario (2019). Ontario Laboratories Information System HL7 FHIR® Consumer Query Implementation Guide-v1.0.1. https://simplifier.net/guide/OntarioLaboratoriesInformationSystemConsumerQuery/Introduction?version=current.

[4] eHealthOntario Ontario eReferral – eConsult – HL7® FHIR® Implementation Guide. https://ehealthontario.on.ca/en/standards/ontario-ereferral-implementation-guide-fhir-overview.

[5] Dong L., Sahu R., Black R. (2022). Governance in the transformational journey toward integrated healthcare: the case of Ontario. J Inf Technol Teach Cases.

[6] Krasowski M.D., Wilford J.D., Howard W. (2016). Implementation of Epic Beaker clinical pathology at an academic medical center. J Pathol Inform.

[7] Blau J.L., Wilford J.D., Dane S.K. (2017). Implementation of Epic Beaker anatomic pathology at an Academic Medical Center. J Pathol Inform..

[8] Tan B.T., Fralick J., Flores W. (2017). Implementation of Epic Beaker clinical pathology at Stanford University Medical Center. Am J Clin Pathol.

[9] VanSandt M., Turner K., Dash R. (2020). Pathologist opinions about EPIC beaker AP: a multi-institutional survey of early adopters. J Med Syst.

[10] Oak J., Gitana G., Wei S., Parry M., Tan B. (2025). Implementation of beaker CP for flow cytometry: workflow optimization and integration at Stanford Health Care. Cytometry B Clin Cytom.

[11] Miller M., Bow L.M. (2018). P039 the bumpy road to epic beaker implementation. Hum Immunol.

[12] Torlakovic E.E., Riddell R., Banerjee D. (2010). Canadian Association of Pathologists-Association canadienne des pathologistes National Standards Committee/Immunohistochemistry: best practice recommendations for standardization of immunohistochemistry tests. Am J Clin Pathol.

[13] Pathologists CoA Minimum Period of Retention of Laboratory Records and Materials. https://www.cap.org/gated-assets/uploads/private/cap-retention-laboratory-records-and-materials.pdf.

[14] Begum N.F., Ramalingam K., Ramani P. (2024). Storage, retention, and use of leftover pathology specimens: the underestimated treasures. Cureus.

[15] Yao J., Zhai Q. (2022). A narrative review of cancer molecular diagnostics: past, present, and future. J Bio-X Res.

[16] Pathologists CoA (2023).

[17] Shen S., Yoon J.Y. (2025). Custom R Flexdashboard for molecular genetic pathology quality tracking. J Pathol Inform..

[18] Jennings L.J., Arcila M.E., Corless C. (2017). Guidelines for validation of next-generation sequencing-based oncology panels: a joint consensus recommendation of the Association for Molecular Pathology and College of American Pathologists. J Mol Diagn.

[19] Jennings L., Van Deerlin V.M., Gulley M.L. (2009). Recommended principles and practices for validating clinical molecular pathology tests. Arch Pathol Lab Med.

[20] Chen B., Richards C.S., Wilson J.A., Lyon E. (2011). Quality assurance and quality improvement in U.S. clinical molecular genetic laboratories. Curr Protoc Human Genet.

[21] Pum J., Makowski G.S. (2019). Advances in Clinical Chemistry.

[22] Pantanowitz L., Mackinnon A.C., Sinard J.H. (2013). Tracking in anatomic pathology. Arch Pathol Lab Med.

