# Supplementary material for: Data migration, validation and implementation of a new laboratory information system (LIS) in an academic pathology department, using Ellkay data archive, and Epic Beaker anatomic and clinical pathology modules
Source: J Pathol Inform. 2025 Jun 25;18:100459. doi: 10.1016/j.jpi.2025.100459 (PMC12332207; doi:10.1016/j.jpi.2025.100459)
Supplement: Supplementary material — Unity Health Toronto Epic Beaker handbook for the division of anatomic pathology. [file mmc1.docx]

# PATHOLOGY EPIC BEAKER INSTRUCTION MANUAL

[PATHOLOGY EPIC BEAKER INSTRUCTION MANUAL 1](#_Toc189650518)

[WORKFLOWS IN PATHOLOGY – General Notes 5](#_Toc189650519)

[Logging in & logging out 5](#_Toc189650520)

[Menus & Outstanding List 6](#_Toc189650521)

[Scanners 7](#_Toc189650522)

[Scanners – Symbol Barcode Scanner DS4308 8](#_Toc189650523)

[Scanners – Zebra DS46 Barcode Scanner 9](#_Toc189650524)

[Finding Patients (inc. Chart Review) 10](#_Toc189650525)

[Ordering (inc. Marking STAT orders) 10](#_Toc189650526)

[Orders – Linked Orders 12](#_Toc189650527)

[Specimen Collection 12](#_Toc189650528)

[Specimen Collection – Infectious specimen documentation/handling 13](#_Toc189650529)

[Specimen Collection – Scheduled Collection (ROSE, EBUS, Bone Marrow), using Snapboard 15](#_Toc189650530)

[Specimen Receiving – Rejecting Specimens 15](#_Toc189650531)

[Specimen Transport – inc. Send-out testing & Packing list editor 16](#_Toc189650532)

[Specimen Transport – Send-out Tests 18](#_Toc189650533)

[Accessioning (& Case Receiving) 20](#_Toc189650534)

[Accessioning – Lab Requisition Entry 21](#_Toc189650535)

[Accessioning – Consultation (“referred-in”) cases 22](#_Toc189650536)

[Accessioning – Historical case workflow 25](#_Toc189650537)

[Accessioning - Container sharing 27](#_Toc189650538)

[Finding Cases 27](#_Toc189650539)

[Case Builder 30](#_Toc189650540)

[Case Builder – Editing Case Information 31](#_Toc189650541)

[Case Builder – Marking as Frozen 32](#_Toc189650542)

[Case Builder – Assigning cases 34](#_Toc189650543)

[General – QA/QC/QM 35](#_Toc189650544)

[General – Add Follow-up Task 36](#_Toc189650545)

[General – Flagging cases, specimens 39](#_Toc189650546)

[General – Downtime Procedures 39](#_Toc189650547)

[General – Smart texts and smart phrases 40](#_Toc189650548)

[General – Useful SmartLinks 40](#_Toc189650549)

[General – Using Dragon 41](#_Toc189650550)

[General – Billing & Charge Codes 41](#_Toc189650551)

[GROSSING AND HISTOPATHOLOGY 41](#_Toc189650552)

[Grossing 41](#_Toc189650553)

[Grossing – Gross-only cases, inc. foreign body 42](#_Toc189650554)

[Grossing - Adding/Removing Blocks 43](#_Toc189650555)

[Grossing – Confirming Blocks 43](#_Toc189650556)

[Grossing – (Re-)Grossing post sign-out 44](#_Toc189650557)

[Grossing – Adding Flags, including cold ischemic time 45](#_Toc189650558)

[Histology – Case Prep Work List (microtomy) 46](#_Toc189650559)

[Histology – Tracking 47](#_Toc189650560)

[EM (electron microscopy) 49](#_Toc189650561)

[IF (Immunofluorescence) for Kidneys and Skin Specimens 51](#_Toc189650562)

[REPORTS (inc. Sample report) 53](#_Toc189650563)

[Reports – Viewing charts, results & reports 54](#_Toc189650564)

[Reports – Result Review 55](#_Toc189650565)

[Reports – Faxing Reports 56](#_Toc189650566)

[Reports – MyChart 57](#_Toc189650567)

[Reports - Beaker Laboratory Report Routing 58](#_Toc189650568)

[Reports – Adding Recipients (Route) 58](#_Toc189650569)

[Reports – OLIS Interface 59](#_Toc189650570)

[Reports – Connection Ontario 60](#_Toc189650571)

[COMMUNICATION 61](#_Toc189650572)

[Communication – Critical Results & Comm log 61](#_Toc189650573)

[Communication – In-basket message 62](#_Toc189650574)

[Communication – Secure Chat 62](#_Toc189650575)

[ANATOMIC PATHOLOGY (AP) Workflows 63](#_Toc189650576)

[AP - Intraoperative Consultation (inc. Frozen Section) 64](#_Toc189650577)

[AP – Gross-Only Cases (foreign bodies) 68](#_Toc189650578)

[AP – Ordering Histochemistry & IHC (Task Protocols) 69](#_Toc189650579)

[AP – Ordering Tasks on Previously Cut, Unstained Slides 70](#_Toc189650580)

[AP – Resulting 71](#_Toc189650581)

[AP – Viewing Linked and “Other” Results 73](#_Toc189650582)

[AP – Synoptic Form (Report) 73](#_Toc189650583)

[AP – Synoptic Report using Macro 75](#_Toc189650584)

[AP – Verify Results (sign-out) 76](#_Toc189650585)

[AP – Residents and other Trainees, including Fellows 77](#_Toc189650586)

[AP – Addenda and Amendments 77](#_Toc189650587)

[CYTOPATHOLOGY 81](#_Toc189650588)

[Cytology – Cytotech Workflow 82](#_Toc189650589)

[Cytology – Cytopathologist Workflow 94](#_Toc189650590)

[Cytology – Ordering and MLA Workflow 113](#_Toc189650591)

[Cytology – GYN cytology 125](#_Toc189650592)

[Cytology – Pap/Gyn Order Composer Questions 126](#_Toc189650593)

[Cytology – Non-Gyn Order Composer Questions 126](#_Toc189650594)

[Cytology – FNA Order Composer Questions 126](#_Toc189650595)

[Cytology – Second Pathologist Review 127](#_Toc189650596)

[Cytology – Sendout HPV test 129](#_Toc189650597)

[AUTOPSY Workflow 132](#_Toc189650598)

[Autopsy – Body Tracking 133](#_Toc189650599)

[Autopsy – Provisional Report 134](#_Toc189650600)

[Autopsy – Final Report 135](#_Toc189650601)

[Autopsy – Ancillary Workup 135](#_Toc189650602)

[HEMATOPATHOLOGY (HP) Workflows 136](#_Toc189650603)

[HP – Peripheral blood review 136](#_Toc189650604)

[HP – Bone Marrow Panel 137](#_Toc189650605)

[HP – Flow Cytometry 140](#_Toc189650606)

[HP – Lymphoma Protocol 142](#_Toc189650607)

[MGP (MOLECULAR GENETIC PATHOLOGY) 142](#_Toc189650608)

[MGP – General 142](#_Toc189650609)

[MGP – Accessioning & Receiving 143](#_Toc189650610)

[MGP – Batch Editor 144](#_Toc189650611)

[MGP – Repeating steps/assays 146](#_Toc189650612)

[MGP – FISH, HER2 147](#_Toc189650613)

[MGP – FISH, 1p/19q 150](#_Toc189650614)

[MGP – DNA extraction 152](#_Toc189650615)

[MGP – *MGMT* promoter methylation assay 152](#_Toc189650616)

[MGP – *MLH1* promoter methylation 154](#_Toc189650617)

[MGP – JAK2 p.V617F assay (MutaScreen) 155](#_Toc189650618)

[MGP – PRMU/FVL 156](#_Toc189650619)

[MGP – NGS (UHT somatic tumour molecular profiling assay) 158](#_Toc189650620)

[MGP – In-house microbiology testing 160](#_Toc189650621)

[MGP – Sendout molecular tests 161](#_Toc189650622)

[MGP – QA/QC/QM 162](#_Toc189650623)

[USER SETTINGS 162](#_Toc189650624)

[User Settings – General 162](#_Toc189650625)

[User Settings – Creating SmartPhrases 163](#_Toc189650626)

[User Settings – Creating new lists (EMR function) 164](#_Toc189650627)

[SUPPORT RESOURCES 166](#_Toc189650628)

[Support Resources – Help Resources 166](#_Toc189650629)

[Support Resources – Tickets 167](#_Toc189650630)

[Support Resources – PLY (playground) environment 167](#_Toc189650631)

[ELLKAY 168](#_Toc189650632)

[Ellkay – General 168](#_Toc189650633)

[Ellkay – Viewing results 170](#_Toc189650634)

[Ellkay – Additional information 171](#_Toc189650635)

[Trainees 171](#_Toc189650636)

[ANALYTICS 171](#_Toc189650637)

[Analytics – Pathologist Dashboard 171](#_Toc189650638)

[Analytics – SlicerDicer 172](#_Toc189650639)

[Analytics – OR list 173](#_Toc189650640)

## WORKFLOWS IN PATHOLOGY – General Notes

### Logging in & logging out

| 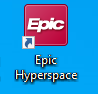 | Open Epic by double-clicking on the Epic icon in the desktop. Login information matches your UHT PC login parameters.  - NOTE: Be sure to choose the “live” (PRD) instance of Epic Hyperspace – other options may be available for training purposes |
| --- | --- |
| 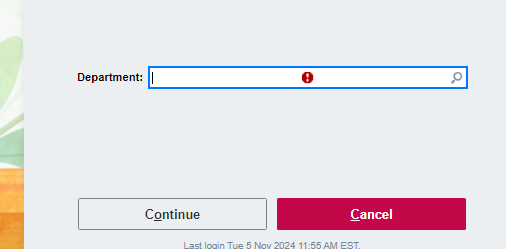 | Users will be prompted to choose a “Department”:   - “SMH lab” - for users physically located SMH - “SJHC Lab” - for users physically located at SJHC |
| 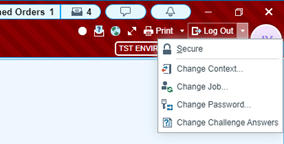 | Users can login with “SJHC lab” instance. Instances can be switched within Epic by clicking on your initials (top right corner) and clicking “Change Context”. |
| 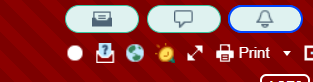 | Various messages (in-basket & secure chat) & Notifications are available   - Secure chat – easily attach patient to chat, can chat with multiple people. |
| 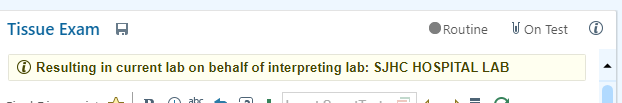 | Resulting report will reflect with different headers. Header is determined by collection location. You may notice certain notification messages, also reflecting the different lab contexts. |
| 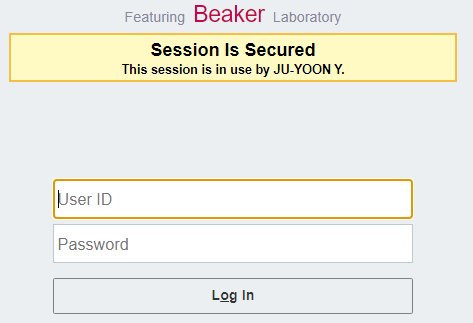 | 10 minutes of inactivity will “secure” the session as shown.   - If in the middle of a case, the information should have been saved and user exited out of a case. |
| 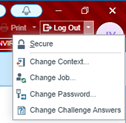 | Session can be manually secured by clicking on the arrow button and clicking “Secure”. |

### Menus & Outstanding List

| 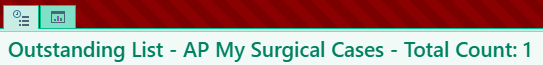 | Default start-up activities (ex. Outstanding list) - set by institution, based on role   - Outstanding list is the landing page for most pathology staff members |
| --- | --- |
| 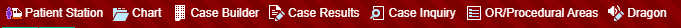 | Main tool bar (patient station, chart, etc.) - user customizable  - items can be pinned - others available through Epic tool bar or search bar  - search - can find activities & can search within chart |
| 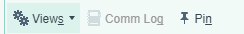 | Views to access other various lists |
| 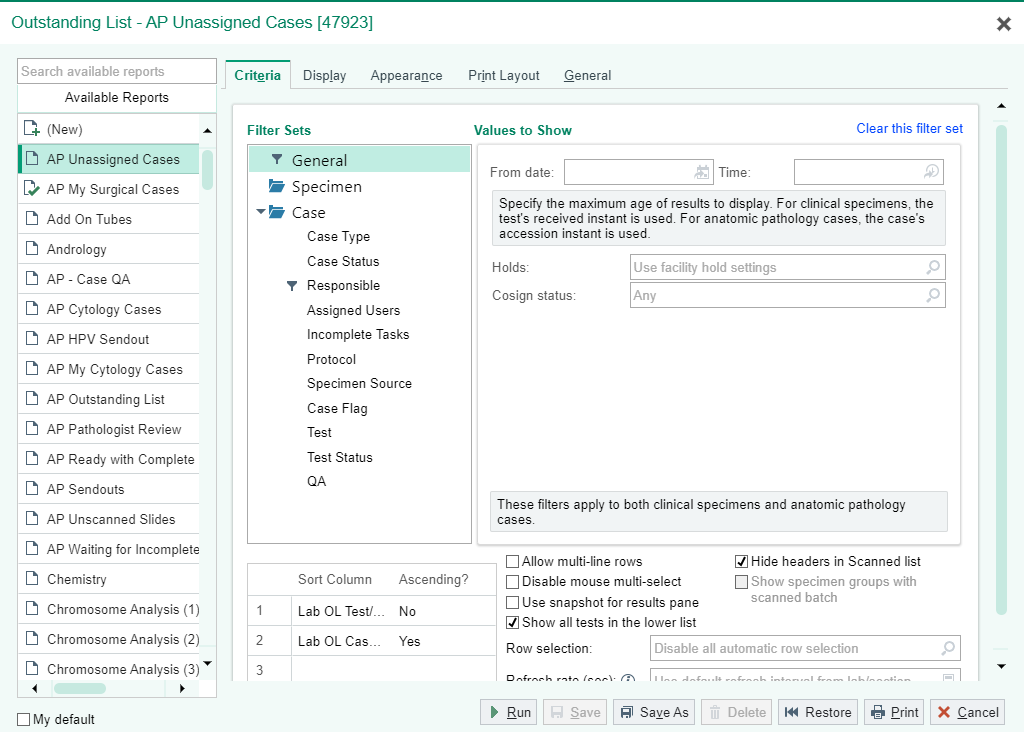Outstanding lists can be edited – click on Views > Settings   - Lists can be set as “My Default”, saved and run | |

NOTE: The outstanding list views cannot be filtered.

### Scanners

Scanners require setup to be compatible with both Epic and Cerner (CoPath). Two different sets of instructions are available—one for the Symbol brand, another for the Zebra brand.

| Symbol DS4308 | | Zebra DS46 | |
| --- | --- | --- | --- |
| 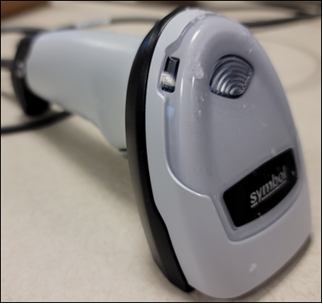 | 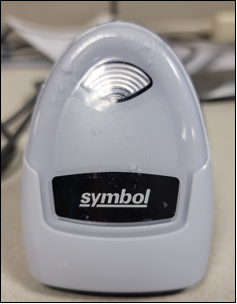 | 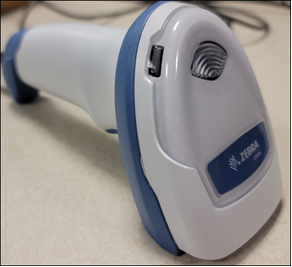 | 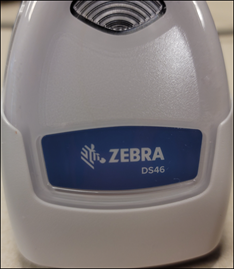 |

Also available are Code barcode scanners (CR2700), which should be setup for Epic by default setup. If not setup, a QR code should be available on the scanner body unit.

### Scanners – Symbol Barcode Scanner DS4308

Scan the barcodes below, one at a time, from 1 to 14.

| 1 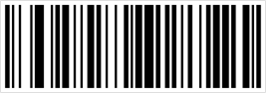 | 2 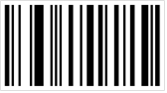 |
| --- | --- |
| 3 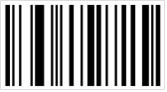 | 4 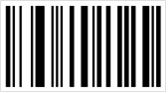 |
| 5 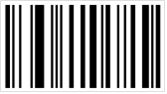 | 6 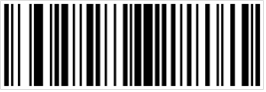 |
| 7 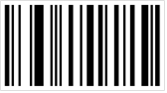 | 8 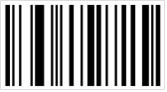 |
| 9 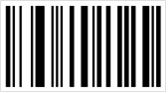 | 10 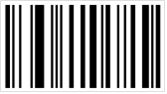 |
| 11 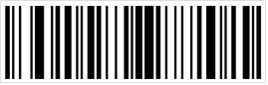 | 12 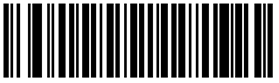 |
| 13 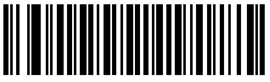 | 14 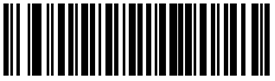 |

NOTE: Be sure that the scanner beeps (may be a set of differently pitched beeps) with every scanning step.

To RESET the barcode scanner to be compatible with Cerner CoPath (i.e., factory default settings), scan the barcode below:

| 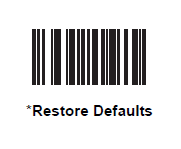 |
| --- |

NOTE: Be sure that the scanner beeps after scanning. The scanner should undergo a reset routine, with your PC detecting the device as a newly connected device.

Scan the barcode to lower the “beep” volume

| 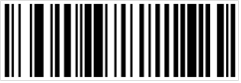 |
| --- |

### Scanners – Zebra DS46 Barcode Scanner

| To Configure Scanner for Epic | To Reset for CoPath (factory setting) |
| --- | --- |
| 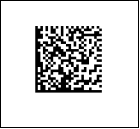 | 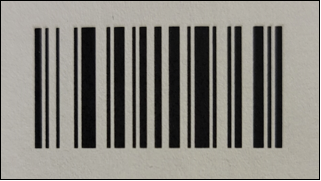 |

### Finding Patients (inc. Chart Review)

| 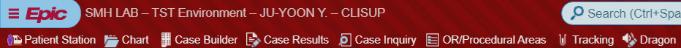 | Patients can be found through different routes, including Patient Station, Chart Review, etc.--best routes depends on the task at hand |
| --- | --- |
| 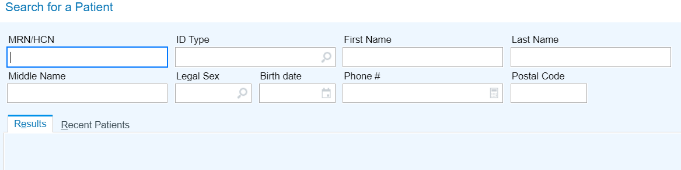 | Patients can be searched using different parameters—of note:   - SMH, SJHC, and Providence charts have been merged into unified charts - Patients may have 4+ IDs, including the various legacy IDs, including SMH (7-digit numbers), SJHC (J+8 digit numbers) & Providence (8-digit numbers) - All patients are assigned new Epic IDs (7-digit numbers) - These can be specified under “ID Type” - SMH legacy, SJHC legacy, PROV legacy |
| 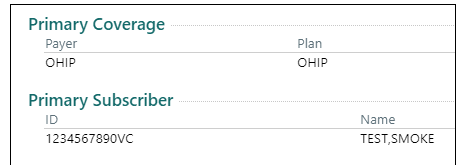 | OHIP information can be found under coverage |

### Ordering (inc. Marking STAT orders)

Ordering for clinicians are performed as below. Note that different pathways exist in the Unity Health’s instances of Epic, with functionalities that do not overlap completely. Please keep in mind that part of the functionalities is driven by the Source & Type list, established for the entire institution.

| 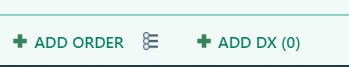 | From “Patient Station” > Choose relevant encounter (admission) > Choose “Add Order”   - Also possible from Chart Review > Encounters |
| --- | --- |
| 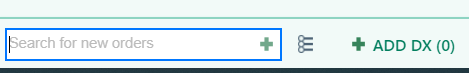 | Search for appropriate order:   - “Tissue Exam” - “Non-Gynecologic Cytology” - “Fine Needle Aspiration Cytology” - “Pap/Gyn Test” |
| 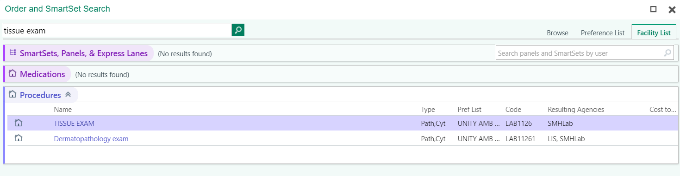 | *OR* click on the “+” button to pull up orders |
| 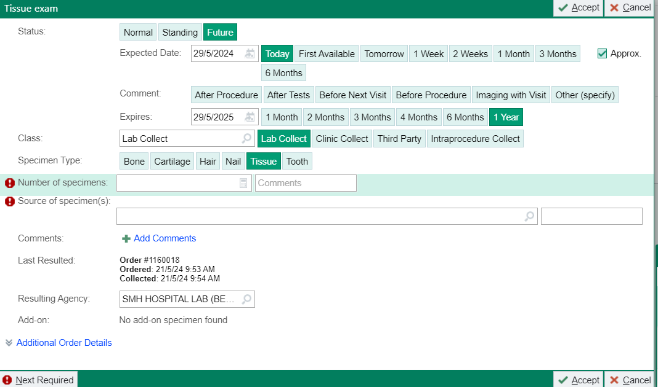 | Fill in the order form:   - Number of specimens - Source of specimens - Specimen types are limited – bone, cartilage, hair, nail, tissue, tooth; tissue chosen as default |
| 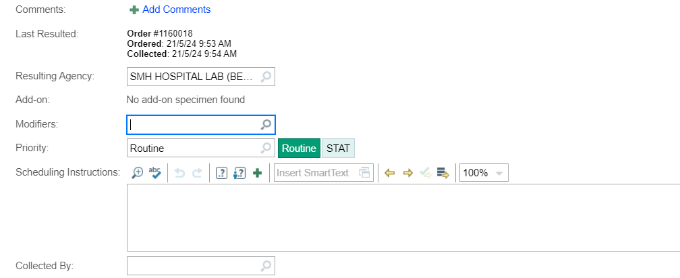 | To mark order as “STAT”, click on “Additional Order Details” > Change “Priority” status from “Routine” to “STAT”   - Orders NOT marked as STAT from the initial order status CANNOT be changed to STAT later in the workflow – this is feasible in ClinPath tests only |
| 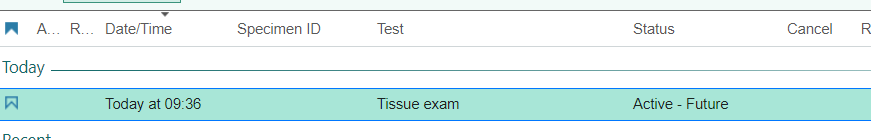 | Sign the order – the order appears in Chart Review as a new pathology case, to be collected |
| 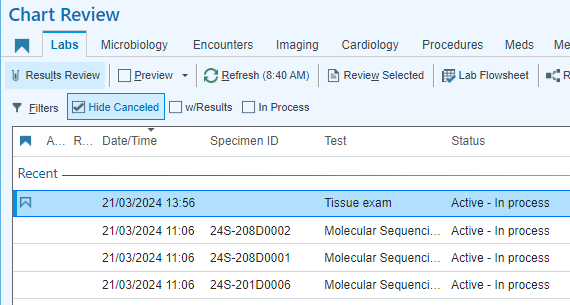 | Orders will now appear in patient’s chart as “Active - In process” |

- Specimens specifically marked as a priority (urgent, critical, or STAT) shall be handled accordingly. There shall be a documented procedure for the receipt, labelling, processing and reporting of these specimens. (V.C.1.1, REF 199)

### Orders – Linked Orders

| 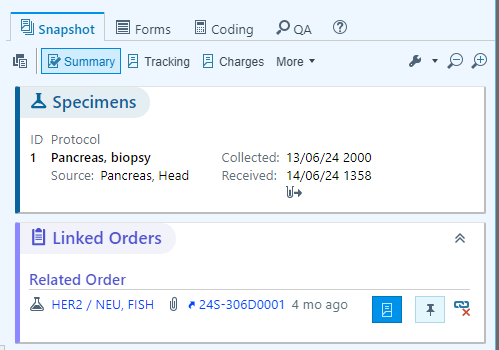 | Different lab orders may be “linked” in the Beaker system – in general, different pathology links are in “default” setting of the foundation system – linked orders are visible in the “Snapshot” panel of the resulting pane |
| --- | --- |

- Present linking algorithm is (as of go-live) related to ordering (ordered on the same day), and (specimen) container sharing
- Algorithm may be modified in the future

### Specimen Collection

| 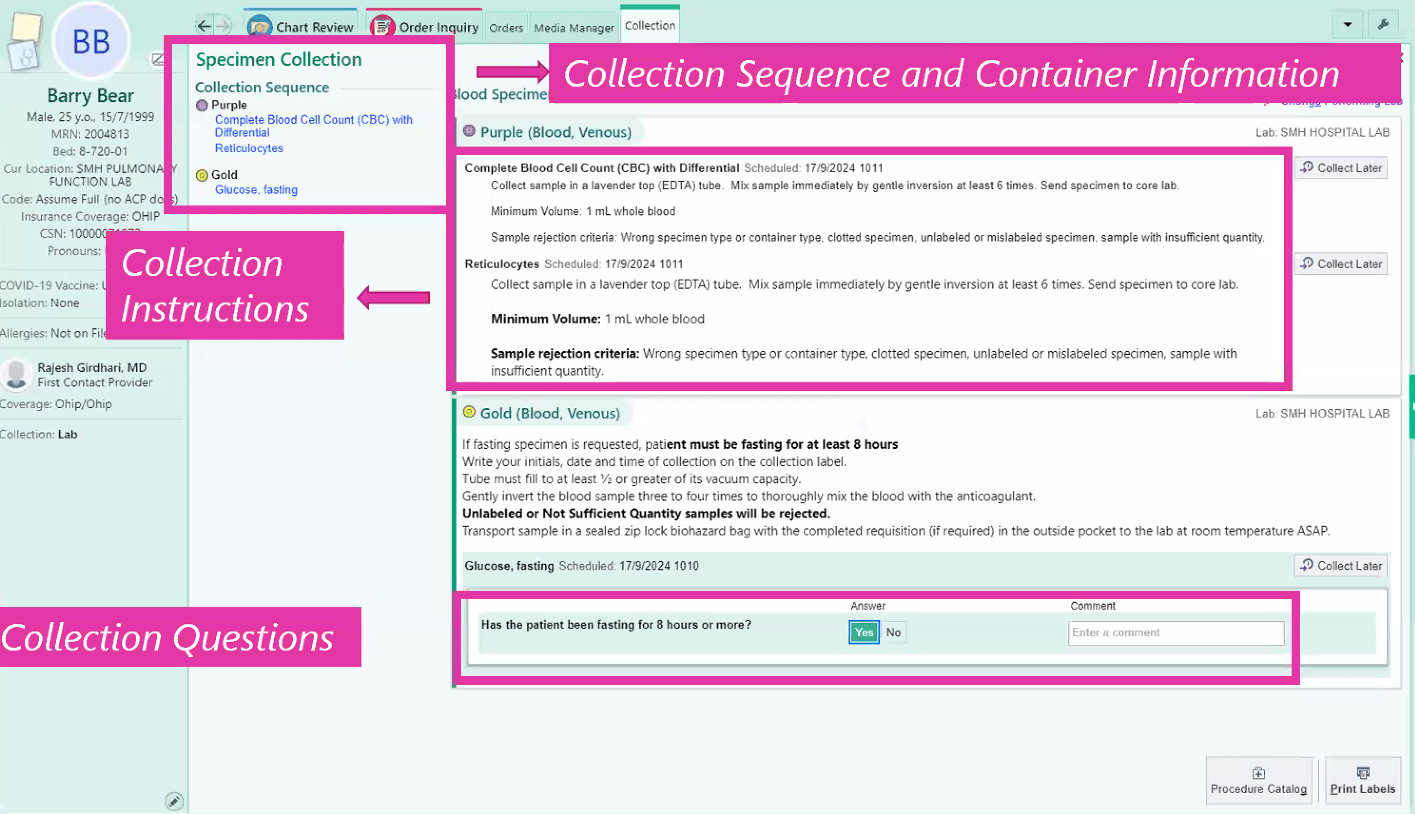 |
| --- |

NOTE: Specimen collection, including that in ORs, should be scanned, tracked events. That time should match the start of ischemic time for pathology cases. However, this aspect of workflow is unvalidated at the time of go-live, as testing this was not available to the lab medicine team.

### Specimen Collection – Infectious specimen documentation/handling

| 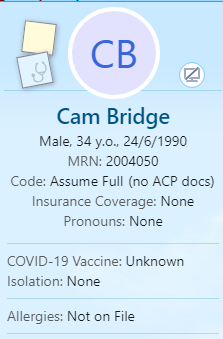 | “Patient Isolation Status” and “Patient Infection Status” - found in patient chart:   - Click “Isolation” - choose current encounter - “PUI” label appears   2 current alerts are more easily viewable:   - PUI flag (person under investigation) - PUI flag also appears on specimen container label - Epic Storyboard (see below) |
| --- | --- |
| 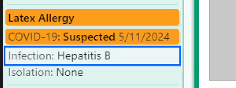 |  |
| 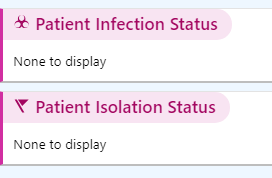 | Infections status & Isolation status are shown  PUI status on label is based on “Infection Status” |
| 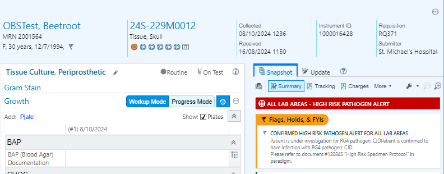  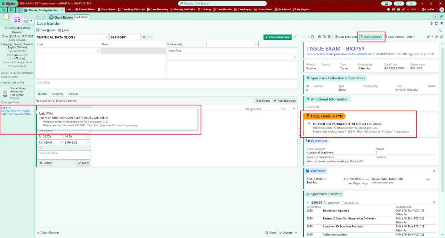 | A separate flag can be placed (controlled by microbiology) for CJD *only*   - This does not appear on specimen container label |

- There shall be a process for managing patients/clients with symptoms of communicable disease(s) to prevent transmission to others in outpatient specimen collection. (V.A.4.1, REF 1571)
- There shall be instructions for the following collection activities:
  (a) instructions for verification of the identity of the patient;
  (b) instructions regarding containers and additives;
  (c) instructions for the amount of specimen to be collected;
  (d) instructions for the proper mixing of tubes;
  (e) instructions for specimen labelling;
  (f) instructions for recording collector identity, collection date, and, when relevant, collection time;
  (g) instructions for disposal of materials used in the collection; and
  (h) instructions for preventing and handling adverse events related to collection (e.g., fainting, nerve puncture or any other threats to patient safety). (V.A.1.3, REF 174)
- Each specimen shall be labelled at the time and point of collection and in the presence of the patient. A firmly attached label shall contain:
  (a) the patient's first and last name (or unique code number in the case of anonymous testing);
  (b) one other unique identifier (e.g., the admission/identification or accession number);
  (c) the date of collection;
  (d) the time of collection (for time-sensitive examinations only).
  When electronically generated, machine-readable labels are not in use, the identity of the person who collected the specimen, as well as the above information, must be written on each specimen at the time of collection. For microsamples where the specimen tube is too small for the above information, an appropriate labelling system may be defined by the laboratory. (V.A.14, REF 207)

### Specimen Collection – Scheduled Collection (ROSE, EBUS, Bone Marrow), using Snapboard

| 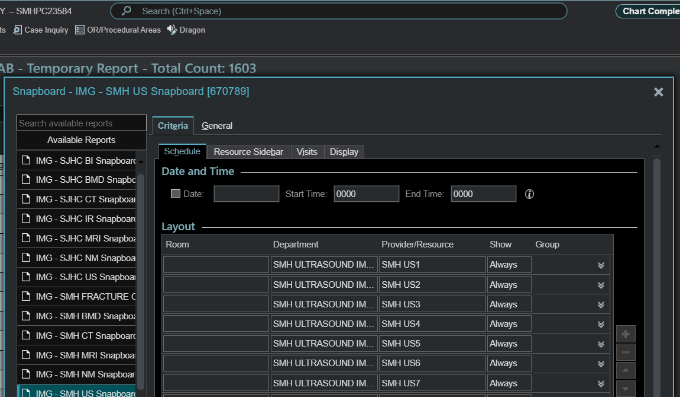 | Open Snapboard (Search > “Snapboard”)   - Choose the appropriate snapboard to explore, and click “Run” |
| --- | --- |
| 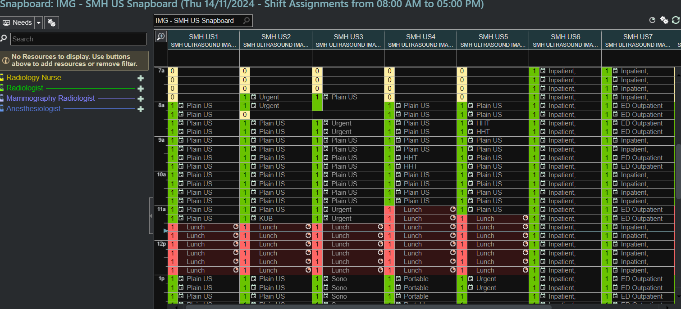 | Snapboard design is requested to highlight the procedures requiring pathology assistance. However, this function is unvalidated as of go-live. |

The Snapboard function has been setup, as of go-live date, for various imaging stations, ORs, and tumour boards.

### Specimen Receiving – Rejecting Specimens

| 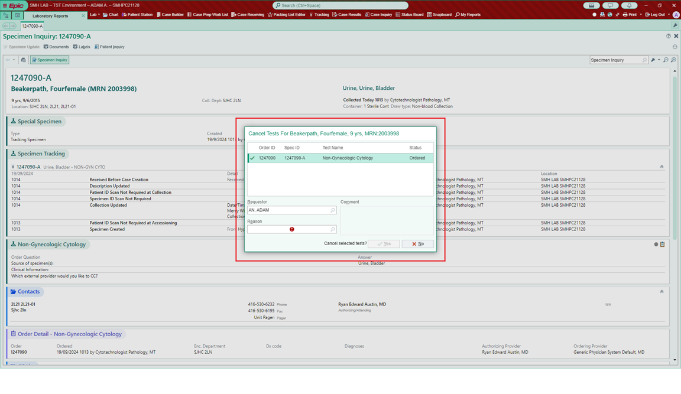 | When specimen has not been accessioned yet – go to “Specimen Inquiry”   - Scan (Specimen) Container Label - Select Reason for cancellation - Select Yes   NOTE: Refer to prior SOPs for rejection criteria.   - Source and Type are found on the Container Label   NOTE: Be sure to fill out the specimen rejection form (as per standing, legacy SOP) |
| --- | --- |
| 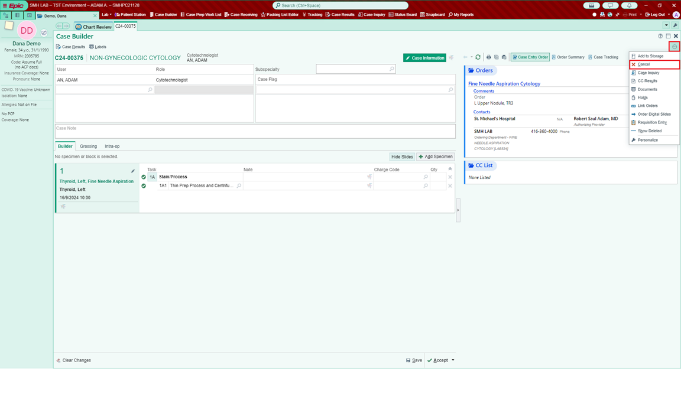 | Specimen Rejection – when case has been accessioned (Case Builder)  Go to Action Button and select Cancel  ***Case must be saved before it can be cancelled with this method |
| 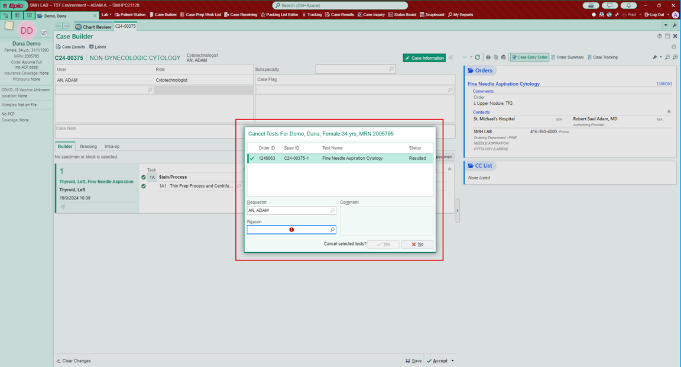 | Specimen Rejection – when case has been accessioned (Case Builder)  Select a Reason for cancellation, document any notes, then click Yes |

### Specimen Transport – inc. Send-out testing & Packing list editor

| 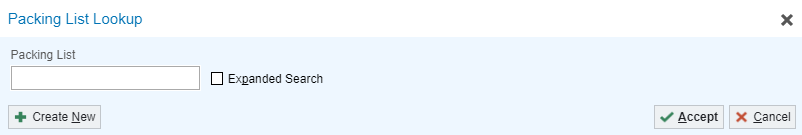 | Epic > Lab > Packing List Editor > Create New   - Unity Anatomic Pathology Packing List - List ID generated automatically |
| --- | --- |
| 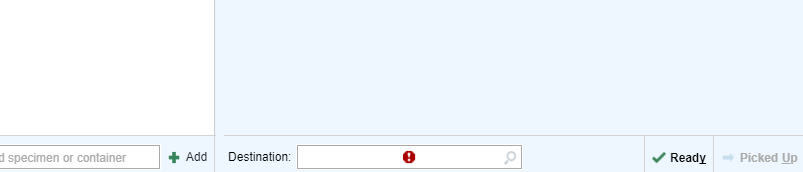 | Choose appropriate destination |
| 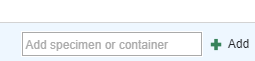 | Enter case information |
| 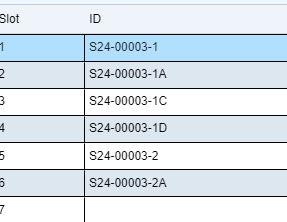 | Case parts are all entered   - Note the “Tests” and “Priority” columns are presently unavailable for editing |
| 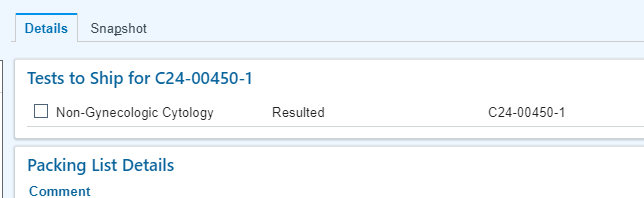 | For transport between sites, “Tests” may also need to be shipped.   - For *Cytology* tests, cases being sent over need to be with the materials to be signed out. i.e., Click the tests and “send over” the tests. - For *Surgical Pathology* tests, “tests” need *not* be sent over, unless the case is also be grossed at the other site. |
| 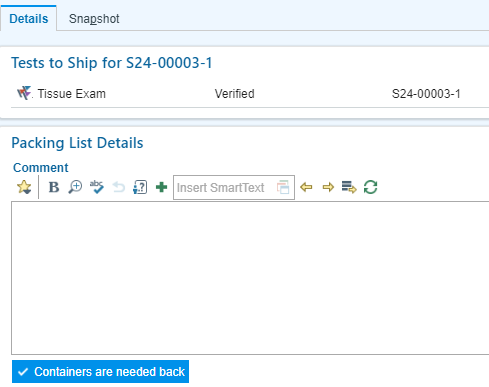 | Click the slot of interest, and designated the desired send-out test in “Comment” text box   - Click “Ready” when ready for pick-up by courier – the packing list is automatically printed - Click “Picked Up” once pickup has been completed - Look in case inquiry to track information |
| 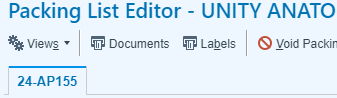 | Clicking on “Ready”, will automatically result in the shipping manifest being printed. The manifest can also be manually printed by clicking on “Documents”.  Click “Picked Up” when the shipment has been picked up. |
| 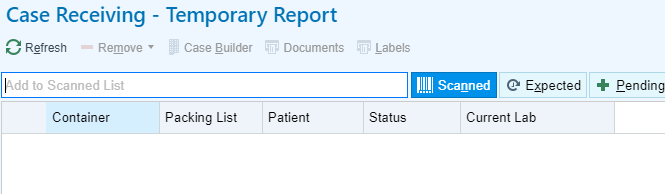 | On receiving the end, go to Case Receiving, and scan the manifest. The entire shipment is marked as having been received at SMH/SJHC. |

### Specimen Transport – Send-out Tests

| 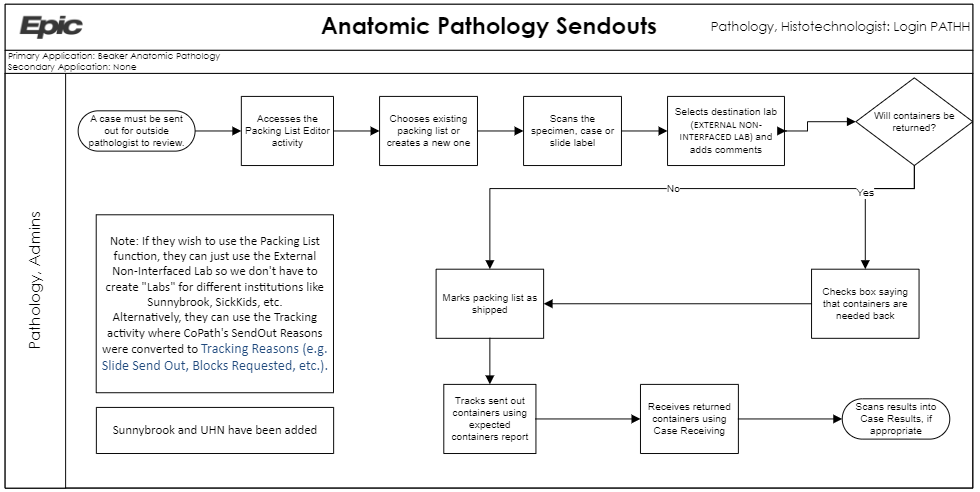 |
| --- |

| 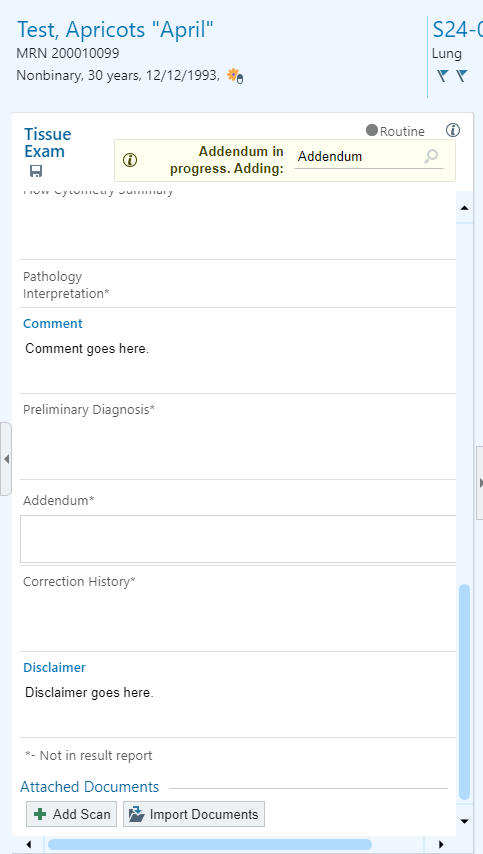 | Notify the path admin persons for cases needing to be sent-out.   - Pathologists and path admin fill out the appropriate outside requisition for the required test(s)   Create Addendum to the case, and click “Add Scan” to scan in a copy of the requisition to the case  NOTE: “Add Scan” function is not available to cases signed out, unless Addendum or Amendment is enabled. |
| --- | --- |
| 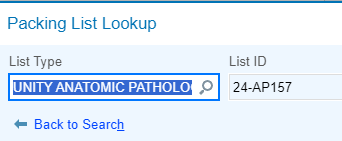 | Create a new Packing List, and choose “Unity AP Packing List” |
| 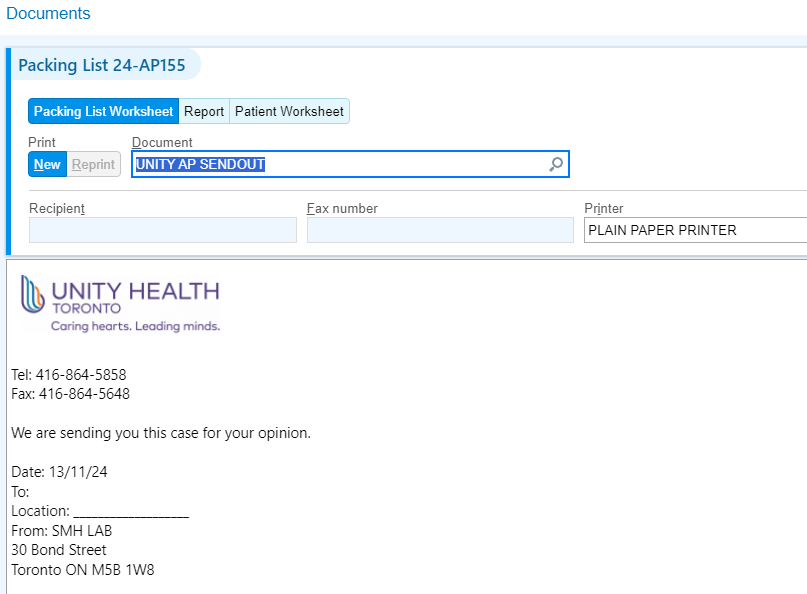 | In Packing list, choose Documents, and choose "UNITY AP SENDOUT” under Document. A default, generic cover letter is generated.  In the cover letter, attach copies of the outside requisition forms, previously filled out (see above).  NOTE: There is no workflow to generate an automated cover letter to reflect outside tests being requested. |
| 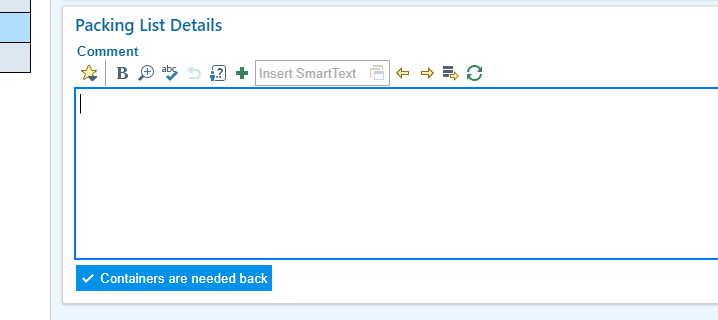 | The “Comment” section of the Packing list Details is for internal purposes only – the information will not be conveyed to the other institution. |
| 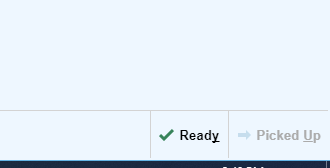 |  |

### Accessioning (& Case Receiving)

| 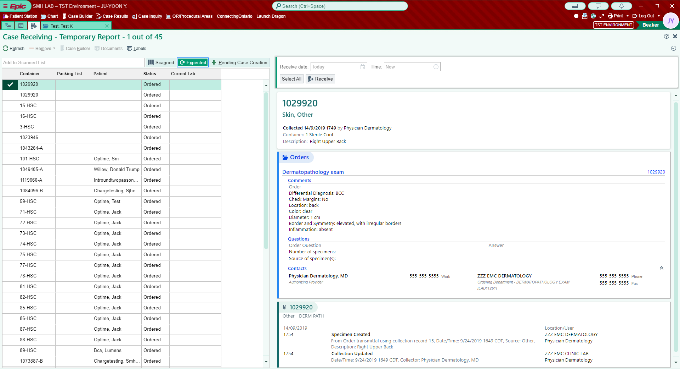 | Open Receiving list   - Alternatively, from Case Builder, click “Case Results” |
| --- | --- |
| 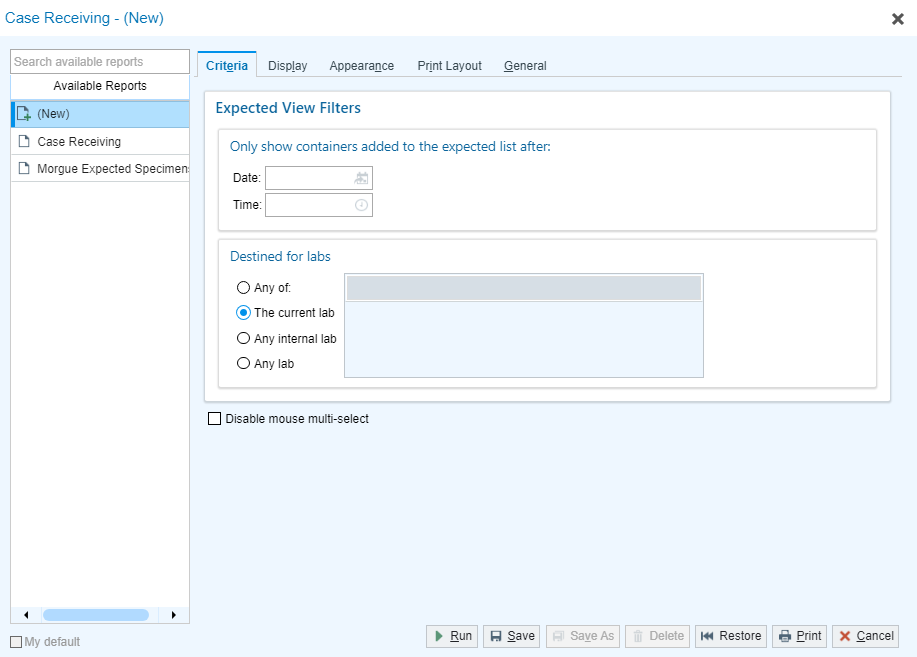 | Access “Case Receiving” by clicking on Epic > Lab > Case Receiving   - Pop-up window allows you to choose/setup desired setup - Click “Run” to run report |
| 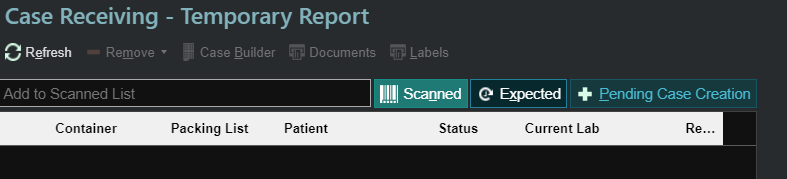 | "Scanned” cases |
| 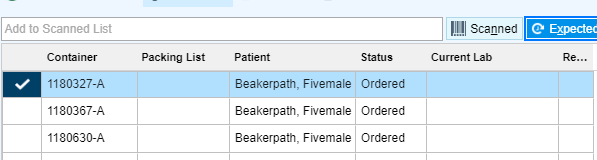 | “Expected” cases can be viewed |
| 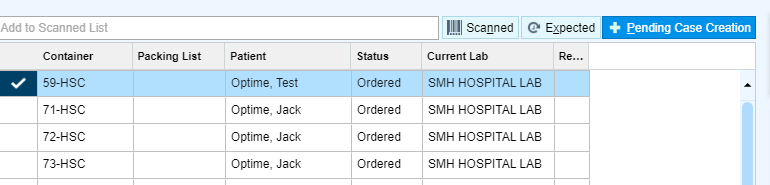 | “+ Pending Case Creation” allows you to view orders in system, where cases have not yet been built in Beaker |
| 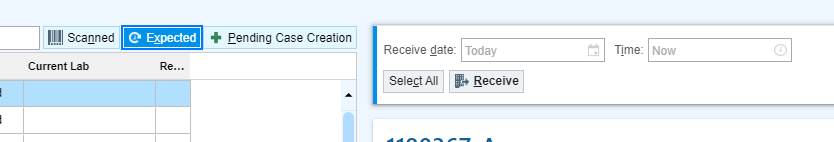 | Scan the container or click “Receive” to receive the container |
| 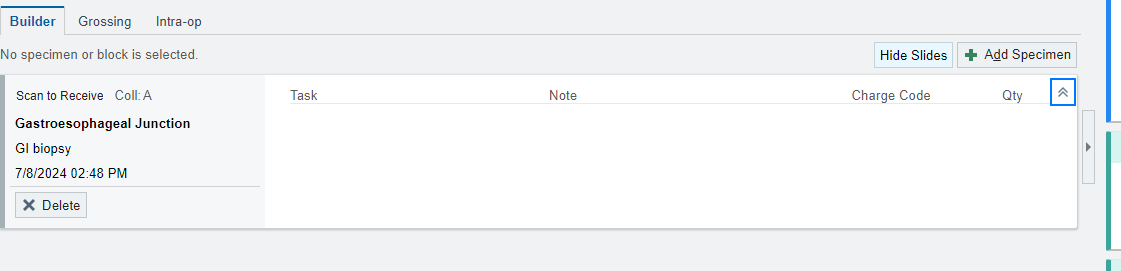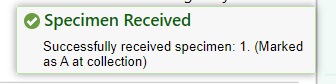 | The specimen can be received in case builder as well.   - Click on ‘case builder’. - Scan the specimen container label. - This will create an S# for that specimen and will mark the specimen as received. |

### Accessioning – Lab Requisition Entry

| 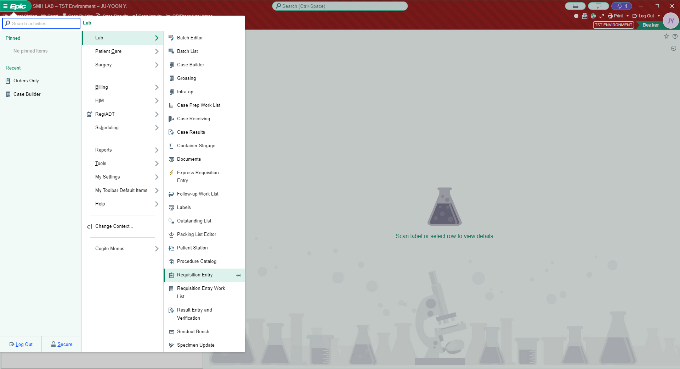 | Epic > Lab > Requisition Entry |
| --- | --- |
| 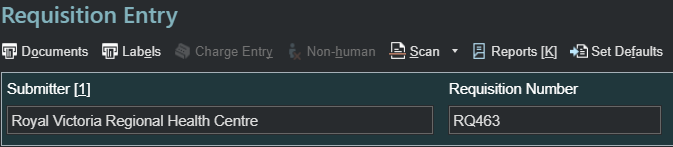 | Enter Submitter   - Requisition number is automatically assigned |
| 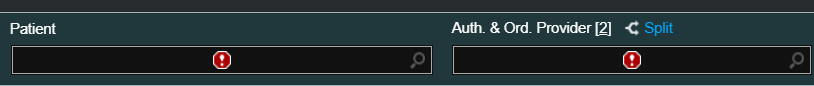 | Patient, Authorizing & Ordering Provider are entered |
| 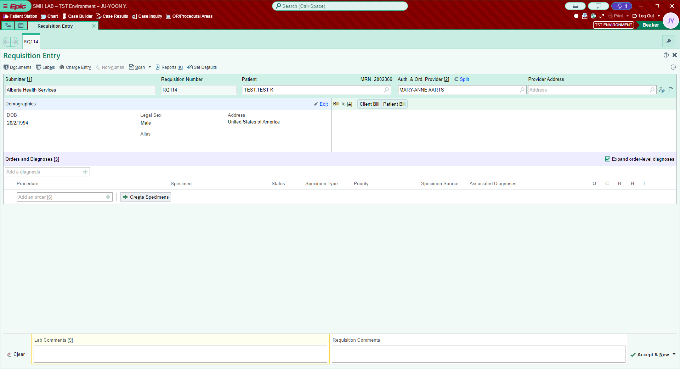 | Cases will appear as “Active - Needs to be Collected” |
| 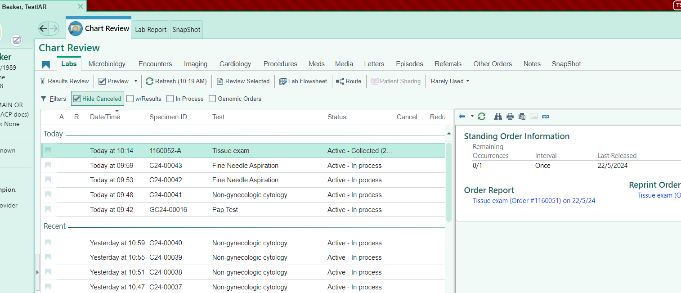 | Orders will now appear in patient’s chart as “Active - In process” |

### Accessioning – Consultation (“referred-in”) cases

| 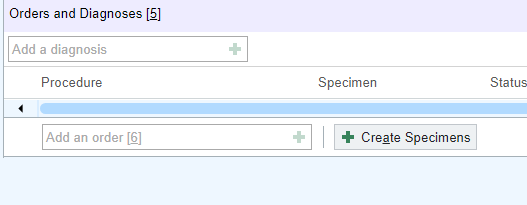 | New referred-in case is created in Epic > Lab > Requisition Entry   - Input “Submitter”, Patient identifiers - Requisition number is automatically generated - Add order “tissue exam” and click “Create Specimens” |
| --- | --- |
| 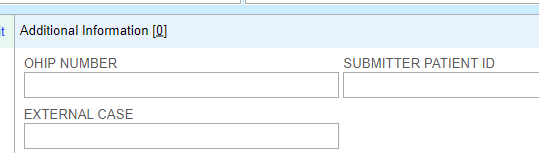 | Be sure to enter OHIP Number, including version code.  Enter “External Case ID”, and submitter patient ID (outside hospital MRN). |
| 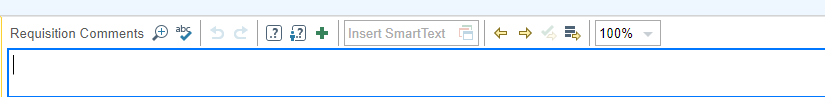 | Under “Requisition Comments”, enter clinical history, or any additional, relevant clinical information provided by the referring institution |
| 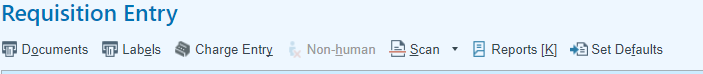 | Scan in the consultation letter, by scanning on “Scan” |
| 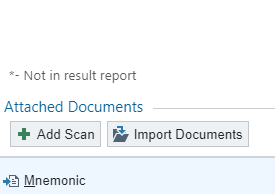 | Scanning is also available from “Case Results” - In Case Results, scroll to the bottom of the case, and click “Add Scan” to scan in the outside requisition. |
| 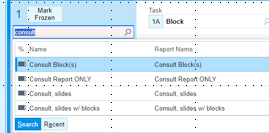 | Enter Case Builder.   - For each slide/block to be entered, search under “Consult” and choose the appropriate option - Histology and any task protocols triggered will generate Unity-specific labels |
| 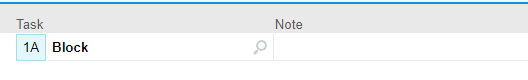 | Under “Note” for each block/slide, enter the corresponding outside block/slide IDs. |
| 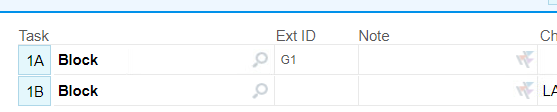 | From Case Builder, for the different blocks, enter a flag (click on the set of flags under “Notes”) |
| 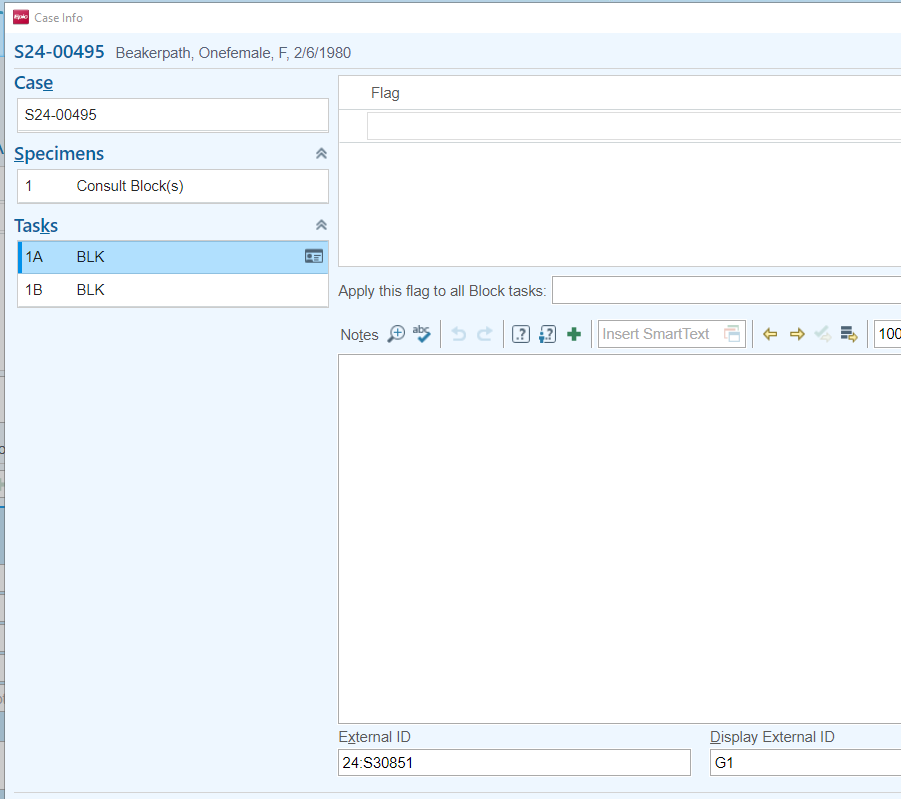 | In “Case Info”, enter the External ID:   - External ID = outside case ID - Display External ID = outside case block ID   The external ID will appear in the slide labels |
| 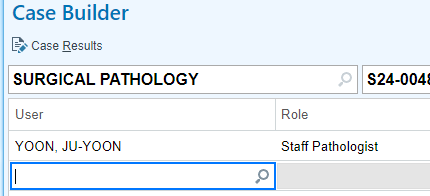 | Assign the case to the appropriate consultant.   - The case should now |
| 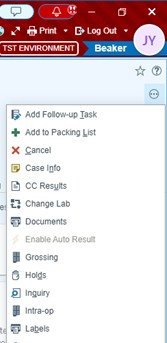 | Click on the “Action” button (3 dots), and click “Labels” |
| 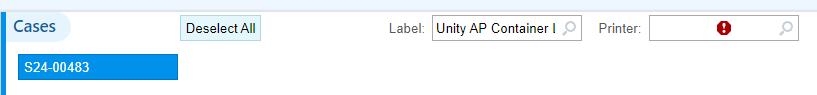 | Print a Container label by clicking on the Case ID, choosing “Unity AP Container label” and designate the appropriate printer (ZT231 model).   - Place the sticker on the outside requisition letter. - Place labels on outside blocks.   place the case in the consultant’s box. |
| 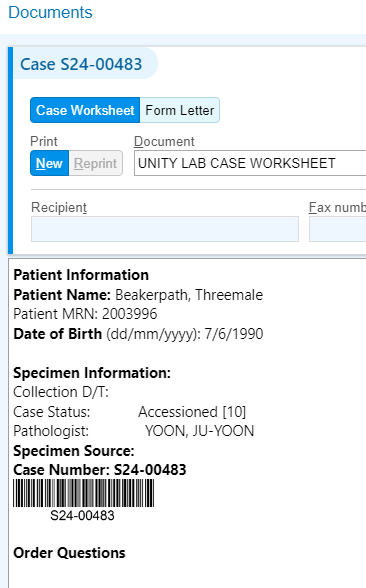 | Click on the “Action” > “Documents” and choose “Case Worksheet”. Print a copy, and place the sheet, and materials (slides if available) into the consultant’s mailbox. |
| 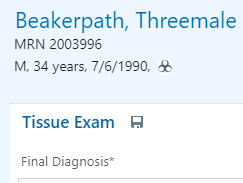 | When finished, click on the “Save” icon in Case Results and close the case. |

### Accessioning – Historical case workflow

| 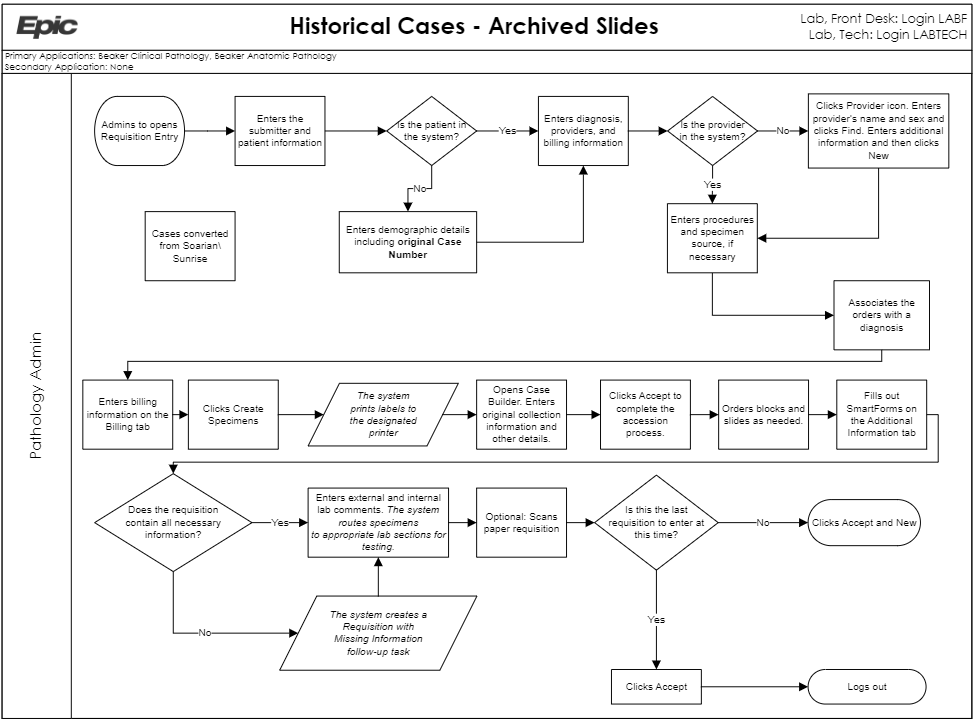 |
| --- |

| 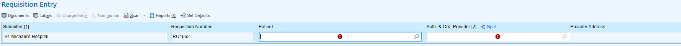 | Cases are added in Requisition Entry. “St. Michael’s Hospital (141)” is chosen as the “Submitter”.   - SJHC is NOT in the list as a separate submitting institution - Requisition number is automatically assigned |
| --- | --- |
| 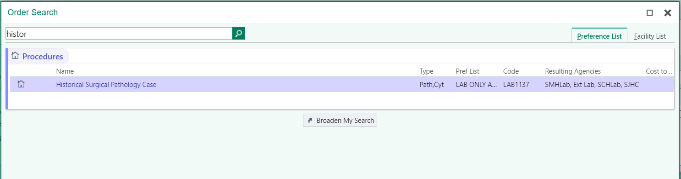 | Under “Procedure”, create order for “historical surgical pathology case” and click on “Create Order”.   - Can search for the order by typing it in the search bar. |
| 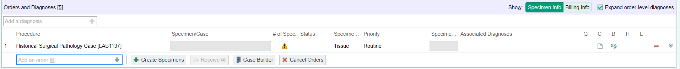 | Enter number of specimens  Enter any comments in the ‘lab comments’ field.  Click on case builder. This creates an H# that can be used to track the case. |
|  | Assign the historical case to the appropriate pathologist.   - Any case flags can be entered in the case flag dropdown menu. |
|  | Description of the specimen can be added in the description field.  Specimen note can include a description of the materials relevant to the case, for example the block information and any slides.  Click on add tasks to add a block. |
|  | Click on block 1, this adds one block. to add multiple blocks, edit the quantity field on the right.  When the block is added click on flags icon in the notes area. |
|  | Flags can be attached to the block here.  In the external id portion, the full block number can be entered. (ex. S24-12345-A1)  In the display external id portion, an 8 digit number representative of the full block number can be entered. This number will show up on the slide label (ex. 12345-A1) |
|  | Any tasks such as special stains and IHC stains can be added to the block.   - click on the block. Click add tasks - Select the desired stains. - For H&E stains to be ordered, use the .same level H&E or .deeper tasks   These tasks will show up on the cutdowns CPWL. |

### Accessioning - Container sharing

|  |
| --- |

While “Container sharing” was raised a possible workflow between lab divisions, this function was not properly explored, and the function did not undergo validation. Container sharing differs from “linked orders”, in terms of generating a potentially single report for multiple tests. However, it should be noted that there are several workflows where AP and CP (clinical pathology) tests interface within the Beaker system, including flow cytometry, and molecular pathology assays. These interfaces generally have been built to rely “triggers” ordered in the AP system, which results in a CP test being ordered. The specimens received from AP, which may range from fresh tissue to unstained slides, are handled in the Beaker system as new specimens.

### Finding Cases

|  | Pending cases are generally found in some views of Outstanding List, which is setup as the default landing page for many AP staff members.  Lists can be sorted   - Right click on column heading to filter as desired - Enter your last name in the Search box to filter by MD’s name to filter for assigned cases - Click on case to enter Case Results - Scan slide/container labels – within Beaker activities (e.g., AP outstanding list) - “Recent” cases - signed out/advanced in last 24 hours |
| --- | --- |
|  | Cases can also be searched in Case Results |
|  | From outstanding list - right click to:   - Assign cases, etc. - Enter “.me” to enter yourself - Residents can also be assigned |
|  | Click on “... (Action)” button to access chart review, etc. |

| Snapshot   - Linked Orders = orders are linked based on set of rules – same day orders, similar body site, etc. - Orders can be linked manually - Linked Order reports are sent out externally (e.g., faxed) together   - Forms – synoptic forms can be entered | |
| --- | --- |
|  | Activity tool bar for Outstanding worklist   - Pin – useful for filtering for pinned cases - List can be searched, filtered, sorted - Left click on column header - Right click on column header |
|  | Fr = case has frozen specimens  T = status for tasks   - Currently does not update follow-up tasks   S = send-out status  M = ex. REQ, IP  R = resulted, signed out, etc.  L = linked orders  Linked IDs = IDs for the linked orders |
|  | Assigning cases:   - Take = take unassigned cases - Put Back = return assigned cases - Assign = assign case to a pathologist   Use “.me” to choose self |

### Case Builder

|  | Case Builder can be accessed from:   - top screen (see left) |
| --- | --- |
|  | Can also be accessed from Pending List > Views >   - Note: pulling cases directly from the Case Builder button requires leading 0s to be present when entering case – c.f., year does not; e.g., S24-00098 can be searched as S00098, but not S24-98 |
|  | Case Builder used to:   - Add Specimen - Add Block - Specify Protocols - Within Case Builder, scroll down to “Attached Documents” and click “Add Scan”   NOTE: Typing parts of protocol number will pull up *recent* protocols, but is not a comprehensive search (click on magnifying glass) |
|  | If not visible, click on the double arrow icon to show blocks/slides in the system for the case |
|  | Choose Specimen Part > Choose “Edit Specimen” (pencil icon) |
|  |  |
|  |  |

### Case Builder – Editing Case Information

|  | Case Builder screen for a given case > “Case Information” button |
| --- | --- |
|  | Case Inquiry |
|  |  |
|  |  |
|  |  |
|  |  |
|  |  |

### Case Builder – Marking as Frozen

|  | Choose Specimen of Interest and click “Edit Specimen”  NOTE: Case cannot be marked as frozen once the case has been signed out. |
| --- | --- |
|  | Clicking on “Mark Frozen” results in specimen being flagged, and generates a FS H&E slide order  NOTE: Specimen protocol needs to be entered before marking the specimen as frozen. Marking a specimen before adding the specimen protocol creates an FS Block which cannot be edited to add extra FS slides or smear slides.  Steps are as follows: add specimen protocol -> delete HE -> mark as frozen. After intra-op is done the initial HE is added. |
|  | To add more frozen section slides, click on the block and search for frozen. Select ‘frozen section, each additional’. To add a smear, click on the block -> search for smear -> select ‘SMH-smear/ touch preps’. If more smears/touch preps are needed select the ‘smear/touch preps- additional' |
|  | To print slide labels, click the labels icon -> select frozen section slides -> select ‘Unity AP Slide Labels’ from the label dropdown -> select the desired printer in the printer dropdown -> click print labels. |
|  | From Case Builder, click on “Intra-op” and enter QS diagnosis as appropriate. Click specimen done to complete intra-op diagnosis.  NOTE: QS diagnosis cannot be entered, once the case has been initially signed out. |
|  | Completing intra-op diagnosis confirms blocks and frozen section/smear slides- denoted by the green check mark beside the tasks.  Cassettes (and slide labels) can be printed at the time of grossing via the ‘labels’ icon. Select the cassettes that need to be printed -> select ‘Unity AP Cassette labels’ in the label drop down -> select the desired cassette engraver in the printer dropdown -> click print labels. |
|  | Time stamp – at time of accessioning/receipt of specimen & resulting, etc can be seen in case tracking via case inquiry. Click case inquiry -> enter case number -> scroll down to case tracking for the time stamps. |

### Case Builder – Assigning cases

|  | Click case on pending list & click “Assign” |
| --- | --- |
|  | Single cases can be assigned in case builder. Click case builder -> enter case number -> select a pathologist |
|  | Multiple cases can be assigned to one pathologist in the outstanding list via the ‘AP Unassigned Cases’ view. Click on all the cases to be assigned to a pathologist (these can be searched as well via the search bar) -> click the assign icon -> select assign -> select the pathologist.  A list of cases can be created by scanning one slide from each case. Also, a case can be added to this list by typing in the case number in the search bar (ex. S### (S298) or S24-##### (S24-00298)). Highlight each case by clicking on each case or by holding the shift key and clicking the first case of the list and then click the last case of the list, all while still holding the shift key. Click the assign icon, select assign, and select the pathologist. |

### General – QA/QC/QM

|  | In general, various QA/QC/QM activities will continue using paper forms and other manual processes (to be re-visited during Epic Beaker Optimization)  Select QA/QC/QM activities are available for pathologists - From Case Results – enter “QA”   - Choose appropriate QA event as appropriate |
| --- | --- |
|  | Findings are entered as appropriate – example:   - Findings – main results as appropriate - Level - e.g., qualifies the findings above - Comment – additional information, if required |

### General – Add Follow-up Task

|  | From Case List, right click & choose “Add Follow-up Task” |
| --- | --- |
|  |  |
|  | Select the task to be done.  Enter a start date for the task, in the date field, enter T for today's date. In the time field, enter N for the time right now.  A due date for the task can be entered as well.  Assign a user to complete the task in the assigned user dropdown menu.  In the notes section, enter any details about the task. For example, the block number/ slide number to be pulled. |
|  | Follow-up tasks are tracked in "Follow-up Work List” (Epic > Lab > Follow-up Work List)  The views filter the list by the task or by the tasks that you’ve been assigned to complete (‘My Follow Up’)  Any events leading up to the completion of the task can be added to the ‘Event Details’ field. Any comments entered here are time stamped. |
|  | To mark the task as complete, click on the complete button on the bottom right corner of the page.  Enter a comment and click accept to finish the task. |
|  | All completed tasks are placed in the ‘Closed Task’ View.  To search for a specific case in the list, click on any case in the list, and press ctrl+F. Type in the case number. |
|  | Any completed task can be ‘re-opened’ again by clicking the re-open button on the bottom right corner of the page.  This resets the task to incomplete and will show back up on the assigned user’s ‘My Follow Ups’ view. |

- Currently no Follow-up Work List is specifically designed for PAs for re-grossing, adding sections, etc. (for optimization)
- List “Cases Requiring Second Pathologist Review” is available for pathologists

### General – Flagging cases, specimens

|  | Flags can be entered at case, specimen, task (slide level), or block level. Case flags are available in Case Builder (flag button) |
| --- | --- |
|  | Choose the appropriate flag from the drop-down menu, and enter text as appropriate.   - Flags can be entered for any of the three levels as appropriate by clicking on the buttons on the left - Click “Accept” to save and exit |

### General – Downtime Procedures

|  | BCA = business continuity access   - Use devices connected to red plugs |
| --- | --- |
|  | Use legacy requisition form:  <https://unitynet.unity.local/wp-content/uploads/2022/05/74625-surgical-pathology-requisition.pdf> |
|  | Documentation of downtimes will be performed by the IT team using their whiteboard process, including “code grey” procedure  Cases will be logged manually using paper forms – cases will be entered retrospectively using Requisition Entry |

### General – Smart texts and smart phrases

|  | Enter “SmartPhrase Manager” and click “New User SmartPhrase”  Alternatively, search for “SmartPhrase” - choose SmartTool Editors   - Choose SmartPhrase to be edited - Can also “Go To My Phrase” to edit user’s own phrases |
| --- | --- |
|  | Also, Smart phrases can be created by:  1) Type desired text  2) Click on the Green Cross button  3) Ensure no PHI is included and click on “Continue” |
|  | Editing SmartPhrases:   - Search for “SmartPhrase” - Ensure no PHI is included - Rich Text *vs.* Plain Text – formatting is retained in “rich” text |
|  | To input the smart phrases, when typing, enter “.xxxx” to enter smart phrases   - xxxx = name of the smartphrase - Press F2 to move to next field – or say “next field” when using Dragon - Smart links can be used – e.g., “.age” for patient age (can be dictated in Dragon) |

### General – Useful SmartLinks

| .sig | Signature, with date & time stamp |
| --- | --- |
| .me | Enters your name |
| .today | Today’s date |
| .now | Time now |
| .casenumber | Case number |
| .mrn | MRN |

### General – Using Dragon

|  | Dragon Medical One is enabled and accessible from Epic Beaker |
| --- | --- |

List of available Dragon commands are available in “Speech Recognition Starter Set - Hyperdrive.xls”

### General – Billing & Charge Codes

In general, the validation and custom building exercise prior to go-live did not sufficiently address billing and workload tracking functionalities. Charge codes & associated quantity can be entered manually. Some workload codes are automatically populated. At the time of go-live, this function should be considered non-validated.

## GROSSING AND HISTOPATHOLOGY

### Grossing

|  | From Case Builder, click on “Grossing” tab   - Builder tab is the default tab that opens when a label is scanned in Case Builder |
| --- | --- |
|  | Alternatively, use Dragon to open the Grossing tab using voice commands.  Voice command: “Open gross”   - see Dragon documentation - Command List tab, Row 320 |
|  | Clicking on specimen part automatically inputs default text, including specimen name, and (protocol-associated?)  Smart phrases can be added |
|  | Blocks can be added from Task Protocols |
|  | “Copy Last” to add 1 block at end.  “Remove Last” to remove the last block.  “Print New” to print the “unconfirmed” (open square) cassettes  “Specimen Done” to mark specimen part as complete   - Grossing text can still be edited afterwards   “Gross Done” sets the status of the case as “Gross Done”. |
|  | Marking the case as “Specimen Done” with unconfirmed blocks results in following popup.  NOTE: After blocks have been deleted, the same block ID cannot be re-assigned |
|  | Click “Expand Specimen” to go back to specimen and add cassettes as needed |

### Grossing – Gross-only cases, inc. foreign body

|  | Certain specimen types will be designated at the time of ordering.   - Several types have been removed as part of specimen type harmonization between lab medicine divisions |
| --- | --- |

### Grossing - Adding/Removing Blocks

|  | Go case builder and choose the specimen part of interest > Add Task |
| --- | --- |
|  | Choose from one of the available protocols for adding blocks |
|  | Pressing the right-most “X” mark will delete the block   - In case with multiple blocks, choosing one of the middle blocks will result in re-number of the blocks as necessary (feature previously not available in the Foundation system) - Similarly, slide levels are re-numbered as middle slides are deleted   NOTE: Deleting a block cannot be undone. As well, the old block number cannot be replaced by future blocks. |
|  |  |

- Auditing trails

### Grossing – Confirming Blocks

|  | While the check box can be clicked to “Confirm” the block by scanning, this behaviour was not confirmed during validation. |
| --- | --- |
|  | In lieu of scanning, confirming blocks is performed by clicking on “Print New”. To use Dragon, “Alt + W” hotkey can be used. - this results in new and all unused blocks being printed (and expected to result in double printing). This issue has been flagged during validation.  Click “Specimen Done” when finished with a part of a case. |

### Grossing – (Re-)Grossing post sign-out

|  | The grossing field cannot be edited post sign-out – as such, the workaround is:   - Pathologist would create an addendum - Add (re-)grossing text as part of addendum report   To add block post-sign-out (final verify), access Case Builder for the case and add blocks as per usual process |
| --- | --- |

### Grossing – Adding Flags, including cold ischemic time

|  | Flags can be added in Case Builder > “Case Information (pencil icon)”   - Click on the magnifying icon for options or search for flag ID - Choose appropriate flag, such as “Cold ischemic time breast” - Click on “Add Comment” and enter value (e.g., “30 minutes”). |
| --- | --- |
|  | NOTE: Cold ischemic and fixation times are also added as part of Breast Biomarker Synoptic Report Form. |

### Histology – Case Prep Work List (microtomy)

|  | In the “Search” bar, search for case prep work list.  Accessing CPWL for the first time shows all the possible ‘views’. Each view is a filter that shows cases with the different blocks and tasks associated with that view. For example, the cutdowns view shows all the cases with blocks and the tasks/stains and molecular tests that the pathologist orders after reading the initial HE. Select a view that will be most frequently used and click my default icon (bottom left of the window). |
| --- | --- |
|  | A list of all the cases is shown on the left. |
|  | Pending tasks are listed after clicking on a case. |
|  | Click “Print Labels” - marks task as printed (Print icon will be beside the task) |

The CPWL workflow: blocks are scanned to cut -> slide labels are printed -> slides are scanned to confirm the task (i.e. mark the task as completed.) Deviating from this workflow will give a notice to scan the block first.

### Histology – Tracking

|  | Tracking is available through Epic > Tools > Lab Tools > Tracking |
| --- | --- |
|  | New tab will appear |
|  |  |
|  | Tracking reports can be modified as shown |
|  | Slides failing QC will be tracked by going into Tracking > choose “Stained - QC Failed”.   - Scan the failed slide. - QC works will continue to be documented using the legacy paper forms. |
|  | Delivering slides:   1. SMH recipients: go into Tracking > Choose “Deliver” & scan each, individual slide to be delivered. Slides are delivered to mailboxes. 2. SJHC recipients: create a packing list > cases are received at SJHC *then* Delivered.   Slides to be filed are also tracked by choosing “Slide Storage – In” |
|  | Embedding:   1. Click the ‘Embedded’ tracking event in the toolbar. 2. Scan block to be embedded. 3. If block is not scanning, click the ‘look-up’ icon and enter the block number. |

- can add multiple cases to one pathologist in Outstanding List
- Unstained slides - do they have to be delivered? in Co-path they're not mandatory to be delivered in case assembly, they just get stored.
- scanning slides for delivery doesn't happen automatically upon scanning slide it happens for all other tracking purposes (updated 3/10 ABS)

### EM (electron microscopy)

|  | To mark a specimen for EM, enter the specimen protocol *EM Specimen.* |
| --- | --- |
|  | This will populate the EM tasks. This can be updated by clicking on the block and clicking on the ‘+’ sign in the add tasks field. |
|  | Search for the tasks and click on each task to add. Adjust the quantity. |
|  | Add the E#.   - Click on the flag icon beside any slide tasks. - Enter the E# in the display external ID field. - Take note of the slide number. This will be the slide that is to be printed to label the specimen. - Click accept. |
|  | Print the slide label with the E#.   - Click the labels icon. - In the slides section, click the slide label. - In the labels dropdown select *Unity AP Slide Label* - In the printer, dropdown select the label printer. |
|  | The tasks need to be confirmed in the Case Prep Worklist.   - In the Case Prep Worklist, under the ‘views’ section, click on settings (make sure *Scan Blocks is highlighted). |
|  | In the ‘task action’ dropdown, click on select all tasks then click run. |
|  | Click ‘Case Lookup’. Type in the case number with the block number.   - An override reason needs to be provided to move forward. - Switch the ‘print labels’ button to ‘confirm’. - Click the ‘All’ button to highlight all the tasks. - Select an override reason. - Click ‘confirm’ |
|  | Deliver the tasks:   - In case tracking, click on the delivered icon. - click the lookup icon to search for the case. - Every container (blocks and slides) for the case will appear. - Highlight all the containers for the EM portion- click on the first container, hold shift, and click on the last container. - Click track to deliver all the highlighted containers. |

### IF (Immunofluorescence) for Kidneys and Skin Specimens

|  | To mark a *skin* specimen for IF, enter the *SKIN Biopsy I.F. Panel.*  To mark a *kidney* specimen for IF, enter the appropriate specimen protocol:   - *Kidney NT. I.F.* - *Kidney TX. I.F.* |
| --- | --- |
|  | Print the labels.   - Go to Case Prep Worklist. Type in the case number with the block number via ‘case lookup’ |
|  | The slide labels for the skin/kidney IF portion should now be printed and need to be confirmed.   - Scan all the labels - Alternatively, change the ‘Print Labels’ button to ‘Confirm’. - Click the ‘All’ button to highlight all the tasks and click ‘Confirm’. |
|  | For Skin Specimens for IF, the pictures of the specimen need to be uploaded on EPIC. This is done in the outstanding list.   - Search for the case in the ‘search’ field. - In the bottom of the resulting field, click on ‘Import Documents’ - Find the pictures in the J Drive > Pathology > Specimen Images > *Year* ex. 2024 > Skin IF > *Patient’s Name.* - Select the pictures and ‘Open’. |
|  | The imported pictures are now available to view at the bottom of the resulting page.  There’s an option to have these images included in the final report or to keep it internal.   - Select ‘internal’ for all the images that have been imported to keep them off the final report. |

## REPORTS (inc. Sample report)

|  | Sample report header shown, includes (as of 7/Nov’24):   - Institution header & location - Patient information – name, DOB, sex, MRN, OHIP - Patient location - Authorizing (attending) & submitting (ordering) provider(s) - Patient information is available on each page of the report |
| --- | --- |
|  | Signature of sign-out pathologists are found below “Final Diagnosis” |
|  | Intra-operative data are displayed lower in the report. |
|  | Sample report footer (as of 7/Nov’24)   - Also shown are page numbers (not displayed in diagram) - Change requested to remove “CAP number” (pending) |
|  | Above results are not generated for cases not finalized, but can be viewed in chart review as preliminary results (if “Prelim Verify”d).   - Preliminary status is displayed below final diagnosis as shown |
|  | AP Lab Report templates (SMH version shown) can be modified in Lab Administration (access restricted) |

NOTE: The above view is generated reports through “Result Report (Case)”. Views can differ when accessed through chart review, etc.. The reports are currently generated only for final reports – preliminary results can be viewed in Chart Review, however, and

### Reports – Viewing charts, results & reports

|  | Click on “Chart” for chart viewing/review  Alternatively, “Patient Station” may be used |
| --- | --- |
|  | Enter patient info to search.   - “=” can be entered in the MRN search field used to look for the same patient |
|  | Click “Chart Review” and results are viewable in “Labs”.  Filter – most surgical pathology results should be found under “Tissue Exam” under “Order” |
|  | For viewing of reports, search “Result Report”:   - By Case – suitable for surgical pathology and cytopathology reports   - NOTE: may need to add leading “0”s (ex., S24-00001)   - Also valid are “S1”, “S2”, etc. - limits search to current year - By Specimen – for molecular cases   Result Report is suitable for generating hard copies of lab reports. |
|  | Entering the case/specimen ID will lead to "Create New Reports” screen, where you can choose the appropriate recipient and print reports |

### Reports – Result Review

|  | (as of 17/Oct’24) -  Microbiology is separate lab under Chart Review – for microbiologists  This tree is separate from filters in lab results  Separate from “Procedure category” - used for filtering results in Chart Review |
| --- | --- |
|  |  |
|  |  |

Result review represents a different way to review results (*vs.* Chart review), allowing more easily to trend results.

### Reports – Faxing Reports

|  | Add the desired recipient as "free text recipient" under "CC list"   - This will be relevant for cases where the outside provider could not be added as a recipient |
| --- | --- |
|  | Ensure the right recipient is now listed in "CC list" |
|  | Search for "Result Report (By Case)" and enter the case number |
|  | Choose the desired recipient, click "Add to List" |
|  | Check the fax number and ensure fax is selected as device - magnifying glass can be used to preview the report to be faxed |
|  | Click "Confirm" to fax the report |

### Reports – MyChart

|  | MyChart is Epic’s interface for patients to view results. |
| --- | --- |
|  | Lab results are viewed under “Test Results”   - The link "Scan1” links out to PDF version of the full report |

NOTE: This function could not be more fully tested by the lab staff members due to limited access to the platform. The MyChart app testing was performed by a separate team.

### Reports - Beaker Laboratory Report Routing

Routing preferences to internal and external providers will be defaulted based on your HRM (Hospital Resource Manager) subscription and employment status, and will determine where signed notes and results for Unity Health patients will be routed. HRM is a system that sends reports to doctors within the circle of care electronically, which avoids the use of faxes altogether.

The Epic Beaker laboratory information system will be routing reports based on the option chosen for different end users.

### Reports – Adding Recipients (Route)

|  | In Chart Review, select the case of interest > click “Route” |
| --- | --- |
|  | Add recipients as appropriate |
|  | Click Send – the test results will be available in “In Basket” message |
|  |  |
|  |  |

### Reports – OLIS Interface

|  | ONE Health testing platform |
| --- | --- |
|  | Original test of final report |
|  | Addended status appears as “corrected” |
|  | Addendum is added.  New synoptic report appears as “Amended”. |
|  | For participating institutions, referred-in cases/specimens will be created using Requisition Entry, and a MRN will be generated – OLIS results will migrate.   - For non-participating submitting institutions. |

### Reports – Connection Ontario

|  | Connection Ontario (OLIS) may be accessed from Chart Review view, by connecting “OLIS” |
| --- | --- |

Alternatively, the Connection Ontario app can be directly accessed from Unity Health workstation, or from Citrix.

## COMMUNICATION

### Communication – Critical Results & Comm log

|  | From Outstanding List:   1. Choose patient/case of interest 2. Click on “Comm Log” |
| --- | --- |
|  | Comm Log can be entered from Case Inquiry |
|  | Open Comm Log:  Results communicated with different contacts |

### Communication – In-basket message

|  | Newly available results are indicated as “in basket message” for clinicians.   - Click “Results” - Alternatively, click on the “Results (Flask button)” |
| --- | --- |
|  | Click on the result of interest – preview of the results is available in the active window. |
|  | In-basket messaging function can also be used to communicate between staff members about a particular patient/case. |
|  | Physician preference is setup in Report result routing record.   - e.g., “inpatient result routing” - SCR records exist for individual providers   List of providers desiring faxed reports were established |

NOTE: The above function could not be validated in the TST environment, as the SCR record setup could not be setup for validators. Algorithm for triggering automated in-basket message is unestablished as of go-live. Communication of critical results should use manual messaging, with/without e-mails. Note that selection of preferred notification method (e.g., faxing, in-basket message, etc.) was performed at the institution level, and different end users are setup accordingly.

### Communication – Secure Chat

|  | Access Secure Chat through the Chat Button |
| --- | --- |
|  |  |

NOTE: While Secure Chat was identified as a potential workflow for communication between staff members about a patient, this function was not further explored during the Epic Beaker validation.

## ANATOMIC PATHOLOGY (AP) Workflows

|  |
| --- |

### AP - Intraoperative Consultation (inc. Frozen Section)

In the absence of well-established procedures for communicating with the pathology group in large, clinical teams are instructed to call the lab to notify regarding frozen sections.

|  | Clinical teams requiring intra-op consultation are asked to call the lab (as with current workflow)  Prior to proceeding, look for the “PUI” designation on the specimen container label for potential infectious process risks ([see above](#_Specimen_collection,_including))  Scan the specimen container label to enter Case Builder and build the case  - Ensure that there is contact information posted with specimen  - Any infectious risks should be designated in the left panel of Case Builder, including “rule-out” flag (shown) |
| --- | --- |
|  | Scanning the specimen container label will date- and time-stamp the receiving of the specimen, viewable in Case Inquiry. |
|  | In Case Builder, mark as “Frozen”   - Marking cases as “Frozen” will automatically generate orders for 1 FS block and 1 FS H&E slides |
|  | Snow flake icon will appear, with case part being designated for Frozen |
|  | For gross-only cases, DELETE the automatically generated FS block and slide tasks. |
|  | Enter your information as the intraoperative consultant |
|  | Call the OR with the intra-operative results. Document the communication in the intra-op section. Use “.todaydate” to enter today’s date, and use “.now” to enter the time now. Use “.me” to enter your name (signature).  Alternatively, use “.sig” to enter something like “This result has been reviewed by Ju-Yoon Yoon, MD on 19/11/24 at 9:05 AM.” |
|  | Upon intra-operative resulting of the case, expand the case information for the specimen part in question, and click “Specimen Done” to mark completion of the frozen section.   - Completion is marked by a check mark beside the specimen. |
|  | Click “Prelim Intra-op" |
|  | Review the results – these data will make their way to the chart with intra-op results only, and be marked as preliminary results.   - Click “Confirm Prelim”   NOTE: You may need to assign the case to yourself prior to confirming prelim. |
|  | Completion of Intra-op consultation is indicated. |
|  | Results are viewable in Chart Review – case is marked as “Completed - Preliminary result” |
|  | To edit frozen section results later, “Intraoperative Consultation” findings are also available in Case Results |
|  | Intraop results are part of the final surgical pathology report as shown on the left (Chart Review view) |
|  | For subsequent intra-op samples after the initial frozen section, the above workflow is repeated, including clicking case parts of “Specimen Done” and clicking “Confirm Prelim” again. Re-confirming prelim results in the result status being marked as “corrected result” as shown |
|  | In case Inquiry, these events are date- and time-stamped. |

Marking a specimen as a frozen before putting in a specimen protocol creates a frozen section block and frozen HE. This creates a hold on the specimen, only after intraop diagnosis is done will it clear the hold. After the intraop is marked as done, the frozen section block and frozen slide tasks are confirmed. Frozen section block in grossing can be printed. Frozen section block cannot be selected to add tasks such extra FS slides, or smear slides.

NOTE: Intraoperative section will also be used for cytology cases, including ROSE procedures – see under cytopathology.

NOTE: Cases will be re-assigned to pathologists on different services at the time of slide delivery. Until the case is re-assigned, the intra-op pathologists will be designated in the system as the “responsible” pathologist in the Epic Beaker system.

### AP – Gross-Only Cases (foreign bodies)

|  | Orders are as “tissue exam” - need to specify source & type   - While “foreign body” removal procedure can be found in the Epic system, the procedure currently does not generate a pathology order for gross assessment. - If no block is to be generated, blocks (& slides) can be removed in Case Builder |
| --- | --- |
|  | In Case Builder, add the flag “Foreign Body” |
|  | Click on the “Add comment” box to add comment to the flag.  NOTE: transfer of custody of foreign body objects, such as bullets, can be documented here. This flag and comment can be added even after the case has been verified, by accessing Case Builder. |
|  | Flags added, including those added post-verification, can be viewed in Case Inquiry (shown on left). |

### AP – Ordering Histochemistry & IHC (Task Protocols)

|  | In Case Builder, add task to a block – Screen shows various histochemistry & IHC orders   - Multiple orders can be placed - Order of tasks cannot be changed – needs to be deleted & re-ordered - Single order can be done multiple times |
| --- | --- |
|  | Orders appear in Case Builder, placed in the order as placed above   - Click on “X” mark if accidentally ordered – can be removed even after some work has been done by lab - - Flags can be added - Using previously cut unstained slide will have to be indicated in the note |
|  | Multiple blocks can be selected by Clicking on multiple blocks, prior to clicking on “Add Tasks” |
|  | Notes/Flags can be added within Case Builder (e.g., marking as “Rush”) |
|  | Note added is seen in Case Prep Work List under the “Incomplete” column |
|  | After tasks have been ordered, for the histology lab, Orders appear in Case Prep Work List as pending tasks for histology |
|  | Slide labels indicate the different task protocols performed  NOTE: Each “trigger” for different ancillary tests is equated with a level, and the section levels for the slides may skip accordingly. |

### AP – Ordering Tasks on Previously Cut, Unstained Slides

|  | Ordering stains using unstained by deleting the protocol name in the Case Builder for unstained – can comment that this is being ordered on unstained slide, and then Save/Advance to update the system. |
| --- | --- |
|  | Changes to unstained slides are tracked (shown on left) |

### AP – Resulting

|  | “Case Results” are available from different pending lists. Click on the case of interest in the Outstanding List to view “Case Results”.   - Click on the Pencil icon to enter editing mode. - Keyboard shortcuts are available as “Alt + underlined letter” - Fields with “*” will not be included in the final report   Use of various phrases and SmartText can be helpful   - Use “.APRALLFINAL” to add different case parts (specimen) |
| --- | --- |
|  | Case Results can also be entered from Case Builder by clicking on the “Case Results” button |
|  | Case information available at top   - Hover over the flag icon to view case flag - Logging out the case will save changes – goes into sleep mode - shut down timing is customizable at role level |
|  | “Restore Results” button can be used to restore results to status before entering the editing mode  NOTE: Mnemonic function has not been validated as of the go-live date |
|  | Formatting can be controlled by clicking on the star button (All Tools) |
|  | See Snapshot, Forms, Coding, QA sections separately  NOTE: Coding will not be used presently, but is available for ICD10, SNOMED coding   - Charges can be viewed/managed |

NOTE: “Mnemonic” function may be used in some divisions, but was not explored further for uses in AP (e.g., cytotechs reporting NILMs, disclaimers).

### AP – Viewing Linked and “Other” Results

|  | “Linked” and “Other” order results are available in the “Snapshot” tab  Linked Orders may include:   - Molecular ancillary tests ordered on a pathology case (NGS, *MLH1* promoter, *MGMT* promoter methylation assays, FISH) - Other lab tests linked to the pathology case by timing of the orders   Other results, including prior pathology results, are also viewable   - The filter for “Abnormal” can be toggled on/off - currently only applicable for cytology results |
| --- | --- |

### AP – Synoptic Form (Report)

|  | From Resulting Screen, click on “Forms” (second icon)   - Left screenshot shows the buttons in their collapsed form |
| --- | --- |
|  | Choose the right Synoptic Report form to be added |
|  | Additional synoptic report can be added as above, which will appear as a separate tab |
|  | Attempting to sign out a case with incomplete synoptic report will prompt an error and incomplete data fields will be displayed as shown |
|  | Forms can be Managed, inc. Deleted & re-ordered |
|  | Macros can be setup:   1. Enter results as desired 2. Click the “play +” macro button 3. Choose macro caption of interest & click Accept     Macros can be managed under “Macro Manager” |

### AP – Synoptic Report using Macro

|  | Search > “Macro Manager” > “Create New Macro” |
| --- | --- |
|  | Choose “Form” and search under “Form” to look for synoptic report of interest |
|  | Edit the synoptic report as appropriate > Save/Accept to save |
|  | When the appropriate form has been added, the macro name should appear as a button |

### AP – Verify Results (sign-out)

|  | Check in Case Builder that the sign-out pathologist is actually assigned to the case. **The assigned pathologist’s name, and *not* necessarily sign-out pathologist’s name.** |
| --- | --- |
|  | Results are automatically saved as they are edited - “Save” icon can be clicked on to manually save. |
|  | “Restore Results” can be used to restore the version of the results *prior* to the case being saved |
|  | Advance can be used by Trainees as well |
|  | Advance   - May be used by residents - May be used by cytotechnologists - May be used for molecular results   Final verify – case is signed out and results available in chart  Prelim verify – suitable for:   - Intra-operative consultation results sign-out - Provisional autopsy report   Pend Final/Pend Prelim – may be used by residents to save results  - not used by molecular – rather advance  Verify & Require Cosign – not used at UHT |

### AP – Residents and other Trainees, including Fellows

|  | Residents are able to enter results into the “Final Diagnosis” field and order additional workup. However:   - **DO NOT** click on “Verify” - depending on the option chosen, the data may become viewable in the patient’s chart - Any ordered stains will be delivered to the assigned staff pathologist |
| --- | --- |
|  | In Case Builder, residents should enter their information under “User” for the case in Case Builder, with the Role “Additional Resident”. |

### AP – Addenda and Amendments

|  | To addend or amend a case after sign-out:   - Scan or search for a signed-out case in Case Results - Select Amendment or Addendum |
| --- | --- |
|  | Addend a Case Result   - Choose Addendum and Confirm Addendum to proceed   NOTE:  When choosing to addend, the previously report data fields (including gross description) *cannot* be altered. Altering such fields require the case to be *amended*.   - For Addenda with new additions to the gross description (e.g., new block added) - include the new grossing text in the addendum text. - Above includes “Correction History” - this should be filled out for amendments. |
|  | Select the appropriate field to Addend:   - Choose “Addendum” to add the first addendum, followed by “addendum 2-5" |
|  | Addend a Case Result   - Input comment into Addendum field. - Remember that previously reported data fields *cannot* be altered. |
|  | Addend a Case Result - Select Verify to complete Addendum & click “Confirm Final” |
|  | Amend a Case Result  For Amendments, you will need to select a reason, then confirm. |
|  | Amend a Case Result   - Be sure to enter “Correction History” to indicate detailed amendment history.     Make necessary changes and note these changes in the Correction History field, then Confirm Final.    If nothing is entered into the Correction History field, a warning will appear. It is possible to override the warning. |
|  | Viewing addenda/amendment history   - In Case Inquiry, the status becomes updated to “Amendment” or “Addendum” |
|  | Correction History can also be viewed in Case Inquiry – pathologists document how the results were altered. |
|  | Data fields corrected are marked and date/time-stamped. |
|  | Similar correction history is also viewable from the Chart Review function |
|  | Previous (to amendment) versions of the reports can also be viewed in Case Results function under “Tissue Exam - Previous Versions” |

## CYTOPATHOLOGY

|  |
| --- |
|  |

### Cytology – Cytotech Workflow

|  | **Cytology cases are listed separately from Surgical cases.**  Find a Cytology Outstanding List  EPIC >  Lab>  Outstanding List |
| --- | --- |
|  | **Cytology Outstanding Lists**  2 Cytology Outstanding Lists are available  EPIC >  Lab>  Outstanding List  Views>  AP Cytology Cases (Dept) - see all assigned and unassigned cases within the dept.  AP My Cytology Cases (Cytotech) - see cases only assigned to the logged-in user.  EPIC will likely default to one of these OL’s at start up. |
|  | **Customize an Outstanding List**  Select an Outstanding List you want to customize (ie; AP My Cytology Cases).  Views>  Settings>  In the Display tab, there are items in the  Available Columns that can be moved to Selected Columns.  Save and Run |
|  | **Resulting a Case – Snapshot Panel**  The Snapshot panel appears on the far-right side of the OL screen. There are 2 important tabs to note:  -Summary tab  -Tracking tab  The Summary tab can appear moderately different for between Pap/Gyn and Non-Gyne case. For this screenshot, a Pap/Gyn case is shown.  Review the panel for relevant ordering and clinical information/history before screening the case. |
|  | **Resulting a Case – Snapshot Panel**  This is the appearance of the Summary tab for a Non-Gyn case.  Note the slightly different components when compared to the Pap/Gyn case.  Case Notes and Flags will be displayed here, along with any Linked orders and Other orders (prior or current). |
|  | **Resulting a Case – Snapshot Panel**  The Tracking tab is important to note for slide count, to ensure the case is complete before screening.  It also lists all of the tracking events for the case.  You’re now ready to screen the case and enter your interpretations into the Resulting Panel (the middle panel on the OL). |
|  | **Entering Adequacy and Interpretations**  Specimen Adequacy and Interpretation (Primary) are selected using discrete dropdowns.  Unfortunately, with EPIC the dropdowns cannot be sorted by body site. This makes selecting the correct dropdown quite daunting. A couple of user aids have been developed to help with this process.  -Mnemonics  -Cheat Sheet |
|  | **Using Mnemonics**  Reporting codes can be specific to each body site. Employ the Mnemonics function to ensure correct codes are entered.  Mnemonics are available for Adequacy and Interpretations dropdown fields only.  (See how to enter Other Interpretations in subsequent steps below).  Select the Mnemonic button at the bottom of the Resulting Panel.  Search for the appropriate body site by typing in an abbreviation (ie; THY for Thyroid) and hit the magnifying glass to see all available options. Note there are separate mnemonics for Adequacy and Interpretations. Only 1 mnemonic can be applied at a time.  Once activated, the mnemonic will populate the Adequacy or Interpretation dropdown fields with the correct reporting code. |
|  | **Cytology Cheat Sheet**  This aid details the specific reporting codes for a particular body site.  A user can use this resource to directly select the correct dropdown without the need for the mnemonics function.  Each dropdown is also associated with a numerical code.  For example, typing in the code “64” into the Adequacy drop down field will select “Nondiagnostic/Unsatisfactory” as the dropdown, which is the correct code for the Pancreaticobiliary system. |
|  | **Entering Other Interpretations**  Other Interpretations are entered differently. EPIC does not allow for the text from discrete dropdowns to be edited.  We will enter Other Interpretations using the "Add a Comment” function.  Next to Specimen Interpretation is a small “Add a Comment” icon. Clicking on it opens a new comment box.  ***Do not mistaken this new comment box for the Specimen Comments field, which is its own separate field*** |
|  | **Use SmartPhrases**  In the new comment box, select a SmartPhrase by clicking the “List my Phrases” icon.  There are 2 sets of SmartPhrase “folders” to draw from. Be sure to check the “My SmartPhrases” checkbox, as these contain Cytology-specific terms.  Search for and select the desired SmartPhrase from the list, then click Add and Close. |
|  | **Use SmartPhrases**  Alternatively, you can also type a period “.” in front of the SmartPhrase name directly into the comment box.  For example, typing “.ai” then pressing the tab key will input “Acute inflammation present” into the comment box.  “Acute inflammation present” has now been populated in the “Add a Comment” box. |
|  | **Before Advancing or Verifying a Case**  Use the “Preview Case Report” feature to preview the case before clicking Advance or Verify.  It is either located on the toolbar or from the Action button.  EPIC has very limited options to “undo” actions. Always be careful before finalizing your diagnosis. |
|  | **Preview Case Report**  Ensure all of your inputs are accurate before Advance or Verify. |
|  | **Cytotech Sign-Out**  To sign out a negative, non-high-risk Pap/Gyn case, enter your interpretations, then click Verify.  A preview screen will appear. Review one final time, then click Confirm Final to sign-out the case.  The case will now enter the 10% QC pool to be randomly selected to get rescreened. |
|  | **10% QC Rescreen**  EPIC will randomly select 10% of negative Pap/Gyn cases for QC.  The case will appear on the “AP Cytology (Dept)” Outstanding List. The Status of the case will appear as “Rescreen”  The QC screener will assign the case to themself, re-screen the case and either Advances case to Cytopathologist or signs out the case. |
|  | **Cases that need to Advance to 2^nd^ Screener or Cytopathologist**  Negative high-risk cases will need to go to a second screener.  Screen the case, then click Advance.  Select Cytology Rescreen or Cytopathologist Review, then click Confirm Advance. |
|  | **Deliver Slides to Cytopathologists**  Use the Tracking function  EPIC>  Tools>  Lab Tools>  Tracking |
|  | **Tracking**  Click on the “Delivered” tab  Scan the slides or click Look Up to enter cases manually in the event the scanner isn’t working.  For Reason, choose “Delivered to Pathologist”  Add a note in Comments box. For example, “Delivered to NGL”  Click Track |
|  | **Sending Specimens and Cases to SJHC/SMH**  Use Packing List Editor  EPIC>  Lab>  Packing List Editor  Create a new Packing list or open an existing one.  Choose “Unity Anatomic Pathology Packing List” as the List Type.  Click Create New |
|  | **Packing List Editor**  Select the Destination  Scan or manually add the specimens and/or cases.  Add a comment under the Details tab if needed.  ***Be sure to check the “Select All Tests” check box to ensure the test is at the correct resulting lab. ***  Select Ready to lock the Packing List   - The manifest will automatically print out.   Click Picked Up |
|  | **Packing List Editor**  The Packing List summary can be seen in the Snapshot tab. |
|  | **Receiving Specimens and Cases from SJHC/SMH**  Use Case Receiving  EPIC>  Lab>  Case Receiving  Several options available:  Option 1 - Scan the Packing list bar code to list all of the items from the packing list manifest. Use the mouse and select all of the items by holding the Shift key, then click Receive.  Option 2 – Scan or manually select one item at a time rather than scanning the Packing List barcode. This method is slower but may be more accurate.  Option 3 – Choose the Expected tab and locate/click-on the specimens and/or cases, then click Receive. |
|  | **EPIC is “Location Sensitive”**  Specimen/Slides are location specific and if not tracked correctly, can cause issues.  In this example, the Cytotech cannot result this case at SMH as the case is “located” at SJHC. |
|  | **Storing and Releasing cases for Review**  Use Tracking  This function places a time stamp on the specimen/case.  Choose the Delivered tab. Scan the specimen/case or select Lookup to enter them manually.  Select “Cyto Slides Storage – In” or “Cyto Slides Storage – Out” as the Reason.  Add any relevant notes in Comments (optional), then click Track. |
|  | **Adding SmartPhrases**  Use SmartPhrase Manager  EPIC>  Tools>  SmartText Editor>  SmartPhrase Manager |
|  | **SmartPhrase Manager**  Select the Add SmartPhrase button and add a New Phrase. |
|  | **SmartPhrase Manager**      On the text box ruler, move the indent to the right by 1 notch (this is to ensure proper formatting on the final report).  Enter a SmartPhrase as you want it to appear in the field.  Under Settings, enter a short-form Name for the SmartPhrase (ie; LNADEQ for Lymph Node Adequate).  The Short-form name can be used within text box for quick entry by typing in period “.” then the Short-form name (ie; “.LNADEQ”).    Enter a Description of the SmartPhrase (can just use the actual SmartPhrase itself to be the description.    Allow Sharing and Editing of the newly created SmartPhrase with other users.    Click Accept |
|  |  |

### Cytology – Cytopathologist Workflow

|  | **Outstanding List**      Cases to result/review will appear in the Outstanding List.    In most instances, the Outstanding List will be the default starting page. If not, it can be found here:  EPIC>  Lab>  Outstanding List      Alternatively, there is an Outstanding List button on top-left of screen |
| --- | --- |
|  | **Outstanding List**  Use “Views” to see other OL’s  Views>  AP My Cytology Cases      Select AP My Cytology Cases or AP My Cytology Cases (Pathologist) to see cases assigned to you. |
|  | **Sort & Filter Columns in Outstanding List**      Sort various columns by left-clicking the column header to sort alphabetically or ascending/descending.    Filter or unfilter columns by right-clicking the column header. |
|  | **Review Ordering Information and Clinical notes before Resulting**  Scan the slide or double-click the case from the list.  Use the Snapshot Panel on the right side of the screen. There are 2 tabs:  -Summary tab  -Tracking tab    Under the Summary tab, note the Specimen Source and Protocol, Clinical Information/Comments, Cytotech Screening Interpretations, any Linked Orders, and Other concurrent or prior orders.    Any prior Abnormal cases (Cytology only, not Surgical) can be filtered by selecting the “Abnormal” button. |
|  | **Review Ordering Information and Clinical notes before Resulting**      Under the Tracking tab, note the Slide count, Case Tracking information, and Provider ordering information. |
|  | **Customization of the Snapshot Panel Toolbar**      Use the Wrench button to modify/customize the Snapshot Toolbar. |
|  | **Chart Review**      Select Chart Review to see patient chart and history.    If the Chart Review button is not available from the toolbar, select it from the Action Button on the top-right of screen. |
|  | **Chart Review**      See prior lab tests and encounters. |
|  | **Entering Case Results**      To enter a Case result, select the Edit button in the Resulting Panel. |
|  | **Resulting Panel**  There are multiple components in the Resulting Panel:  Clinical Information – comments from the ordering physician appear here; this text can be edited.  Specimen Adequacy and Specimen Interpretation – these are entered using discrete dropdowns. Use Mnemonics or Cheat Sheet to ensure correct selection is made (will be discussed further down).  Rapid Assessment – ROSE information is shown here. Text needs to be first entered in Case Builder, but can be later edited here.  Gross Description – Text needs to be first entered in Case Builder, but can be later edited here.  Internal Comment – communication field for Cytotech to Pathologist. Inputs will not appear on Final Report but will appear in Case Inquiry.  Preliminary Diagnosis, Microscopic Description, Correction History – free text fields, to use where appropriate. |
|  | **Using Mnemonics**      The discrete dropdown options for Adequacy and Interpretation are not organized by body site. To ensure the correct dropdowns are selected, employ the use of Mnemonics.    Click the Mnemonics button at the bottom of the Resulting Panel.      Search for the appropriate body site abbreviation (ie; THY for Thyroid) and hit the magnifying glass to see all available dropdown options. Note there are separate mnemonics for Adequacy and Interpretations. Only 1 mnemonic can be applied at a time. |
|  | **Entering “Other Interpretations”**      In EPIC, Other Interpretations are not chosen with dropdowns. Dropdowns cannot be easily edited with free text, so SmartPhrases will need to be employed instead.  To enter an Other Interpretation, click on the “Add a Comment” icon to the right of the Specimen Interpretation field.    ***Is it important to note that this box is not to be confused with the “Specimen Comment” field”, which is its own field entirely. *** |
|  | **Entering Other Interpretations**      Clicking the “Add a Comment” icon opens a new text field called “Comment” |
|  | **SmartPhrases**      Click on the “List My Phrases” button.    Cytology-specific “Other Interpretations” SmartPhrases have already been created.  Make sure the “My SmartPhrases” checkbox is checked off – the Cytology-specific SmartPhrases live here.  Search for a SmartPhrase by typing in an abbreviation or keywords. Select the desired SmartPhrase, then click Add and Close. |
|  | **SmartPhrases**  The SmartPhrase now appears in the “Add a Comment” box and can now be edited with free text if necessary. |
|  | **Verifying the Case Results (signing-out cases)**      There are several Verifying options:    Final Verify – releases results to the patient’s chart and sends the results to the patient’s primary care provider (most common selection).    Prelim Verify – sends results to the patient’s chart with a preliminary status.    Pend Final – changes the case Status to “Pend Final”    Pend Prelim –changes the case Status to “Pend Prelim”    Verify & Require Cosign – a required cosigner is requested before sign-out. |
|  | **Preview Case Results before Final Verify**      A preview screen will appear after Final Verify is selected. Review for accuracy, then click Final Confirm.    Alternatively, using the “Preview Case Report” function is also recommended. This function shows a preview of the exact appearance of the Final Report.    Preview Case Report can be found on the toolbar or under the Action button. |
|  | **Add a QA Event**      Select QA tab from the Snapshot Panel |
|  | **Add a QA Event** |
|  | **Amend/Addend a Case Result**  Scan or search for a signed-out case in Case Results  Select Amendment or Addendum |
|  | **Addend a Case Result**  For an Addendum, first Confirm the Addendum. |
|  | **Addend a Case Result**  Select the appropriate field to Addend |
|  | **Addend a Case Result**  Addend the case, then click Verify to complete Addendum. |
|  | **Addend a Case Result**  Preview the Addendum and Final Report, then click Confirm Final. |
|  | **Amend a Case Result**  For a Amendment, select a Reason then Confirm. |
|  | **Amend a Case Result**      After the amendments are made, be sure to add a note into the Correction History field.    If nothing is entered into the Correction History field, a warning will appear. However, it is possible to override the warning by selecting Clear.  Click Confirm Final |
|  | **Looking up Cases**  Use Case Inquiry  EPIC>  Reports>  Lab Reports>  Case Inquiry |
|  | **Case Inquiry**  Can see:  Specimen Protocol, Tasks  Provider  Case Tracking  Order Details  CC List (can add/modify)  Case Results |
|  | **Print Final Report - from Case Results**  EPIC>  Lab>  Case Result |
|  | **Print Final Report - from Case Results**  Enter Case number and Accept |
|  | **Print Final Reports - from Case Results**  Locate “Preview Case Report” from the Action button . |
|  | **Print Final Reports – from Case Results**  Preview and/or Print |
|  | **Print Final Reports – from Chart Review**  Select Results Review tab |
|  | **Print Final Reports – from Chart Review**  Select the desired Laboratory Result from the Lab Reports list |
|  | **Print Final Reports – from Chart Review**  Scroll down and select Create specimen report |
|  | **Print Final Reports - from Chart Review**  Create New Reports  Select the Recipient of the Final Report:  Department, Patient, or Providers.  Click Add to List |
|  | **Print Final Reports – from Chart Review**  Preview and/or Print |
|  | **Ordering Blocks and Special Stains**  Use Case Builder  Scan/select the case, then right-click to open Case Builder.  Alternatively, select the case, then click Case Builder from the toolbar or from the Action button. |
|  | **Ordering New Blocks**      To order a brand-new Block, be sure to unclick from any of the existing blocks first.    Add a new Block by clicking the “Add Tasks to” search field. |
|  | **Ordering New Blocks**      Search for desired Block(s) from the list (can select multiple at a time).  The ”Peach Cassette” block is for Cytology (not shown on screenshot)    If more than one Block is needed, adjust the quantity on the right side of the window.  Click Accept |
|  | **Ordering Special Stains**      Select the specific Block for the Special Stain.    In this example, “1B” is the selected Block. The Special Stain will be prepared from this Block.  Search for the desired Special Stain from the “Add Tasks to” search field. |
|  | **Ordering Special Stains**      Search and select desired Special Stain (can select multiple stains at a time).    Enter the quantity of each special stain on the right-side panel.  Click Accept    The order for the Special Stains will now appear on the Case Prep Work List (CPWL) for the Histotechnologists to process. |
|  | **Adding Flags and Notes to a Case**      Case Flags can be added from the dropdown options.    Special instructions for the Histotechnologists can be noted for any specific Block or Special Stain (EPIC calls these “tasks”). |
|  |  |

Resulting

|  | Various fields   - “*” designates empty fields |
| --- | --- |
|  | Case assigned within Case Builder |
|  | “Assign” button can also be used for assigning |
|  | Batch Assign from Pending list   - Choose multiple cases - Assign to user |

Adding blocks

|  | Performed within case builder   - May be performed reflexively based on specimen type |
| --- | --- |

### Cytology – Ordering and MLA Workflow

|  | **Ordering a Cytology Test for In-Patients**  Use Patient Station  Search for the Patient   - MRN, First/Last Name |
| --- | --- |
|  | **Ordering a Cytology Test for In-Patients**  Select correct patient from list and Accept |
|  | **Ordering a Cytology Test for In-Patients**  Select Encounter, then double-click Admission |
|  | **Order Inquiry tab**  For first time users, a Report Settings window will pop up   - Select “Lab Order Inquiry All” - Run |
|  | **Orders tab – New Test**  Under the Orders tab, click New |
|  | **Select a Cytology/Pathology Test**  Pathology Tests will appear under “Pathology” in the “Lab” section   - Non-Gynecologic Cytology - Fine Needle Aspiration Cytology - Pap/Gyn Test |
|  | **Select a Pap/Gyn Test**    Pap/Gyn Order Composer Questions   - LMP - Clinical Indication - History - HPV Status - Specimen Type, Source   Accept and Sign |
|  | **Select a Non-Gynecologic Test**    Non-Gyn Order Composer Questions   - Specimen Source - Clinical Information - Comments - Specimen Type   Accept and Sign |
|  | **Select a Fine Needle Aspiration Cytology Test**    FNA Order Composer Questions   - Clinical Information - Specimen Source   Accept and Sign |
|  | **Prepare to Collect Specimens**    Return to Order Inquiry tab   - Select the order from Pathology and Cytology Lab heading   Click Collect Specimens |
|  | **Print Specimen Labels**    Additional Questions (Pap only)   - LMP - HPV Reflex?   Click Print Labels |
|  | **Collect the Specimen and Complete Order**    Attach printed label to Specimen container.  Collect the Specimen, then scan the Specimen label to confirm the collection.  Click Collect specimen and Accept. |
|  |  |
|  | **Receiving the Specimen in the Lab (Option 1 – Case Builder)**  Open Case Builder and scan the Specimen bar code.  The Specimen is now marked as Received and is now ready to be Accessioned and Grossed in Case Builder. |
|  | **Receiving the Specimen in the Lab (Option 2 – Case Receiving)**  EPIC>  Lab>  Case Receiving |
|  | **Receiving the Specimen in the Lab (Option 2 – Case Receiving)**  Scan the Specimen, then click Case Builder from the toolbar.  The Specimen is now marked as received in the lab and can now be accessioned and grossed. |
|  | **Receiving the Specimen in the Lab (Option 2 – Case Receiving)**  The Specimen can also be manually received from the Expected tab, in the event the barcode or scanner is not functional. |
|  |  |
|  | **Accessioning and Grossing**  Use Case Builder  (Shown is a Pap/Gyn Test)  Accession the case   - Accession number is created for the case. This number can be adjusted with the “+” button adjacent to it. - Review the Order Information for completeness and correctness from the right-side panel. - Assign appropriate Protocols + Tasks. - Apply and Flags or Case Notes if necessary. - Save/Accept will automatically print 3 or more Container labels. Slide labels will be printed in the following step in CPWL. - If there is a Cassette to be printed, this is done in the Grossing tab (use the Print New button). - Return here to reprint any label later if needed. |
|  | **Accessioning and Grossing**  (Shown is a Non-Gyn Test)  Enter Gross Description in Gross tab   - Use SmartPhrases and/or free text. - If there is a Cassette to be printed, this is done here by pressing “Print New.” - MUST click Specimen Done when complete.   Use Builder tab to enter Protocol + Tasks  Save/Accept |
|  | **Accessioning and Grossing**  (Shown is an FNA Test)  Enter Gross Description in Gross tab   - Use SmartPhrases and/or free text. - MUST click Specimen Done when complete.   Enter Intra-Op if there is a ROSE study   - Use SmartPhrases and/or free text. - Manually input data (pass #, etc) - MUST click Specimen Done when complete.   Use the Build tab to enter Protocol + tasks.  Save/Accept |
|  | **Ordering Additional Blocks**  Use Case Builder  Unclick from Stain/Process (1A)  Select “Add Tasks to” |
|  | **Ordering Additional Blocks**  Select the desired Block and Accept.  The “Peach Cassette” Block is used for Cytology. |
|  | **Ordering Special Stains/Tasks**  Select the Block (1B)  Select “Add Tasks to” |
|  | **Ordering Special Stains/Tasks**  Select the desired Stains/Tasks, can select multiple under “quantity” on the upper-right side of window  Click Accept  Repeat steps to add any additional Stains/Tasks |
|  | **Save and Accept**  Once Protocols and Tasks are all inputted, slick Save/Accept. |
|  | **Printing Slide Labels**  Use Case Prep Work List  EPIC>  Lab>  Case Prep Work List |
|  | **Case Prep Work List**  Select the correct Case Prep WL under Views   - Case Prep Work List (Cyto) - Or your own custom created version. |
|  | **Confirming Tasks and Printing Slide Labels**  Scan the Container label  The case and its associated tasks will appear on the right side of the screen. The tasks should also be checked off.  Toggle the “Print Labels/Confirm” button to Print Labels and click it to print the Slide labels.  Then toggle the “Print Labels/Confirm” button back to Confirm. Scanning the slide label will automatically remove the task from the CPWL.  Remaining tasks (workload units) may still remain. These can be selected manually with the mouse and clicking Confirm will remove them from the list.  Confirming cases is required to ensure Slide count is correct. |
|  |  |
|  | **Ordering for Referred-In Patients (ie; Cambridge)**  Use Requisition Entry  EPIC>  Lab>  Requisition Entry |
|  | **Requisition Entry**  Select the Submitter (ie; Cambridge Memorial)  Enter patient name or create a new patient.  Enter Authorizing and Ordering Physicians |
|  | **Scan the Requisition**  Use the Scan button to scan in the requisition ONLY! The remaining documents (ie; ROSE evaluation forms) are to be scanned in later a step. |
|  | **Create an Order**  Select Add an Order |
|  | **Select a Test**  Fine Needle Aspiration Cytology  Non-Gynecologic Cytology  Pap/Gyn Test |
|  | **Order Composer and Clinical Information for** **Requisition Entry**  Questions   - Specimen Type - Specimen Source - Priority (Routine vs. STAT) - Order Comments (all transcribed Clinical Information goes here)   Select Case Builder to build, accession, and gross the case. |
| [INSERT SCREENSHOT HERE] | **Scanning ROSE Evaluation forms**  Use the “Case Results” button on the Case Builder toolbar to move to the Outstanding List. This function can also be found in the Actions button.  On the Outstanding List, in the middle panel (the Resulting panel), scroll to the very bottom.  Click Add Scan to scan ROSE Evaluation forms and other documents. ***Be sure to choose “Internal” and not “Reported”*** |
|  |  |
|  | **Specimen Rejection – when specimen has not yet been accessioned**  Use Specimen Inquiry  EPIC>  Lab Reports>  Specimen Inquiry>  Specimen Inquiry (By Specimen)  Must have sufficient access level to make these changes |
|  | **Specimen Rejection – when specimen has not yet been accessioned**  Specimen Lookup  Scan specimen bar code |
|  | **Specimen Rejection – when specimen has not yet been accessioned**  Action Button  Select Cancel |
|  | **Specimen Rejection – when specimen has not yet been accessioned**  Select Reason for cancellation  Select Yes |
|  | **Specimen Rejection – when case has been accessioned**  Use Case Builder  Action Button  Select Cancel  Case must be saved before it can be cancelled with this method |
|  | **Specimen Rejection – when case has been accessioned**  Select Reason for cancellation  Select Yes |
|  |  |
|  | **Reprinting Labels**  Use Case Builder  Click Labels from the toolbar or select from the Actions button. |
|  | **Reprinting Labels**  Select which labels to print   - Entire Case Labels - Specimen Labels - Cassette Blocks - Slide Labels   Print Labels |
|  |  |
|  | Results   - “Other Orders” to view old results - Filter can be applied but legacy data not flagged appropriately - “Clinical information” - to be modified to be pulled from req |
|  | QA for discordant cases with Cytotech will have to be performed manually |

### Cytology – GYN cytology

- Built-in HPV Reflex logic has been disabled, now granting 100% control/responsibility to the pathologists. It was determined that the built-in reflex system is somewhat flawed and will require extra effort to massage it down to our specific needs. Seeing that Pap/Gyn tests will be discontinued soon, it didn't make sense to expend resources to this.
  - HPV Ordering, Sendout Bench, Package List Editor, HPV Oustanding List, and Resulting all work. How these results are displayed on the Final Report is yet to be determined.

### Cytology – Pap/Gyn Order Composer Questions

|  | Select a Pap/Gyn Test    Pap/Gyn Order Composer Questions   - Routine or STAT - LMP - Routine or Colposcopy or Other - Clinical Indications - History - HPV Status - Specimen Type, Source   Accept and Sign |
| --- | --- |

### Cytology – Non-Gyn Order Composer Questions

|  | Select Non-Gynecologic Cytology  Composer Questions   - Specimen Source - Clinical Information - Specimen Type   Accept and Sign |
| --- | --- |

### Cytology – FNA Order Composer Questions

|  | Select Fine Needle Aspiration Cytology      FNA Order Composer Questions   - Clinical Information - Specimen Source - Specimen Type is defaulted to Non-Gyne Cytology   Accept and Sign |
| --- | --- |

### Cytology – Second Pathologist Review

10% of cases require 2^nd^ pathologist review, *prior* to final verifying the case. This was changed from the system’s default setting of 2%. This was enabled for *all* cytopathology cases, including STAT cases. This task will show up in “FYI” section of the side panel, and in the “Follow-up” worklist – see under “Cases Requiring Second Pathologist Review” view of the Follow-up Work List.

|  | **Cases Requiring a Second Pathologist Review**      EPIC has built in a QA function where 10% of cases will require a 2nd pathologist review, prior to final verifying the case. |
| --- | --- |
|  | **Cases Requiring a Second Pathologist Review**  A hard hold will be placed on the case until the case is reviewed by a 2nd pathologist.  The case will move to a Follow-up Worklist |
|  | **Cases Requiring a Second Pathologist Review**      EPIC>  Lab>  Follow-up Work List |
|  | **Cases Requiring a Second Pathologist Review**  Views>  Cases Requiring Second Pathologist Review |
|  | **Cases Requiring a Second Pathologist Review**      Review the case, document any notes, and click Complete.    Case will drop off the Follow-up WL    Return to OL to Final Verify the case |
| [INSERT SCREENSHOT HERE] | **Bypassing Second Pathologist Review**  It is possible to bypass a Second Pathologist review.  Click Verify. An alert will appear, but next to the alert is a “Clear” button. Click it to override the hard stop. |

### Cytology – Sendout HPV test

|  | Ordering HPV Test (Cytopathologist only)  100% control and responsibility is given to the Cytopathologist  Order HPV Test? Select Yes  Verify |
| --- | --- |
|  | Ordering HPV Test (Cytopathologist only)  Selecting Verify will create a pop up note confirming HPV Test has been ordered.  Specimen will now go on Sendout Bench list for HPV Sendout to Dynacare.  ***HPV test request is initiated when the Advance or Verify button is selected, not when Confirm Final is selected***  HPV tests can be cancelled at AP HPV Sendout Bench OL before test is sent to Dynacare. |
|  | Sending out HPV Test to Dynacare – Sendout Bench  Ordered HPV Tests will automatically go to the Sendout Bench  EPIC>  Lab>  Sendout Bench |
|  | Sending out HPV Test to Dynacare – Sendout Bench  Select HPV Tests to send  Add Tests to Packing List |
|  | Sending out HPV Test to Dynacare – Sendout Bench  Packing List Lookup window appears  Select Unity Lab AP Dynacare HPV Packing List from List Type  Create New |
|  | Sending out HPV Test to Dynacare – Sendout Bench  Finalize HPV Packing List   - Ready - Print (automatic) - Picked Up |
|  | Result HPV Tests when results are received from Dynacare - AP HPV Send Out Outstanding List  Views>  AP HPV Send Out  Admin staff can enter results of HPV Test   - Select case - Pencil/edit icon - Enter results   Use Save icon  ***Do NOT Verify***  Workflow requires Pathologists to periodically check the AP HPV Send Out Outstanding List for cases pending sign-out. Currently, there is no reflex to notify the Pathologist that the HPV result has been entered. |

## AUTOPSY Workflow

|  | |
| --- | --- |
|  | Deceased patients are marked by the Leaf mark |
|  | Order as “Autopsy Exam” |
|  |  |
|  | Order appears in “Labs” as other pathology tests |
|  | Same process for perinatal autopsy |

### Autopsy – Body Tracking

|  | Morgue tracking – body information appears in the Decent Affairs tab |
| --- | --- |
|  | Morgue Patient List   - No place in Epic to document viewings; this workflow is managed by spiritual care staff - Discharge: Patients will appear on the morgue list if they are discharged as deceased, even without physician notes - Porter Requests: There is no automated porter request for the morgue. The request must be made manually, either as part of the discharge transport process or as an ad-hoc request after discharge. The unit is responsible for ensuring proper discharge from the unit - morgue list is accessible to anyone who can view the inpatient patient list, which includes clinicians and unit staff. |
|  | Body is released from the morgue for autopsy workup |
|  | Body is released to the family  Belongings typically accompany the patient to the funeral home to ensure they don't remain in the unit. The morgue attendant will document this. Security staff, who are on duty 24/7, handle the release of patients. They will continue to use paper forms for double-checking, though computers will also be available for documenting necessary information. |

Of note, transport of and tracking of deceased patients (including fetuses) was headed by the EVS department. The above data are based on demonstration from the Epic working group, coordinated by EVS. The above workflow was not validated by the pathology division.

### Autopsy – Provisional Report

|  | Provisional reports are signed out by:   1. Enter data into “Final Diagnosis” 2. Copy the same data into “Provisional Diagnosis” 3. Click on “**Prelim Verify**” (and NOT Final Verify) - results will be available in chart, but not as final report 4. Need to enter password to sign out the reports   “Final Diagnosis” text is revised for the final report. |
| --- | --- |

Of note, the data entered as above will be tracked (see Inquiry), but the different texts will be overwritten upon issuing of the final report.

### Autopsy – Final Report

|  | “Final Diagnosis” text is revised for the final report.   - Need to enter password to sign out the reports |
| --- | --- |

Reporting wise, note:

- Ancillary results to be added under “Final Diagnosis” (without separate data field).
- Provisional diagnostic data as above.
- Block template (currently under “Histo data” in CoPath) will be combined with internal exam text
  - Previously setup with template that contained various organ weight & dimensions, with normal ranges
- Following data fields are not reported:
  - Internal exam - not reported
  - External exam - not reported
- Neuro gross description will also be added to Internal/External exam text
- Neuropathology diagnosis - new section to be added

### Autopsy – Ancillary Workup

|  | Ancillary workup may include:   - Cytogenetics (e.g., umbilical cord) - Microbiology (e.g., tissue culture) - Toxicology (e.g., drugs)   Take note of the patient’s MRN and enter Lab > “Requisition Entry”   - Submitter = SMH or SJHC - Auth. & Ord. Provider = Pathologist on the case |
| --- | --- |
|  | Enter additional information   - GA and mother’s information relevant for perinatal case - Enter date of death |
|  | Choose the desired order & click on “Create Specimen”   - Specify “Specimen Type” & “Specimen Source”   - Of note, Type is restricted to Bone, Cartilage & Tissue - Priority = Routine |
|  | Click the downward chevrons to expand the ordering screen   - Indicate the Autopsy case number in “Lab Order Comments” |

Of note, for send-out tests, please see the “Sendout” workflow

## HEMATOPATHOLOGY (HP) Workflows

NOTE: Various hematopathology workflows were not validated as part of the wider AP Epic Beaker workflow validation study. Some aspects of this workflow may be discrepant with workflows to be coordinated with hematology. Inquiries should be directed to Dr. Sasan Zandi.

### HP – Peripheral blood review

|  | Several different outstanding worklists are available for hematology, including SMH Hematology (hematopathologist review) |
| --- | --- |
|  | Click on the pencil icon in Case Resulting to enter comment |

### HP – Bone Marrow Panel

|  | Ordering for “Bone Marrow Panel” - search for “bone”   - Specify which components are required |
| --- | --- |
|  | - For molecular & cytogenetics components, additional information are required – both can be ordered on the bone marrow and/or the peripheral blood - Certain options will require additional information – to be inserted in the adjacent Comment box |
|  | Specimens will be received by? |
|  | Outstanding cases are viewable in the “Bone Marrow” list. In the case resulting screen, click on the pencil icon to enter data. |

Ordering instructions from Sasan:

|  | 1. Book the marrow (MLT time) on the new scheduling, this is managed by hematology (Charmaine) 2. To Place orders Go to “Manage Orders” |
| --- | --- |
|  | 1. Type “bone” NOT “Marrow” in the search area; select “BONE MARROW PANEL” |
|  | 1. You should be able to see this panel: |
|  | 1. Select the tests that you want to order 2. Accept order and Sign it. |
|  | 1. Then you need to collect the specimens under order inquiry |
|  |  |
|  | 1. At this point you can **print labels and please MAKE SURE YOU SPECIFY THE SITE AND SIDE OF THE BIOPSY** in comment section of “bone marrow exam” 2. Stick the labels to the appropriate tube and fill the paper req for UHN and NYGH and DONE! |

### HP – Flow Cytometry

|  |
| --- |

|  | Ordering is available under ”Flow Cytometry, Immunophenotyping” |
| --- | --- |
|  | Flow cytometry can also be ordered from pathology as a “trigger” (Flow (Trigger)) from Case Builder |
|  | Two outstanding lists are available under Flow Cytometry – AP or CP |
|  | Flow cytometry results are entered by technologists for review.   - Sign out results in a report, separate from the surgical pathology report (in AP cases) - surgical case should be listed under “Linked Order” in the order snapshot/summary |

### HP – Lymphoma Protocol

|  | Order “Tissue Exam” - insert comment “Lymphoma Protocol”   - Clinical team should call the lab to notify   In Case Builder, choose “Lymphoma Protocol” protocol:   - “Flow (trigger)” triggers flow cytometry test - Print a copy of Specimen Container label for the RPMI tube |
| --- | --- |
|  | Insert grossing data into Intraoperative Consultation |
|  | For cases where a separate container is received and *all* of the container content is submitted for flow cytometry, *delete* the blocks and slides in Case Builder by clicking on the “X” marks beside the block and slide(s). |
|  | In Case Prep Work List, go to the case and find the Flow (Trigger).  Click “Confirm” to confirm the trigger to initiate the downstream workflow.  RPMI tube is handed to the accessioning staff for delivery to Flow Cytometry  At Flow, a new “test” will be created, using their own F number wheel – results will be reported separately by hematopathologists |

## MGP (MOLECULAR GENETIC PATHOLOGY)

### MGP – General

|  | Ordering molecular – 3 pathways:   - “Task protocol” for pathology cases (shown on left, search query = “Molecular”)   Note: Prior “RAS molecular” is now renamed to be used for send-out molecular tests (curls or slides) |
| --- | --- |
|  |  |

For non-MGP pathologists, please use the order set. If a 2^nd^ MGP assay is requested on a case, please notify the MGP pathologist, and the order will be placed appropriately (either as a new set, or “trigger-only” orders)

### MGP – Accessioning & Receiving

|  | Check for “linked orders” - DNA may have already been extracted for the particular specimen.  A MGP test can be ordered without sections by choosing the “(trigger)” option for the relevant test in case builder.  Although the trigger does not result in unstained slides being cut, a label can be printed, and the order has to be confirmed in the “Case Prep Work List” |
| --- | --- |
|  | In Outstanding List, click “Molecular Diagnostics” - contains NGS & FISH cases  In Outstanding List, click “Molecular Pathology (PCR)” - has outstanding cases for:  MGMT, MLH1, JAK2, FVL, Prothrombin G20210A, and APCR |
|  | Unstained slides and curls are received in the “Tracking” function – look under:   - Views = “Extract” - Reason = “Received in Molecular”   Scan the barcode or lookup the specimen ID to apply the tracking status to “Received in Molecular” - automatically records received date, time, and person |
|  | Scanning the case (e.g., slide labels) results in date/time-stamping of the case to the status “Received” under Specimen Tracking   - The case will remain on the Outstanding List |
|  | After completing the step within the resulting pane, scan the label again to advance the status.   - NOTE: in the chart review, the case status will remains as “Active - In process” |

- NOTE: the scanner must be setup for Epic Beaker (see under “[Scanner](#_Scanners)”)
- Look under “Inquiry” for date/time stamps
  - Specific fields for “date_received”, “time_received”, “person_received” have now been made obsolte with the tracking function
  - Also obsolete are date_submit, and date_signout data fields

### MGP – Batch Editor

|  | Search “Batch Editor”, or Lab > Batch Editor   - Batch editor can also be accessed from Batch List and double clicking on the batch |
| --- | --- |
|  | Click “Create New” and choose appropriate assay batch   - A new Batch ID is assigned |
|  | Batch Editor Template can be revised |
|  | Samples available for adding to the batch are shown on the left |
|  | Main screen lists the samples and the 96-well plate layout   - Click on the sample of interest and the appropriate well on the 96-well plate > click “Add Selected” to add the sample to the plate   Assays run in replicates need to be “repeated” within the assay setting – otherwise, cases can be only added once into a single batch |
|  | Click on “Quality Control” to add assay controls. |
|  | Comment can be added on the panels to the right |
|  | Sample can be “moved” to a different well by clicking the sample, entering the new slot number, and clicking on “Move” |
|  | Click “Clear Slot” to remove the sample from the batch |
|  | Plate labels can be printed under “Labels”.  Click on “Ready” once batch is complete.   - This locks the plate. However, the batch can be made “Unready”.   Plate maps can be printed under “Documents”. |

NOTE: This function is currently unvalidated as of go-live. Optimization and adding testing are needed.

### MGP – Repeating steps/assays

|  | In general, repeating steps require: 1) going back within Beaker to designate the new place in the workflow, and 2) choosing the data field(s) of interest to be repeated and clicking on “repeat”. |
| --- | --- |
|  | Click Repeat > Launch Repeats Activity  Clicking the return arrow to designate the data field of interest, allowing the data field to now allow for two values |
|  | Clicking on the number “2” click the appropriate radio button to enter results from each round of repeats |
|  | “Repeat All” allows for all data fields to be duplicated. |
|  | Repeating assays or components of assays already signed out require sign-out director to click “Result Correction” |
|  | Akin to amendments in surgical pathology, reason for correction is entered. |

### MGP – FISH, HER2

|  | - HER2 FISH can be ordered as a task in Case Builder   - Order = “Her-2 FISH” - HER2 (trigger) must be added |
| --- | --- |
|  | - Task appears to Case Prep Work List – both slide & Trigger must be processed by the histotechnologist by clicking on “Print Labels” and then “Confirm” |
|  | - Case now appears under the outstanding list for “FISH (1) Slide Prep” - A new ID is assigned, corresponding to the FISH order |
|  | - Subsequent steps are 2) Drop Probes, 3) Probe Hybridization, 4) Main scorer, 5) Review by Pathologist, and 6) Pathologist Sign-Out - Progress through each step by clicking on “Complete Step” |
| (see under [Repeating steps/assays](#_MGP_–_Repeating)) | - Processing step may be repeated |
|  | - 1^st^ scorer enters results - Additional HER2 FISH data – indicate whether Aneusomy and/or Heterogeneous signals are observed - Representative image is attached by clicking on “Import Documents” |
|  | - 2^nd^ (and 3rd) scorer can be trigger by “Repeating” results – Click “Repeat” (arrow) and choose “Launch Repeats Activity” |
|  | - For 2 scorers, repeat as follow: - HER2/NER FISH (MLT score) - repeat x2 (1 additional observer + 1 set for average) - HER2/CEP17 ratio – repeat x2 - Average HER2 signals per nucleus – repeat x2 - Average CEP17 signals per nucleus – repeat x2 - Number of invasive tumour cells counted – repeat x1 - Additional HER2 FISH data – repeat x1 |
|  | Click on the Save icon and close the case.   - “Review by Pathologist” step is designated for pathologist scoring, if necessary - Place the scoresheet in the pathologist’s mailbox   **SAVE – DO NOT VERIFY** results – result verification is reserved for pathologists |
|  | For pathologists, results will NOT appear in the “AP My Surgical Cases” - the case must be found in “FISH (7) Pathologist Review and Sign-out" outstanding list. |
|  | Representative photographs are viewable in the bottom-most section of the resulting pane – make sure the photographs are set to “Internal” (i.e., not to be part of the report). |
|  | Surgical pathology results are viewable by hovering over the Pathology ID. You can also jump to the case by clicking on the hyperlink. |
|  | Click “Verify” > “Final Verify” > “Confirm” to sign-out the FISH report. |

### MGP – FISH, 1p/19q

|  | - Ordering as a task within Case Builder - FISH of BCL2, BCL6, and MYC are paused and orders are obsolete - FISH order appears in case prep worklist (as generic FISH) - The case will now appear in outstanding list under “FISH (1) Baking” - “(trigger)” must be confirmed at histology end for cases to show up |
| --- | --- |
|  | 1p/19q Final Interpretation = co-deletion status  Summary of counts – each for 1p36/1q25 & 19q13 – enter number of nuclei (& percentages are calculated) |
|  | For pathologists, results will NOT appear in the “AP My Surgical Cases” - the case must be found in “FISH (7) Pathologist Review and Sign-out" outstanding list. |
|  | Surgical pathology results are viewable by hovering over the Pathology ID. You can also jump to the case by clicking on the hyperlink. |
|  | DO NOT click “Verify”. Rather review the results, and jump to the surgical case. In the surgical case, click “Addendum”.   - Click “Complete Step” to have the case fall off the outstanding list. The   Enter the FISH results into an addendum, and verify the addendum. |

### MGP – DNA extraction

|  | For different assays that involve DNA extraction, document:   - slide_received = # of slides received - slide_scraped = # of slides scraped for extraction - date_extracted - storage_location = freezer box location for the extracted DNA |
| --- | --- |
|  | For assays that utilize the Qubit assay for DNA quantification, enter:   - DNA_qubit = DNA concentration - DNA_qubit_lot = lot # for the Qubit assay |
|  | For assays that utilize the Denovix assay for DNA quantification (e.g., *MGMT* promoter methylation assay), input:   - DNA_denovix = DNA concentration - DNA_denovix_260280, DNA_denovix_260230 = 260/280 and 260/230 ratios   NOTE: if an order was placed as “trigger only” (i.e., no designated sections or curls ordered with the test), input the *Test* ID for the other molecular test in the “comment_sample” field |

### MGP – *MGMT* promoter methylation assay

|  | - Ordering available under tasks – curls or slides |
| --- | --- |
|  | - Choosing slides automatically triggers protocol for unstained x10 + H&E slide, along with MGMT trigger - Confirming the MGMT (Trigger) order from Case Prep Work List (by histotech) moves the order from histology to molecular - Confirming stamps the work done with date and time |
|  | - Order now appears under “MGMT (1) Extraction” to undergo macro-dissection (if required) and DNA extraction |
|  | - Refer to the case Snapshot to look up surgical pathology ID number - “Referring Institution Case ID” is for outside cases – line should be deleted for Unity cases - Estimated tumour % will be provided by reviewing pathologist(s) |
|  | Following data fields shown on the left are to be entered upon receipt   - “tumour_origin” = “CNS” for most cases, unless indicated otherwise - Use “.td” in text fields to enter today’s date - Use “comment_assay” field for internal commenting texts |
|  | - Assay results are entered at bottom of the screen - Refer to the resulting form to track results for batch - “mgmt_fam” & “mgmt_vic” copy numbers entered, corresponding to *MGMT* (methylated) and *ACTB* copies, respectively - “mgmt_percent” - entered as % (i.e., “1.0” for “1.0%”) - data field does not accept “%” symbol - “mgmt_hegi” & “mgmt_hegi_ajd” can be left blank – to be calculated by molecular pathologist - Please add “internal comment” to alert pathologists – ex. low *ACTB* copies |
|  | - Enter “Assay Results” by choosing from the drop-down menu - Ensure “Material Information” is filled correctly - “Complete Step” to place the case for sign-out by attending   Once all results have been entered, e-mail the molecular pathologist for review and sign-out. |

### MGP – *MLH1* promoter methylation

|  | Ordering “MLH1” on a block will pull up molecular study order tasks as curls or unstained slides   - Choosing slides automatically orders a set of 10 unstained slides, H&E slide, and the MLH1 trigger |
| --- | --- |
|  | - Extract DNA and enter the information as above, including Qubit-derived DNA concentration |
|  | PCR results are entered:   - “regc” = “Region C” - “regd” = “Region D” - "b2m” = Ct values obtained for the beta-2-microglobulin gene - “per” = % methylated - “per_adj” = % methylated, adjusted for tumour %   Once all results have been entered, e-mail the molecular pathologist for review and sign-out. |

### MGP – JAK2 p.V617F assay (MutaScreen)

|  | Specimen receiving – please enter:   - “order_type” (routine *vs.* STAT) - “specimen_received” - blood or bone marrow - “sample_class” = clinical - “sample_type” - in-house, outside, etc. - sample_osh – enter outside hospital information as appropriate (IDs)   NOTE: order is not currently available at the SJHC site – only available at SMH (see send-out option instead) |
| --- | --- |
|  | DNA extraction   - storage_location = location of extracted DNA storage |
|  | PCR assay   - "assay_lot” - “jak2_fam” = average copy numbers - “jak2_vic” = average copy numbers - “jak2_ratio” = based on averages - “jak2_gz_low” & “jak2_gz_high” = grey zone values for run batch |
|  | Resulting:   - Choose from drop-down value - Enter JAK2 comment (“UNITY LAB AP JAK2 COMMENT”) and edit as appropriate   Once all results have been entered, e-mail the molecular pathologist for review and sign-out. |

- Check workflows are valid for PBs

- Check workflows for bone marrow samples

### MGP – PRMU/FVL

NOTE: Below steps were based on the legacy PRMU/FVL assays. The assays have been paused prior to go-live date. The below information is for reference only.

|  | Ordering may be performed from clinics/wards - access encounter and “Add Order” from “Patient Station”   - Concurrent ordering "Prothrombin G20210A Mutation” and “Factor V Leiden Mutation” automatically results in linking of the two test orders |
| --- | --- |
|  | New orders appear in “Chart Review” under “Labs” |
|  | Tests ordered can be viewed under “Molecular Pathology (PCR)”   - PRMU & FVL orders will have two separate specimen IDs - Extract one tube – use both labels on same tube - “Container sharing” function to be explored in future - Workflow steps can be completed for multiple samples simultaneously |
|  | Click on a case on the worklist to pull resulting screen |
|  | The resulting screen various components of the report, including the pre-defined texts (disclaimer, methodology, references). |
|  | Clicking on the pencil icon enters edit mode.  Choose the appropriate result category |
|  | If the test was rejected, the comment text must be modified. Delete the pre-populated comment in the Comment box, and click on “Insert SmartText”. |
|  | Choose the comment “FACTOR V LEIDEN MUTATION – HDSC" to choose the comment text.  Click “Accept” to insert the comment. |
|  | Once the correct results are entered, click on “Pend Final” to send |
|  | Tests not indicated – deletes disclaimer, methodology & reference canned texts |

- Test approval
  - Samples held - Buffy coat stored (2+ months)
  - Aliquot to be stored in –80C at hematology
- APCR > 2.12 - calls mnemonic FVL not indicated
  - To be setup as auto-verify
  - Samples held – 1+ year
  - APCR run every week, usually
  - FVL testing-related data will be deleted – press “Warning Acknowledged” and Final Verify results
- LAB7530 for FVL – EAP in Beaker

### MGP – NGS (UHT somatic tumour molecular profiling assay)

|  | Ordering NGS is accessed through “Task Protocols”, ordered on a block. Choose "NGS-Molecular Slides”   - The order set results in 10 unstained sections + H&E level being ordered, accompanied by “NGS trigger”, which starts the workflow below |
| --- | --- |
|  | NGS workflow is broken down by workflow steps  In Chart Review, NGS test is listed as “Molecular Sequencing Request” |
|  | When samples have been received, enter specimen information as appropriate:   - Order type = routine/STAT - Sample class = clinical, EQA, validation, or research - Sample type = in-house, referred, etc. - Material Information = case ID, block ID, material type (FFPE), tumour %   To be entered by pathologists:   - tumour_tissue = tissue source - tumour_type = carcinoma, melanoma, etc. - tumour_origin = primary site (or most likely site)   NOTE: Use “Tracking” function rather than entering all sample receiving information.   - Date, time received, person receiving do not need to entered, if tracked |
|  | Extraction information   - DNA_qubit = DNA concentration measured by Qubit - DNA_qubit_lot = lot information for the Qubit assay - storage_location = freezer box desgination - comment_sample = internal information for comments regarding the sample |
|  | NGS resulting – enter, as generated from Genoox report template   - Tier I/II variants - Point Mutations Identified – additional infromation for tier I/II variants - AMP/ASCO/CAP TIER III Gene Variants (variants of unclear/unknown clinical significance) - Sample Quality Control Metrics - “drop-out” regions   Also enter “Case Summary” - recapitulation of the overall NGS results, clinico-patho-molecular correlations |
|  | Standard disclaimer, methods & reference sections genes are automatically populated   - Includes gene list (as table) - reference transcripts are designated in the table - Reference sequence (hg19) is indicated in “Methodology” |

### MGP – In-house microbiology testing

|  | In Case Builder, order sections as:   - Slides – ordered as “Molecular Curls (Micro Mol)” |
| --- | --- |
|  | Provide sample to the path admin team.  Requisition Entry > Choose “HERPES SIMPLEX VIRUS (HSV) 1 AND 2, VARICELLA ZOSTER VIRUS (VZV) PCR”   - Make sure “Code” = “LAB4070” - Specimen type = “Tissue” - Priority = Routine - Designate “Specimen source “   Print label and stick onto materials & deliver to microbiology (UHT) |

### MGP – Sendout molecular tests

|  | Direct orders (e.g., UHN JAK2 workup) can be ordered through usual ordering process   - Be sure to choose “Facility List” when looking for test - “Preference List” - individual-user set; shows subset of orders available for encounter type |
| --- | --- |
|  | For send-out tests where curls/unstained slides are cut from UHT histology lab, order task protocols designated as “(Sendout)” (curls or slides). If needed, be sure to include H&E slides. |
|  | Provide case to path admin for sendout – indicate desired test.  Open “Sendout Bench” for specimens already in the lab   - Click “Packing List Editor” - Indicate desired test/institution, and material to be sent (see packing list editor section) |

### MGP – QA/QC/QM

MGP QA/QC/QM will continue using the various manual spreadsheets and paper forms currently in use – this function will be re-visited for Epic Beaker optimization

## USER SETTINGS

Any user customization needs to be completed in the real production (PRD) environment. Please note that the PRD environment has real patient data – when completing user customization, only use the designated *ZZZtest* patients.

### User Settings – General

|  | “... (action)” -> Personalize   - View menus available - Automate List Switching – automatically enters next case on outstanding list |
| --- | --- |
|  | Dictionaries can be customized by:   1. Type word 2. Right click 3. Dictionaries 4. Re-type in word & choose options – ignore, autocorrect, etc. - misspelled will flag words as such  - case sensitive for certain functions - Autocorrect helpful for short texts (*vs.* longer texts for varous smart tools) |

### User Settings – Creating SmartPhrases

- 1. Find the name of the note template you want to update for yourself. For example, click the icon in the “Insert SmartText” window in the sidebar of your open note and write down the name of the SmartText you want to change.
- 2. Click My Tools on the main toolbar, and then select My SmartPhrases. Or use Chart Search to open My SmartPhrases.
- 3. Create a new SmartPhrase and give it a short, intuitive name.
  - You might want to begin your phrases with your initials. For example, if your initials were N.J., you might name the SmartPhrase "NJPROGNOTE.“
- 4. In the Insert SmartText field, enter the name of the note template you recorded in step 1.
- 5. Adjust the details of the note template as needed.
  - You can pull SmartLinks into your note template by typing a period followed by the name of the SmartLink, with no spaces. The SmartLink appears in your template surrounded by @ symbols.
- 6. Add a brief summary of your SmartPhrase in the Description field. When you search for your SmartPhrase, this description appears next to the name.
- 7. Click Accept when you're done.
  8. To use your new SmartPhrase, type a period followed by the SmartPhrase name, and then press Spacebar.
- Add speed buttons to jump into documentation
  - 1. Click in the upper-right corner of the Notes sidebar, activity, or navigator section.
  - 2. In the window that appears, enter a Note Type you want to create a speed button for.
  - 3. Search for a SmartPhrase or SmartText.
    - * Tip: The Default Preferences you see allows you to default your own created note into the note text field when you open a New Note.
  - 4. Click Add to add it as a new button.
    5. In the Caption field, enter the name that you want to appear on the button.
    6 .Click and drag the buttons to re-order them (if you have more than 1 speed button already created)
    7. Click Accept. The button appears at the top of your Notes section.

### User Settings – Creating new lists (EMR function)

| Creating new list of patients  1. Click Edit List > Create My List.  2. Name your list. We recommend prefacing the name with your initials.  3. Click Copy and select a template with the columns you want.  4. Rearrange the orders of the columns to suit your needs by clicking  5. Delete any columns you don't want by clicking Remove.  6. Add an individual column by selecting it from the Available Columns menu and clicking Add Column.  7. Add your group's patients to the list: Under Available Lists, right-click your group's list and select Send To > |
| --- |

- Organize your Orders Preference List
  - 1.Use Search to open the Preference List Composer.
     2.In the list that appears, double-click your Orders (inpatient) preference list.
    - It's important to choose the orders preference list because when you place an order and save it as a favorite, the order is automatically added to this list.
  - 3.Click New Section.
    - Create sections that are appropriate for your specialty and that group orders you often place at the same time.
    - Enter a display name that makes sense to you.
    - Indicate whether you want orders in this section to appear alphabetically when browsing the list.
  - 4.To add a subsection, select the section it should appear under and click New Subsection.
    - Add subsections that group orders appropriately for your specialty. or example, you might add subsections for meds, labs, and imaging to various disease/diagnosis sections.
    - Reorganize the sections and subsections in your preference list by dragging and dropping them or by using the Section Up and Section Down buttons in the bottom left.
  - 5.Click X in the upper-right corner when you're finished adding sections.
- Create Your Own Version of an Order Set - If you often need to change details in an Order Set, you can create your own version of the Order Set, called a User Order Set. After you open a User Order Set for a patient, you can further customize the details for that patient.
  - 1. Open an Order Set that you want to customize.
  - 2. Click Manage User Versions and select Create My Version.
  - 3. Enter a name that will help you remember this version of your Order Set.
    - For example, enter Mild Pneumonia. The version name you enter appears only to you and not to other providers, so enter a name that makes sense to you.
  - 4. Click the order details link to customize details such as the dose, frequency, and rate.
    5. Select or clear the check box next to an order to customize which orders are selected automatically.
  - 6. Click Accept. Your User Order Set is now open for you to use with the current patient and available from your favorites list for easy access in the future.
- Create Your Own Order Panels
  - 1. Within a patient's chart, search for the orders that you would like in the order panel.
  - 2. After the orders are queued up for signing, click Options and select Create Panel.
  - 3. In the Display name field, enter an easy-to-remember name for the panel.
    - If needed, edit the details of each order.
  - 4. Click Accept.
  - 5. To use your order panel in a patient's chart, search for the order panel on your preference list by completion matching or browsing, just as you would for an individual order.
- Extra time? Edit or make a copy of an order panel - After you've created an order panel, you can update order details or remove orders from it, and make a copy.
  - 1. Open your preference lists and select the Only Favorites check box.
  - 2. Right-click the order panel.
    - To edit the current order panel, select Edit Preference List Entry.
    - To create a copy of the order panel, select Add to Preference List and choose a list to put the copy on.
  - 3. Add an order, edit order details or remove orders.
- Create a macro to record common findings
  - 1. After making your selections on the ROS, Physical Exam, or Procedures tab of the NoteWriter, click to the right of the pencil icon (drop-down arrow) and select Create macro from current data.
    2. In the Macro Selection window, enter a name for your macro and click Accept.
    - Note any age and sex restrictions that appear. For example, the macro you create might apply only when you're doing a review of systems for females age 13 and older. These restrictions allow you to create different macros for female, male, and pediatric patients.
  - 3. In the SmartBlock Macro Editor, select any other items that you commonly record. Then select the Released check box (make sure its selected) and click Accept. Your macro is now available for use.
    - Note that any additional changes you make in the editor are not automatically applied to your note for the current patient.
  - 4. To apply this macro at a future visit, click the button that appears next to your new macro at the top of the NoteWriter toolbar
- Customizing Schedule
  - 1. Go to your schedule. Click the name at the top left of your schedule. In the window that appears, select a schedule under the My Schedule group.
  - 2. Click the gears to customize your schedule.
    - To reorder your columns, select one from the Selected Columns list and click the arrows to move it.
    - To add a column, select it from the Available Columns list and click Add Column. For example, select the Meds Due column to see whether the patient has a medication due, or select the Referring column to see the referring provider for the visit.
    - To remove a column, select it from the Selected Columns list and click Remove. For example, remove the Provider column if your schedule lists only your visits.
  - 3. Click Accept when you're finished.

## SUPPORT RESOURCES

### Support Resources – Help Resources

|  | Click on the Question mark of press “F1” (on PCs and not Macs) to open Pathologist Learning Home |
| --- | --- |
|  | Document can also be viewed separately at : <file://epicfsnp/EpicSharesNP/Application/LearningHomes/Beaker/Anatomic%20Pathology/UHT%20Pathologist%20-%20Sign%20out%20a%20Case%20QSG.pdf>   - February, May, August, November are release dates for regular Epic version updates – principal trainers will review updates |

Also contact Helpdesk (44357) - option 3 for virtual at-the-elbow (ATE) support.

### Support Resources – Tickets

Epic-specific process will be in-place. To consolidate the tickets, pathology users are asked to coordinate with designated support persons – Matt and Jong.

### Support Resources – PLY (playground) environment

Playground (PLY) is a copy of the training environment.

- refreshed every night, practice patient data are removed, so that each day there is a clean slate
- Epic ACE and Playground environments undergo a nightly refresh at approximately 10:00 PM - during this process, all patient data and documentation will reset, ensuring a clean environment for the next day

To access PLY: Go to https://citrix.unity.local/CItrix/AppsWeb/ and log-in using your personal Unity Health Toronto credentials. (see https://unitynet.unity.local/wp-content/uploads/2024/09/PLY-Login.pdf)

## ELLKAY

### Ellkay – General

|  | Ellkay is directly from Epic, from the Chart Review view.   - The document may alternatively be labelled as “Documents and Archive”   NOTE: Scanned document is stored only once (in ELLKAY) but it should be easy to get to from Epic via Chart Review |
| --- | --- |
|  | New tab labelled “Web Viewer” opens, within the Epic platform.  NOTE: Direct URL address will not be used post go-live (https://cas1.lkemrarchive.com/UHT1009-LIS/) |

|  | |
| --- | --- |
|  | PDFs are available under “Lab Result” |
|  | More of the result components can be viewed as individual components under “Case Results” |

- Search for under MRNs or other identifier

|  |
| --- |

- Case/specimen information, including protocol – found under “Specimen” tab > “Comments”

|  |
| --- |

- Retrieval flag information under “Case Requisition”

|  |
| --- |

### Ellkay – Viewing results

|  | Enter MRN  Click to go into the case |
| --- | --- |
|  | Click on the most recent version of the report (PDF icon) |
|  | View “Case Results” to view each data field separately – click on “Observation Notes” |

### Ellkay – Additional information

|  |  |
| --- | --- |
|  | Encounter information can be found for the case – this information was not validated during data migration validation. |
|  |  |

## Trainees

Login

|  | When you login, ensure that your “Application” is set to Beaker. “EpicCare” using the resident template, is the wrong application for pathology trainees. |
| --- | --- |
|  |  |

## ANALYTICS

### Analytics – Pathologist Dashboard

|  | Dashboard |
| --- | --- |

### Analytics – SlicerDicer

|  | Access through Epic>Cogito Menus>SlicerDicer  Can also search “a cat” from the Epic button search to run reports – choose SlicerDicer |
| --- | --- |
|  |  |

### Analytics – OR list

|  | Go to Analytics Catalogue - if accessing for the first time, use the search bar |
| --- | --- |
|  | In the Catalog screen, ensure all filters are checked off, and search for "OR" |
|  | Look for the "OR Projected Case Start by Hour (Today & Tomorrow)"   - also available are "OR On Time Starts (Today) for Dashboard", "OR On Time Starts (Yesterday) for Dashboard", and "OR On Time Starts" - Click to start the report |
|  | Use the "Detail List" to view the list in a tabular format   - - other tabs can be explored, as well as access to case & chart info |
